# Toward Polaritonic Molecular Orbitals for Large Molecular Systems

**DOI:** 10.1021/acs.jctc.4c00808

**Published:** 2024-09-30

**Authors:** Yassir El Moutaoukal, Rosario R. Riso, Matteo Castagnola, Henrik Koch

**Affiliations:** Department of Chemistry, Norwegian University of Science and Technology, 7491 Trondheim, Norway

## Abstract

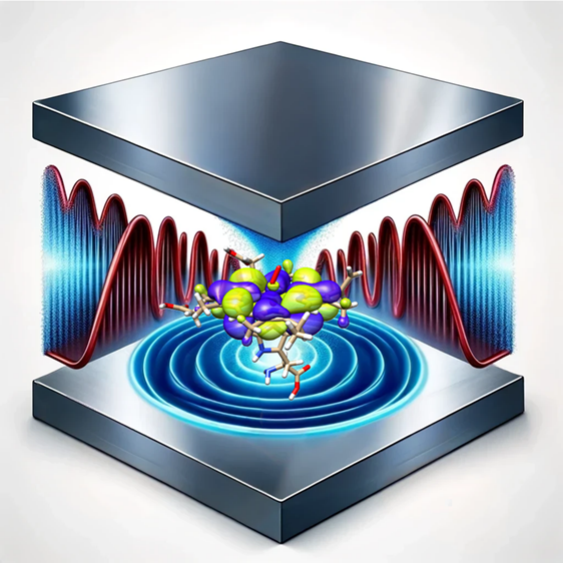

A comprehensive understanding of electron–photon correlation is essential for describing the reshaping of molecular orbitals in quantum electrodynamics (QED) environments. The strong coupling QED Hartree–Fock (SC-QED-HF) theory tackles these aspects by providing consistent molecular orbitals in the strong coupling regime. The previous implementation, however, has significant convergence issues that limit the applicability. In this work, we introduce two second-order algorithms that significantly reduce the computational requirements, thereby enhancing the modeling of large molecular systems in QED environments. Furthermore, the implementation will enable the development of correlated methods based on a reliable molecular orbital framework as well as multi-level methodologies able to model the inclusion of solvent effects in this kind of complex systems.

## 1 Introduction

Strong coupling between electromagnetic vacuum fluctuations and molecular systems has been suggested to be an innovative way to noninvasively engineer molecular properties.^1−5^ To effectively achieve light-matter strong coupling, different optical devices able to spatially confine electromagnetic fields have been designed.^6−10^ The strong coupling regime is unlocked once the coherent energy exchange rate between the electromagnetic field and the molecular system exceeds the dissipation processes.^11−13^ This interaction leads to the formation of polaritons, which mix photonic and molecular degrees of freedom.^14−17^

While new experimental studies keep increasing the range of possible applications,^18−22^ a complete rationalization of the mechanisms behind these modifications is missing, underlying the pressing need for theoretical insight into the complex interplay between light and matter.^23^ In this regard, ab initio methods that model the underlying physical processes starting from wave functions are of the utmost importance to faithfully reproduce the molecular features of the polaritons. The electromagnetic fields and matter degrees of freedom must be treated on the same footing by means of quantum electrodynamics (QED) theory.^24^ Several ab initio models have been proposed in the past few years to capture electron-photon correlation while keeping a polynomial scaling describing the overall complexity. Most of the well-established quantum chemical approaches have been extended. More specifically Hartree–Fock^25^ (QED-HF), density functional theory^26,27^ (QEDFT), as well as coupled cluster^25,28−30^ (QED-CC), full configuration interaction^25^ (QED-CI), complete active space configuration interaction^31^ (QED-CASCI) and Mo̷ller–Plesset second order perturbation theory^32^ (QED-MP2). However, one also encounter instances where the extension of the quantum chemical concepts is nontrivial, like in the case of polaritonic molecular orbitals.

Molecular orbitals are powerful theoretical tools able to provide a description of molecular properties, for instance, the rationalization of stereoselectivity in chemical reactions. In quantum chemistry, the molecular orbitals are obtained by solving the Hartree–Fock method. However, the orbitals obtained from the straightforward generalization of Hartree–Fock to cavity environments (QED-HF) has unphysical features, notably they do not display correct intermolecular consistency and they are not origin invariant for charged systems. A polaritonic molecular orbital theory is necessary to address these issues and provide a more accurate description of the molecular behavior under strong light-matter coupling. Several groundbreaking works have indeed demonstrated that strong light-matter coupling can change both the ground and excited state reactivity, altering the reaction kinetics,^33^ changing reactive yields, and even affecting the selectivity toward a particular product.^34^

Recently, Riso et al.^35^ presented the strong coupling QED Hartree–Fock (SC-QED-HF) model, the first fully consistent molecular orbital theory for QED environments. The approach is very promising not only because it can be used to rationalize how molecular orbitals are reshaped, but also because it can represent a valuable reference for the development of more accurate correlated approaches. Despite its potential, the first implementation in  program^36^ has convergence difficulties that restrict its applicability. These numerical limitations find their roots in the multicomposite nature of the wave function parametrization, which includes two classes of parameters, one accounting for the orbitals optimization and one for the electron-photon interaction. Simultaneous optimization of these two physically different variables negatively affects the convergence. In this work, we tackle this issue by developing two second-order algorithms. The new algorithms significantly speed up convergence. These results pave the way for developing correlated methodologies and significantly increase the application range for large molecular systems.

This paper is organized as follows: in Section 2, we present a brief overview of the SC-QED-HF theory, highlighting differences between the previous implementation and the new algorithms. In Section 3, we demonstrate the improved convergence with a set of benchmark molecules that includes organic as well as inorganic ones. Thereafter, we discuss the computational scaling of the improved methodology. In the Section 4 we present our conclusions and future perspectives.

## 2 Theory

In this work, the light-matter interaction inside a cavity with quantization volume *V* is modeled using the Pauli–Fierz Hamiltonian in the length gauge and dipole approximation, where only one effective cavity mode is considered^37−39^
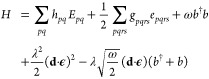
1In eq 1, the bosonic operators *b*^†^ and *b* respectively create and annihilate a photonic mode of the cavity with frequency ω. The light-matter interaction is mediated through the photonic-bilinear term where **ϵ** is the polarization vector of the field, λ is the coupling strength
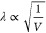
2while **d** is the molecular dipole operator defined as
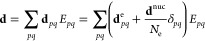
3with **d**^e^ being the electronic dipole and **d**^nuc^ the nuclear dipole of a system of *N*_e_ electrons. The electronic operators *E*_*pq*_ and *e*_*pqrs*_ are given by
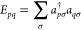
4
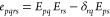
5where *a*_*p*σ_^†^ and *a*_*p*σ_ are the creation and annihilation operators for an electron in orbital *p* and spin σ. Finally, **d**_*pq*_^e^, *h*_*pq*_ and *g*_*pqrs*_ are the one and two electron integrals that enter in the Pauli–Fierz Hamiltonian. We note that the dipole self-energy (DSE) term ensures the Hamiltonian in eq 1 is bounded from below.^40^ For simplicity, in the remaining part of this work, the ∼ symbol denotes integrals and operators in the basis that diagonalizes the integrals (**d**·**ϵ**):
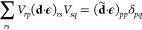
6
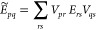
7where **V** is an orthogonal matrix. The dipole basis is particularly suitable in the strong coupling regime as Slater determinants in that specific basis are the exact eigenstates for the Pauli–Fierz Hamiltonian in the infinite coupling limit.

### 2.1 Strong Coupling QED Hartree–Fock

The SC-QED-HF method is the first QED ab initio framework able to provide origin independent molecular orbitals in a nonperturbative treatment that capture cavity frequency dispersion as well as being intermolecular consistent.^41,42^ In this approach, the wave function reads
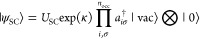
8where *U*_SC_ is the strong coupling transformation
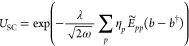
9where {η_*p*_} are orbital specific coherent state parameters. The electronic and photonic vaqua are referred respectively to as |vac⟩ and |0⟩. The wave function in eq 8 becomes increasingly accurate as λ → *∞* because it is obtained by relaxing the infinite coupling solution to a finite strength (see Supporting Information for a detailed derivation). The molecular orbitals are optimized through a unitary transformation^43,44^ exp(κ), where
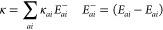
10and *a* and *i* denote virtual and occupied MOs. Unlike the uncorrelated QED-HF model, the SC-QED-HF theory incorporates electron-photon correlation by dressing the electronic molecular orbitals with the photonic degrees of freedom as seen from
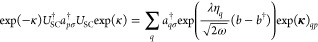
11In the dipole basis, the Pauli–Fierz Hamiltonian can be written as
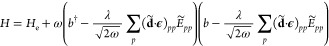
12and transforming it with *U*_SC_, we obtain
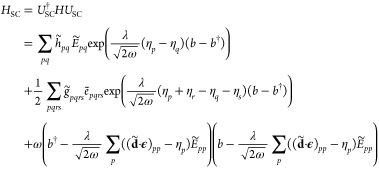
13This Hamiltonian differs from the Pauli–Fierz operator in eq 12 by the η-shifting of the dipole integrals and the photonic dressing of the electronic terms. The optimal wave function is determined by energy minimization using the gradients with respect to the parameters:
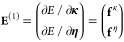
14where *E* = ⟨ψ_SC_|*H*|ψ_SC_⟩ and ⟨ψ_SC_|ψ_SC_⟩ = 1.

Now we obtain the gradient with respect to η_*r*_ as
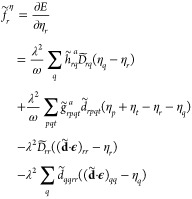
15where
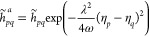
16
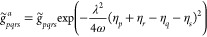
17are the one and two electron integrals scaled by the ω-dependent Gaussian factors. The density matrix elements are given by *D̃*_*pq*_ = ⟨HF|*Ẽ*_*pq*_|HF⟩ and *d̃*_*pqrs*_ = ⟨HF|*ẽ*_*pqrs*_|HF⟩. The gradient with respect to *D̃*_*pq*_ equals the SC-QED Fock matrix element in the dipole basis
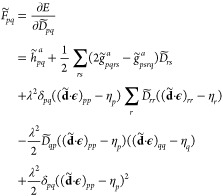
18which is related to the nonredundant Hartree–Fock gradient in the canonical basis:
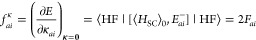
19where ⟨*H*_SC_⟩_0_ is the vacuum averaged SC-transformed Hamiltonian and
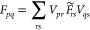
20The origin dependence of the QED-HF orbitals stems from the changes that a displacement **a** has on the dipole operator of a charged molecule
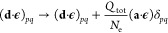
21where *Q*_ tot_ is the total charge of the system. For SC-QED-HF, this change in the molecular dipole can be reabsorbed through an appropriate shift in the η parameters, leading to an origin invariant Fock matrix and thus orbitals.^35^

In the previous implementation, the orbital optimization is performed using the Roothaan-Hall self-consisted field (SCF) procedure^45,46^ where diagonalization of the Fock matrix is performed coupled with the direct inversion in the iterative subspace (DIIS) algorithm.^47,48^ Meanwhile, the η-parameters were updated in a steepest descent fashion accelerated by the DIIS. The numerical difficulties of the previous implementation stem from the inability to provide a proper preconditioner in the η update. Specifically, optimizing the density matrix exhibits similar behavior as standard Hartree–Fock, while the gradient in the η-parameters fail to predict a reliable convergence path. A partial solution to this issue is performing very small steps in the {η_*p*_}, but this significantly increases the calculation time.

It is well-known in numerical optimization that more reliable convergence paths can be found using higher derivatives.^49^ In the next two sections, we present two new optimization schemes exploiting parts of the Hessian matrix to improve convergence stability and speed.

### 2.2 Trust Region Newton–Raphson Optimization

The construction of the Hessian matrix allows us to understand how closely different parameters are interrelated by capturing the curvature of the parameter hypersurface. Using the second-order derivatives, a Newton–Raphson type algorithm can be developed.^50^ Since the wave function is composed of two different classes of parameters, κ and η, the Hessian matrix contains four different blocks
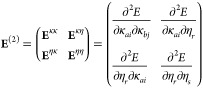
22The individual blocks are obtained from the following expression
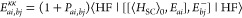
23
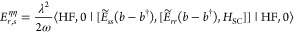
24
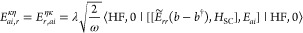
25For each block, the derivatives are calculated at **κ** = **0** because the Hessian is computed in the updated MO-basis. Instead, for the η-parameters, the derivatives are evaluated at the present values. For the explicit derivation of the Hessian blocks, we refer to the Supporting Information. Moreover, by employing a trust region (Levenberg–Marquard) approach, it is possible to enhance the robustness of the optimization by constraining each new step to stay within a trusted neighborhood of the previous iteration.^49,51,52^ The optimization is carried out by solving in each iteration
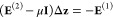
26featuring the level-shifted Hessian (**E**^(2)^ – μ**I**), the new step Δ**z** and the gradient vector **E**^(1)^. For the derivation and the computational details of the trust region Newton–Raphson algorithm, we refer to the Supporting Information. To compute the new step, one could invert the level-shifted Hessian in eq 26, but this approach is computationally demanding due to the large number of the nonredundant κ-parameters. For this reason, we solve the linear system iteratively only calculating the action of the Hessian on a trial vector:
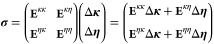
27This linear transformation approach is particularly efficient because each term in eq 27 can be expressed in terms of gradient elements. Moreover, our numerical investigations reveal that neglecting the mixed blocks of the Hessian gives a robust and faster converging algorithm. This implies that the coupling between the two sets of parameters is not particularly tight and relevant for the optimization. In this case, the linear transformation simplifies to
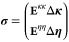
28For the purely κ–κ linear transformations, we have the Hartree–Fock equation in terms of the κ-gradients
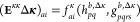
29where the redefined one and two electrons integrals
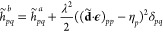
30
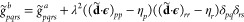
31are rotated back to the canonical basis and then Δκ-transformed as follows:
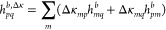
32
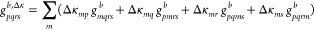
33On the other hand, for the purely η–η linear transformations in terms of the η-gradients we have
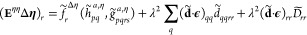
34where the η-transformed one and two electrons integrals are
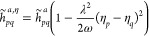
35
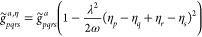
36For the explicit derivation of the linear transformations comprising also the mixed parameters ones we refer the reader to the Supporting Information.

### 2.3 Direct Inversion of the η–η Hessian Block

The trust region Newton–Raphson approach requires an iterative algorithm in order to solve the linear equations that determine the step length. However, as shown in Section 3, the gradient based algorithm only struggles with the optimization of the η-parameters. This suggests an alternative algorithm where we only use DIIS acceleration for the density matrix and Newton–Raphson for {η_*p*_} obtained from the direct inversion of the η–η Hessian block:
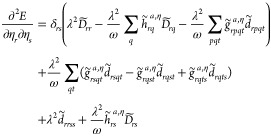
37The computational cost of building the η–η Hessian matrix is *N*^4^ and the matrix inversion is *N*^3^, in this way recalculation of the integrals is avoided.

## 3 Results and Discussion

**Figure 1:**
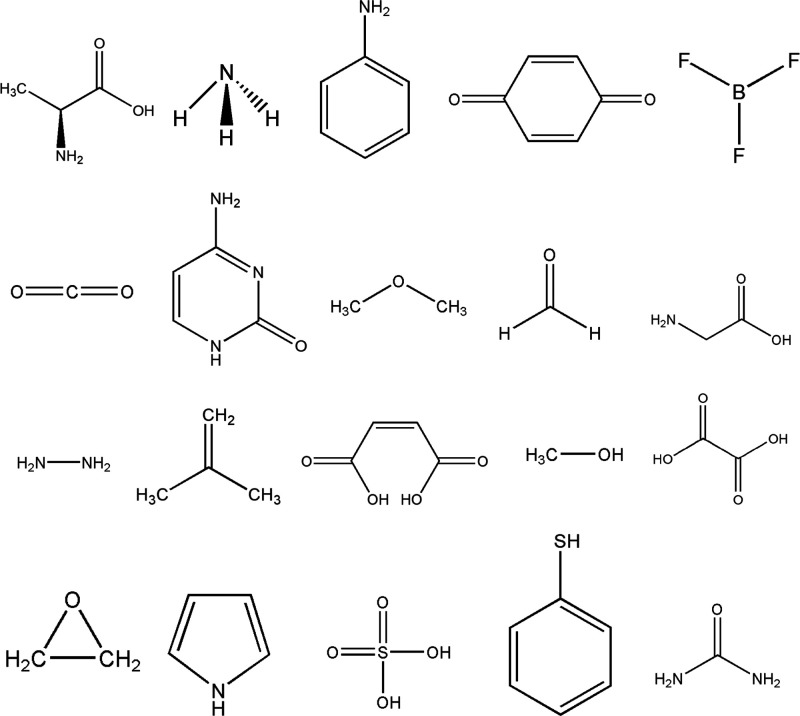
Benchmark molecules.

In order to demonstrate the computational efficiency of the developed algorithms, we compare the performance for the set of 20 molecules shown in Figure 1. We also present a few calculations for larger molecular systems using the batching algorithm of the Cholesky decomposed two-electron integrals. All the calculations have been performed with a development version of the  program^36^ using a dual-socket Intel(R) Xeon(R) Platinum 8380 system with 2 TB of memory. In the benchmark study we employed 20 cores, while for the largest systems 80 cores were used.

### 3.1 Benchmark of the Methods

**Figure 2:**
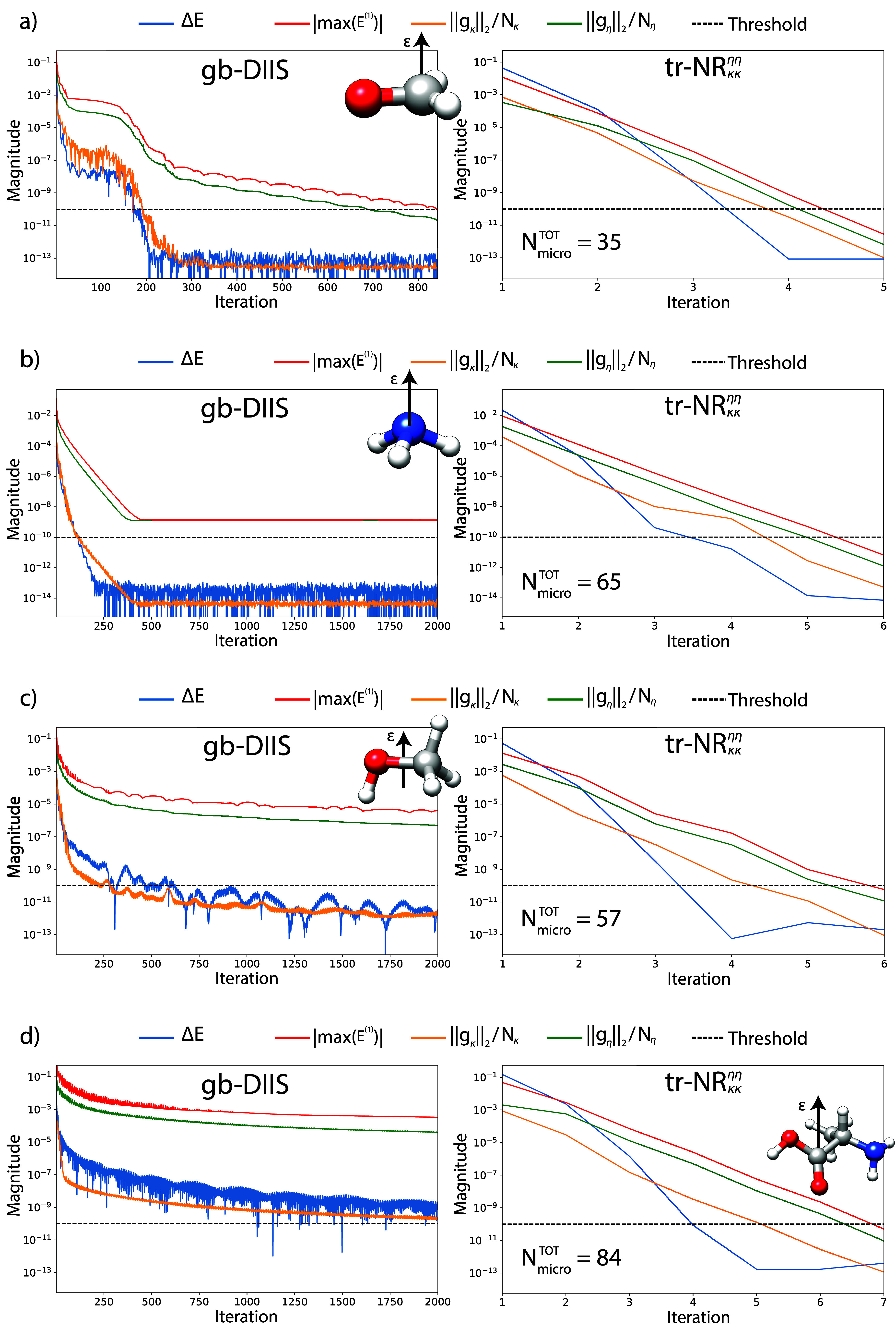
Convergence comparison between the gb-DIIS and the tr-NR_κκ_^ηη^ algorithms for (a) formaldehyde, (b) ammonia, (c) methanol, and (d) alanine. For the tr-NR_κκ_^ηη^ algorithm, we also report the total number of microiterations. See text for the definition of the other quantities.

In Figure 2, we report the comparison of the convergence patterns for the DIIS accelerated gradient-based implementation (gb-DIIS) and the trust region Newton–Raphson algorithm using only the κ–κ and η–η blocks of the Hessian (tr-NR_κκ_^ηη^). For conciseness, we illustrate the convergence only for formaldehyde, ammonia, methanol, and alanine. All molecular geometries and the results for the remaining 16 molecules are reported in the Supporting Information. We used an aug-cc-pVDZ basis set,^53,54^ light-matter coupling λ = 0.005 a.u. (atomic units), vacuum cavity frequency ω = 2.71 eV, and a field polarization along the *z*-axis. The quantities plotted for each iteration are the absolute energy difference from the previous iteration
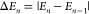
38the absolute maximum value of the total gradient vector |max(**E**^(1)^)|, and the *L*^2^-norms of the κ and η-gradients (defined in eqs 15 and 19) scaled by the number of nonredundant parameters within each class: ||**g**_κ_||_2_/*N*_κ_ and ||**g**_η_||_2_/*N*_η_. For all calculations, the convergence threshold is set to 10^–10^ a.u. The results obtained with the gb-DIIS implementation are shown to the left in Figure 2 and clearly indicate that the convergence pattern of the η-parameters is not optimal. This is also corroborated by the rapid convergence observed when optimizing the orbitals while keeping the η-parameters frozen to the eigenvalues of the dipole operator.

For the gb-DIIS algorithm, only formaldehyde converges within 2000 iterations as can be seen in Figure 2a. For ammonia and methanol, in Figure 2b,c, the scaled *L*^2^-norms of the η-gradient reach a plateau of 10^–9^ and 10^–6^ a.u., respectively. The κ parameters keep oscillating around their stationary value, with the energy slowly decreasing by 10^–11^–10^–14^ Hartree in each step. On the other hand, for alanine in Figure 2d, we observe that not even the κ-parameters are converged within 2000 iterations, while the η-parameters reach a plateau much higher than the convergence threshold. In Figure 2, to the right, we show the results obtained with the tr-NR_κκ_^ηη^ algorithm. In all four cases, convergence is reached in less than 10 iterations, where each iteration requires on average less than 10 microiterations to solve the linear equations in eq 28.

**Figure 3:**
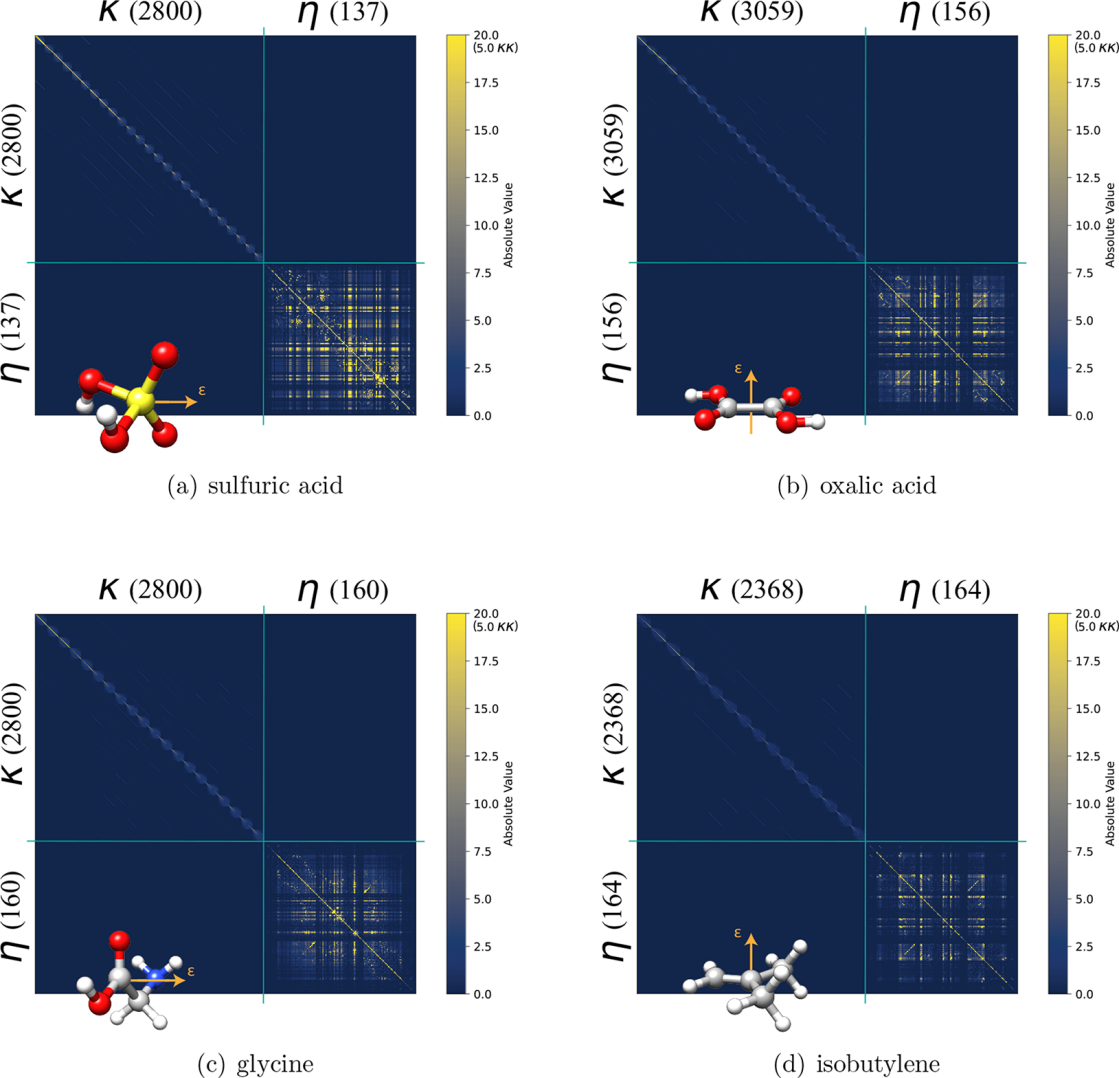
Hessian matrices at the first iteration for (a) sulfuric acid, (b) oxalic acid, (c) glycine, and (d) isobutylene. η–η and mixed parameter blocks are resized to provide better visualization of these critical terms. Cutoff of 20.0 in the color scale is used to better illustrate the importance of the off-diagonal elements in the η–η blocks. Cutoff of the κ–κ blocks is placed at 5 to appreciate the diagonal dominance.

We notice a fast and robust convergence when neglecting the mixed parameters blocks of the Hessian matrix. To validate this, we analyzed the Hessian matrix, in the first iteration, for all the molecules in Figure 1. In Figure 3, we show the heat map representations of the nonredundant Hessians for sulfuric acid, oxalic acid, glycine, and isobutyene. See the Supporting Information for the heat map representations for the remaining 16 benchmark molecules.

As expected from Hartree–Fock theory, the κ–κ blocks are diagonally dominant with the contribution from Fock matrix elements being the dominating part (see Supporting Information for derivation):
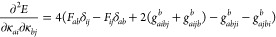
39In Figure 3 we observe that the η–η blocks are highly nondiagonal indicating the parameters are strongly coupled. Although the diagonal elements are larger than the off-diagonal ones (Supporting Information), the structure of the η–η block still leads to convergence difficulties of the gradient-based optimization. This also explains why considering these couplings in the tr-NR_κκ_^ηη^ algorithm benefits the procedure. The iterations saved by including the mixed blocks in the Hessian do not make up for the computational requirement (see the Supporting Information for a detailed wall time comparison).

The observations made from the Hessian analysis suggest the development of a third algorithm. To this end, the direct inversion of the η–η block is performed concurrently with the orbital optimization process of the original implementation (gb-DBI_ηη_). The plots with this algorithm are similar to the tr-NR_κκ_^ηη^ ones and are reported in the Supporting Information. In Table 1, we show the wall time for the three algorithms. We stress that the timings reported for the gb-DIIS optimization refer to achieving 2000 iterations while still being orders of magnitude far from convergence. Only formaldehyde converged in 843 iterations. The gb-DBI_ηη_ algorithm turns out to be faster in terms of wall time and number of iterations. These savings are obtained because the microiterations are no longer needed in favor of the direct inversion of the small η–η block.

**Table 1:** Wall Times and Iterations Comparison between the Algorithms

molecule	gb-DIIS	tr-NR_κκ_^ηη^ (micro-iter.)	gb-DBI_ηη_ (iter.)
alanine	7.71 h	22.0 m (84)	14.7 m (21)
ammonia	87.3 s	5.91 s (65)	1.81 s (14)
aniline	11.6 h	23.7 m (57)	21.9 m (20)
benzoquinone	10.2 h	25.1 m (69)	18.4 m (19)
boron trifluoride	19.4 m	69.1 s (76)	24.7 s (14)
carbon dioxide	6.38 m	20.2 s (73)	7.77 s (13)
cytosine	12.5 h	47.8 m (106)	27.4 m (23)
dimethyl ether	1.11 h	2.09 m (49)	87.3 s (15)
formaldehyde	2.40 m	10.2 s (35)	7.88 s (14)
glycine	2.87 h	6.94 m (71)	5.29 m (20)
hydrazine	15.4 h	26.8 s (44)	19.1 s (16)
isobutylene	3.41 h	6.37 s (51)	5.31 m (17)
maleic acid	10.2 h	36.2 m (104)	22.2 m (23)
methanol	12.1 m	31.2 s (57)	17.4 s (16)
oxalic acid	2.58 h	5.37 m (56)	4.58 m (19)
oxirane	35.1 m	83.3 s (69)	46.6 s (16)
pyrrole	2.83 h	5.23 m (47)	4.82 m (18)
sulfuric acid	1.68 h	8.76 m (145)	3.02 m (20)
thiophenol	10.0 h	26.0 m (72)	22.2 m (23)
urea	1.24 h	2.68 m (54)	2.16 m (18)

### 3.2 Polaritons for Large Molecular Systems

To investigate the polaritonic properties of larger molecular systems, we implemented a batching algorithm for the two-electron integrals in the dipole basis. While these integrals can be comfortably stored for smaller systems without significant memory requirements, larger systems need a more efficient handling. In our approach, the Cholesky vectors in the dipole basis are stored in memory, and the two-electron integrals are calculated on-the-fly in blocks that maximize the use of the total available memory:^55−59^
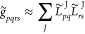
40

**Figure 4:**
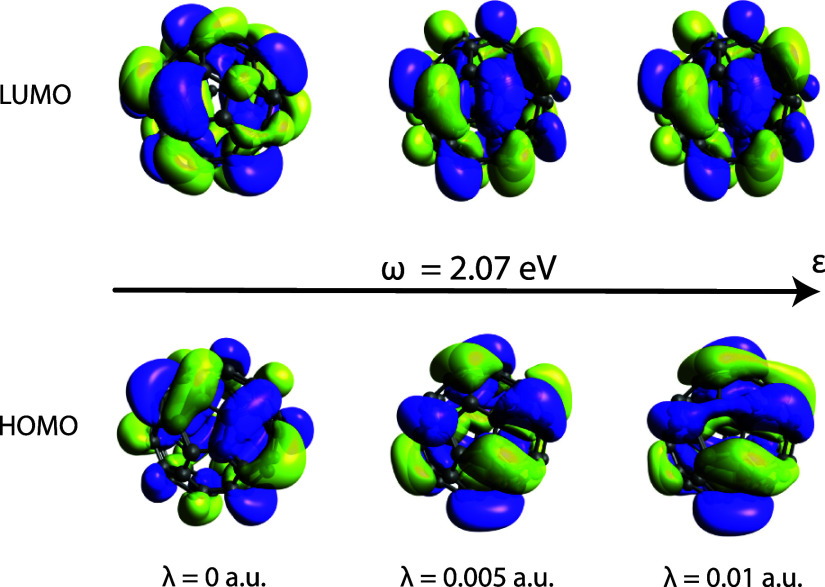
Fullerene (C_60_) HOMO and LUMO orbital reshaping at various couplings and cavity frequency set to ω = 2.07 eV. Arrow refers to the polarization vector. Surfaces are plotted using a 0.012 a.u. isosurface value.

In Figure 4, we show the reshaping of the HOMO and LUMO orbitals for fullerene using a cc-pVDZ basis set.^53,54^ In the Supporting Information we also show HOMO–1 and LUMO+1 orbitals as well as the molecular geometry. Interestingly we notice how the vacuum field polarization breaks the *I*_h_ point group symmetry and how this is reflected in the orbital shapes at various coupling strengths. For the selected cavity frequency these effects are more pronounced for the HOMO, while no significant changes are observed in the LUMO passing from λ = 0.005 a.u. to λ = 0.01 a.u.

**Figure 5:**
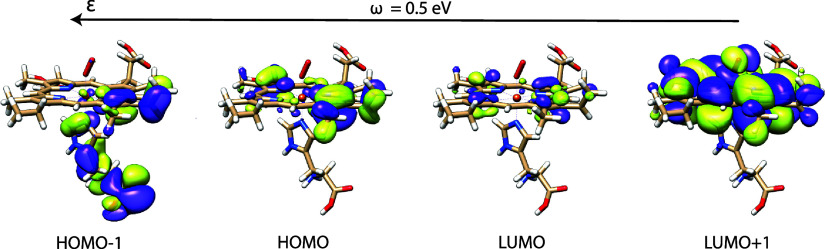
Surface differences between the λ = 0.01 a.u. and λ = 0 a.u. four frontier orbitals of a heme group coordinated to a proximal histidine amino acid and an oxygen molecule at ω = 0.5 eV. Arrow refers to the polarization vector. Surfaces are plotted using a 0.0002 a.u. isosurface value.

In Figure 5 we analyze the four frontier orbitals of the heme group with the Fe^2+^ ion coordinated to a proximal histidine amino acid and an oxygen molecule. We show λ = 0 a.u. and λ = 0.01 a.u. differences at cavity frequency ω = 0.5 eV. The molecular geometry is given in the Supporting Information. The calculations were performed using the tr-NR_κκ_^ηη^ algorithm for the first few iterations then followed by the faster gb-DBI_ηη_, due to the nonpositive definite Hessian in the early stages of the optimization. We used a 6-31G basis set^60^ without batching of the two-electron integrals. The observed differences in the orbitals are small due to the absence of cavity induced symmetry breaking.

Our results show that the improvements in the convergence will allow us to study large molecular systems and address the electron-photon correlation using a properly dressed set of orbitals that can be used in post-mean-field approaches.

## 4 Conclusions

In this work, we have reported a new and improved implementation of the strong coupling quantum electrodynamics Hartree–Fock model. Our new algorithms rely on the use of the second derivatives of the energy with respect to the wave function parameters. This provides faster convergence of the orbital-specific coherent state η-parameters. While a full implementation of the trust region Newton–Raphson scheme has been reported, our investigations reveal that only using the η–η Hessian block is enough to provide robust and fast convergence in a memory-efficient manner. Our work provides new insight into the complex interplay between electrons and photons showing that, at the mean-field level, orbital rotations and electron-photon parameters are almost completely decoupled. In addition, our algorithms pave the way for developing computationally efficient post-mean-field methods. Specifically, coupled cluster and active space extensions would improve the description of electron-photon correlation while capturing the electron–electron correlation as well. Additionally, our improvements open new avenues for the development of multi-level methodologies to tackle the inclusion of solvent effects in QED environments. To this end, efficient screening of the photon-dressed two-electron integrals is necessary in order to reduce the computational scaling for large molecular systems. Future works will focus on response theory^38^ as well as the use of molecular orbitals to understand the cavity-induced modifications of molecular properties. Moreover, the generalization to a multi-mode Hamiltonian able to describe higher-order optical phenomena and the extension of the method to chiral cavities are currently in development.

## Supporting Information Available

The geometries used for the reported calculations as well as the results obtained for all the other molecules in Figure 1 can be found in the Detailed derivation of the Hessian equations and linear transformations (PDF)

## Accession Codes

The  program^36^ used to perform the calculations shown in this work is available from the corresponding author upon reasonable request.
