# Supplementary material for: Toward Polaritonic Molecular Orbitals for Large Molecular Systems
Source: J Chem Theory Comput. 2024 Sep 30;20(20):8911–20. doi: 10.1021/acs.jctc.4c00808 (PMC11500296; doi:10.1021/acs.jctc.4c00808)
Supplement: Supplementary file 1 — ct4c00808_si_001.pdf [file ct4c00808_si_001.pdf]

Supporting information to: Towards polaritonic molecular orbitals for large molecular systems

Yassir El Moutaoukal, Rosario R. Riso, Matteo Castagnola, and Henrik Koch*

Department of Chemistry, Norwegian University of Science and Technology, 7491 Trondheim, Norway

E-mail: henrik.koch@ntnu.no

S1 Wave function parametrization

The light-matter interaction between molecular systems and electromagnetic fields can be modeled with the single-mode Pauli-Fierz Hamiltonian:

$$\begin{aligned} H = &\sum_{pq} h_{pq} E_{pq} + \frac{1}{2}\sum_{pqrs} g_{pqrs} e_{pqrs} + \omega b^\dagger b \\ &+ \frac{\lambda^2}{2}(\mathbf{d}\cdot\boldsymbol{\epsilon})^2 - \lambda\sqrt{\frac{\omega}{2}}(\mathbf{d}\cdot\boldsymbol{\epsilon})(b^\dagger + b). \end{aligned} \tag{1}$$

In eq. (1), the bosonic operators $b^\dagger$ and b respectively create and annihilate a photonic mode of the cavity with frequency ω. The interaction is mediated through the bilinear term $\lambda\sqrt{\frac{\omega}{2}}(\mathbf{d}\cdot\boldsymbol{\epsilon})(b^\dagger + b)$ where $\boldsymbol{\epsilon}$ is the polarization vector of the field, λ is the coupling strength for a cavity with confinement volume V

$$\lambda \propto \sqrt{\frac{1}{V}}, \tag{2}$$

while $\mathbf{d}$ is the molecular dipole operator defined as

$$\mathbf{d} = \sum_{pq} \mathbf{d}_{pq} E_{pq} = \sum_{pq} \left(\mathbf{d}^e_{pq} + \frac{\mathbf{d}^{nuc}}{N_e} \delta_{pq} \right) E_{pq}, \tag{3}$$

with $\mathbf{d}^e$ being the electronic dipole and $\mathbf{d}^{nuc}$ the nuclear dipole of a system of N_e electrons. The electronic operators E_{pq} and e_{pqrs} are

$$\begin{aligned} E_{pq} &= \sum_{\sigma} a^{\dagger}_{p\sigma} a_{q\sigma} \\ e_{pqrs} &= E_{pq} E_{rs} - \delta_{rq} E_{ps}, \end{aligned} \tag{4}$$

where $a^{\dagger}_{p\sigma}$ and $a_{p\sigma}$ are the creation and annihilation operators for an electron in orbital p and spin σ. Finally, h_{pq} and g_{pqrs} are the one and two electron integrals associated to the electronic Hamiltonian in the Born-Oppenheimer approximation.

In the infinite coupling strength limit, the photonic part dominates and we thus employ the approximate Hamiltonian

$$H_{\infty} = \omega b^{\dagger} b - \lambda \sqrt{\frac{\omega}{2}} (\mathbf{d} \cdot \boldsymbol{\epsilon})(b^{\dagger} + b) + \frac{\lambda^2}{2} (\mathbf{d} \cdot \boldsymbol{\epsilon})^2. \tag{5}$$

The electronic part of the exact eigenfunctions are Slater determinants in the basis that diagonalizes the dipole operator $\mathbf{d}$, referred to as the dipole basis. In this basis, the infinite coupling Hamiltonian reads:

$$H_{\infty} = \omega b^{\dagger} b - \lambda \sqrt{\frac{\omega}{2}} \sum_{p} (\tilde{\mathbf{d}} \cdot \boldsymbol{\epsilon})_{pp} \tilde{E}_{pp} (b^{\dagger} + b) + \sum_{pq} \frac{\lambda^2}{2} (\tilde{\mathbf{d}} \cdot \boldsymbol{\epsilon})_{pp} (\tilde{\mathbf{d}} \cdot \boldsymbol{\epsilon})_{qq} \tilde{E}_{pp} \tilde{E}_{qq}. \tag{6}$$

This expression suggests to introduce a coherent state transformation to reabsorb the bilinear term and move quantum picture:

$$H_{\infty} = \omega b^{\dagger} b \tag{7}$$

The transformation able to do so by mixing the photonic and electronic degrees of freedom is

$$U_\infty = \exp\left(-\frac{\lambda}{\sqrt{2\omega}}\sum_p (\tilde{\mathbf{d}}\cdot\boldsymbol{\epsilon})_{pp}\tilde{E}_{pp}(b-b^\dagger)\right). \tag{8}$$

Now, we can relax the infinite coupling limit to a finite coupling strength recovering the electronic Hamiltonian

$$H = \tilde{H}_e + \omega\Big(b^\dagger - \frac{\lambda}{\sqrt{2\omega}}\sum_p (\tilde{\mathbf{d}}\cdot\boldsymbol{\epsilon})_{pp}\tilde{E}_{pp}\Big)\Big(b - \frac{\lambda}{\sqrt{2\omega}}\sum_p (\tilde{\mathbf{d}}\cdot\boldsymbol{\epsilon})_{pp}\tilde{E}_{pp}\Big) \tag{9}$$

and introducing in place of the dipole integrals the η-parameters: a novel set of orbital specific parameters

$$U_{\text{SC}} = \exp\left(-\frac{\lambda}{\sqrt{2\omega}}\sum_p \eta_p\tilde{E}_{pp}(b-b^\dagger)\right). \tag{10}$$

The SC-QED-HF parametrization of the wave function is then

$$|\psi_{\text{SC}}\rangle = \exp\left(-\frac{\lambda}{\sqrt{2\omega}}\sum_p \eta_p\tilde{E}_{pp}\left(b-b^\dagger\right)\right)e^{\kappa}\,|\text{HF}\rangle\otimes|0\rangle\,, \tag{11}$$

where

$$\kappa = \sum_{p>q}\kappa_{pq}(E_{pq}-E_{qp}) \tag{12}$$

is the anti-hermitian operator used for the SCF orbital rotations.

The SC-QED-HF wave function parametrization is constituted by two set of parameters, the η-parameters and the κ-parameters, and therefore both of them need to be optimized during the SCF procedure.

S2 SC-QED-HF Hessian matrix

The SC-parametrization in eq. (11) is composed by two different classes of parameters: the κ and the η parameters. For this reason the Hessian Matrix is be composed by 4 different blocks:

$$\mathbf{E}^{(2)} = \begin{pmatrix} \mathbf{E}^{\kappa\kappa} & \mathbf{E}^{\kappa\eta} \\ \mathbf{E}^{\eta\kappa} & \mathbf{E}^{\eta\eta} \end{pmatrix} = \begin{pmatrix} \frac{\partial^2 E}{\partial\kappa_{ai}\partial\kappa_{bj}} & \frac{\partial^2 E}{\partial\kappa_{ai}\partial\eta_r} \\ \frac{\partial^2 E}{\partial\eta_r\partial\kappa_{ai}} & \frac{\partial^2 E}{\partial\eta_r\partial\eta_s} \end{pmatrix}_{\boldsymbol{\kappa}=\mathbf{0}} . \tag{13}$$

where in top left we recognise the purely κ-κ block, in bottom right the η-η one and lastly the mixed parameters blocks in top right and bottom left. For each block, the derivatives can be taken at $\boldsymbol{\kappa} = \mathbf{0}$ if the Hessian is computed in the updated MO-basis. Instead, for the η-parameters, the derivatives are implicitly evaluated at the SCF converged values coming from the previous iteration.

In the next sections, each block contributing to the Hessian matrix $\mathbf{E}^{(2)}$ is explicitly derived for generic indices by calculating the proper commutators averaged on the reference wave function $|\text{HF}, 0\rangle$.

S2.1 The $\boldsymbol{\kappa}$-$\boldsymbol{\kappa}$ block

The Hessian elements for the κ-κ block are

$$\left(\frac{\partial^2 E}{\partial \kappa_{pq} \partial \kappa_{rs}}\right)_{\boldsymbol{\kappa}=\mathbf{0}} = (1 + P_{pq,rs}) \langle \mathrm{HF}, 0| \left[\left[U_{\mathrm{SC}}^{\dagger} H U_{\mathrm{SC}}, E_{pq}\right], E_{rs}^{-}\right] |\mathrm{HF}, 0\rangle \tag{14}$$

where $P_{mn,lo}$ permutes the m and n indices with the l and o ones.

By applying the SC-transformation (10) to the Hamiltonian (9), we obtain the SC-transformed dipole Hamiltonian, which reads

$$\begin{aligned} H_{\mathrm{SC}} &= U_{\mathrm{SC}}^{\dagger} H U_{\mathrm{SC}} \\ &= H_e^{\mathrm{SC}} + \omega\Big(b^{\dagger} - \frac{\lambda}{\sqrt{2\omega}} \sum_p ((\tilde{\mathbf{d}} \cdot \boldsymbol{\epsilon})_{pp} - \eta_p) \tilde{E}_{pp}\Big)\Big(b - \frac{\lambda}{\sqrt{2\omega}} \sum_p ((\tilde{\mathbf{d}} \cdot \boldsymbol{\epsilon})_{pp} - \eta_p) \tilde{E}_{pp}\Big) \end{aligned} \tag{15}$$

where the transformed electronic Hamiltonian is defined as:

$$\begin{aligned} H_e^{\mathrm{SC}} = &\sum_{pq} \tilde{h}_{pq} \tilde{E}_{pq} \exp\left(\frac{\lambda}{\sqrt{2\omega}} (\eta_p - \eta_q)(b - b^{\dagger})\right) \\ &+ \frac{1}{2} \sum_{pqrs} \tilde{g}_{pqrs} \tilde{e}_{pqrs} \exp\left(\frac{\lambda}{\sqrt{2\omega}} (\eta_p + \eta_r - \eta_q - \eta_s)(b - b^{\dagger})\right). \end{aligned} \tag{16}$$

Notice that in the electronic Hessian of Eq. 14, the operators E_{mn} and E_{lo}^{-} act exclusively on the electronic degrees of freedom. Therefore, the average over the photon vacuum $|0\rangle$ involves only the Hamiltonian H_{SC}, giving an effective electronic operator. In particular, there are contributions only from the (transformed) electronic Hamiltonian H_e^{SC} and the dipole self-energy.

Taking then the vacuum average on the SC-tranformed Hamiltonian (15) we obtain

$$\begin{aligned}\langle H_{\mathrm{SC}}\rangle_0 &= \sum_{pq}\tilde{h}_{pq}\exp\biggl(-\frac{\lambda^2}{4\omega}(\eta_p-\eta_q)^2\biggr)\tilde{E}_{pq}\\ &+\frac{1}{2}\sum_{pqrs}\tilde{g}_{pqrs}\exp\biggl(-\frac{\lambda^2}{4\omega}(\eta_p+\eta_r-\eta_q-\eta_s)^2\biggr)\tilde{e}_{pqrs}\\ &+\frac{\lambda^2}{2}\sum_{pq}((\tilde{\mathbf{d}}\cdot\boldsymbol{\epsilon})_{pp}-\eta_p)((\tilde{\mathbf{d}}\cdot\boldsymbol{\epsilon})_{qq}-\eta_q)(\tilde{e}_{ppqq}+\tilde{E}_{qp}\delta_{pq})\end{aligned} \tag{17}$$

It is convenient to rewrite the above equation in an electronic Hamiltonian fashion

$$\langle H_{\mathrm{SC}}\rangle_0 = \sum_{pq}\tilde{h}^b_{pq}\tilde{E}_{pq}+\frac{1}{2}\sum_{pqrs}\tilde{g}^b_{pqrs}\tilde{e}_{pqrs} \tag{18}$$

where we have redefined the one and two electrons integrals:

$$\tilde{h}^b_{pq} = \tilde{h}_{pq}\exp\biggl(-\frac{\lambda^2}{4\omega}(\eta_p-\eta_q)^2\biggr)+\frac{\lambda^2}{2}((\tilde{\mathbf{d}}\cdot\boldsymbol{\epsilon})_{pp}-\eta_p)^2\delta_{pq}, \tag{19}$$

$$\tilde{g}^b_{pqrs} = \tilde{g}_{pqrs}\exp\biggl(-\frac{\lambda^2}{4\omega}(\eta_p+\eta_r-\eta_q-\eta_s)^2\biggr)+\lambda^2((\tilde{\mathbf{d}}\cdot\boldsymbol{\epsilon})_{pp}-\eta_p)((\tilde{\mathbf{d}}\cdot\boldsymbol{\epsilon})_{rr}-\eta_r)\delta_{pq}\delta_{rs}. \tag{20}$$

The κ-κ block elements of the SC-QED-HF Hessian read

$$\left(\frac{\partial^2 E}{\partial\kappa_{pq}\partial\kappa_{rs}}\right)_{\boldsymbol{\kappa}=\mathbf{0}} = (1+P_{pq,rs})\,\langle\mathrm{HF}|\,\bigl[\bigl[\,\langle H_{\mathrm{SC}}\rangle_0\,,E_{pq}\bigr],E^-_{rs}\bigr]\,|\mathrm{HF}\rangle\,, \tag{21}$$

where the vacuum averaged SC-transformed Hamiltonian $\langle H_{\mathrm{SC}}\rangle_0$ involved in the commutators assumes the shape of a purely electronic Hamiltonian with redefined integrals. This electron-like redefinition is also useful because, once we have explicitly derived the equations for the Hessian elements, we can compare them with the ones coming from the Hartree Fock theory:

$$E^{(2)}_{HF,aibj} = 4\Bigl[\delta_{ab}\delta_{ij}\,(\epsilon_a-\epsilon_i)+4g_{aibj}-g_{abji}-g_{ajbi}\Bigr]. \tag{22}$$

The Hessian elements in eq. (21) can be rewritten as

$$\left(\frac{\partial^2 E}{\partial \kappa_{pq} \partial \kappa_{rs}}\right)_{\boldsymbol{\kappa}=\mathbf{0}} = (1 + P_{pq,rs})(G_{pqrs} - G_{pqsr}) \tag{23}$$

where

$$G_{pqrs} = \langle \mathrm{HF} | \left[\left[\langle H_{\mathrm{SC}} \rangle_0 , E_{pq} \right], E_{rs} \right] | \mathrm{HF} \rangle \, . \tag{24}$$

The first common commutator for both G_{pqrs} and G_{pqrs} is

$$\begin{aligned} \left[\langle H_{\mathrm{SC}} \rangle_0 , E_{pq} \right] = & \sum_t \Big(\sum_{rs} U_{rt} \tilde{h}^b_{rs} U^*_{sp} \Big) E_{tq} - \sum_u \Big(\sum_{rs} U_{rq} \tilde{h}^b_{rs} U^*_{su} \Big) E_{pu} \\ & + \sum_{txy} \Big(\sum_{rsvz} U_{rt} U_{vx} \tilde{g}^b_{rsvz} U^*_{sp} U^*_{zy} \Big) e_{tqxy} - \sum_{uxy} \Big(\sum_{rsvz} U_{rq} U_{vy} \tilde{g}^b_{rsvz} U^*_{su} U^*_{zx} \Big) e_{puxy} \end{aligned} \tag{25}$$

where $\mathbf{U}$ is the canonical to dipole basis transformation.

Transforming the $\tilde{h}^b_{pq}$ and $\tilde{g}^b_{pqrs}$ integrals back to the canonical basis and calculating the second commutator with E_{rs} in G_{pqrs}, we obtain

$$\begin{aligned} G_{pqrs} = \langle \mathrm{HF} | \Big(& \sum_t h^b_{tp} (E_{ts} \delta_{rq} - E_{rq} \delta_{ts}) - \sum_u h^b_{qu} (E_{ps} \delta_{ru} - E_{ru} \delta_{ps}) \\ & + \sum_{tvz} g^b_{tpvz} (e_{tsvz} \delta_{rq} + e_{tqvs} \delta_{rz} - e_{rqvz} \delta_{ts} - e_{tqrz} \delta_{vs}) \\ & - \sum_{uvz} g^b_{quvz} (e_{psvz} \delta_{ru} + e_{puvs} \delta_{rz} - e_{ruvz} \delta_{ps} - e_{purz} \delta_{vs}) \Big) | \mathrm{HF} \rangle \, . \end{aligned} \tag{26}$$

Now we can average the electronic operators on the Hartree Fock determinant and obtain the density matrices elements which, in the canonical basis, read

$$D_{pq} = \langle \mathrm{HF}| \, E_{pq} \, |\mathrm{HF}\rangle = 2\delta_{pq}\delta_{p,occ}, \tag{27}$$

$$d_{pqrs} = \langle \mathrm{HF}| \, e_{pqrs} \, |\mathrm{HF}\rangle = 4\delta_{pq}\delta_{rs}\delta_{p,occ}\delta_{r,occ} - 2\delta_{ps}\delta_{qr}\delta_{p,occ}\delta_{q,occ}. \tag{28}$$

Then, G_{pqrs} becomes

$$\begin{aligned} G_{pqrs} = {} & 2\Big(\delta_{rq}(h^b_{sp} + \sum_j^{occ}(2g^b_{sjj} - g^b_{sjjp})) - \delta_{ps}(h^b_{qr} + \sum_j^{occ}(2g^b_{qrjj} - g^b_{qjjr}))\Big)(\delta_{s,occ} - \delta_{r,occ}) \\ & + (\delta_{q,occ} - \delta_{p,occ})(\delta_{s,occ} - \delta_{r,occ})\Big(g^b_{pqrs} - \frac{1}{2}g^b_{psrq}\Big), \end{aligned} \tag{29}$$

while G_{pqsr} is easily obtained from the previous equation by simply swapping the r and s indices. We can recognise from the above eq. (29) the presence of the Fock matrix elements for our electronic-like Hamiltonian (18), transformed back to the canonical basis

$$F_{pq} = h^b_{pq} + \sum_j^{occ}(2g^b_{pqjj} - g^b_{pjjq}) \tag{30}$$

which is related to the non-redundant Hartree-Fock gradient

$$f^{\kappa}_{pq} = \left(\frac{\partial E}{\partial \kappa_{pq}}\right)_{\boldsymbol{\kappa}=\mathbf{0}} = \langle \mathrm{HF}| \left[\langle H_{\mathrm{SC}}\rangle_0 \, , E^-_{pq}\right] |\mathrm{HF}\rangle = 2(F_{pq} - F_{qp})(\delta_{q,occ} - \delta_{p,occ}), \tag{31}$$

where

$$F_{pq} = \sum_{rs} V_{pr}\tilde{F}_{rs}V_{qs}. \tag{32}$$

and

$$\tilde{F}_{pq} = \frac{\partial E}{\partial \tilde{D}_{pq}} = \tilde{h}_{pq} \exp\left(-\frac{\lambda^2}{4\omega}(\eta_p - \eta_q)^2\right) + \frac{\lambda^2}{2}\delta_{pq}((\tilde{\mathbf{d}}\cdot\boldsymbol{\epsilon})_{pp} - \eta_p)^2$$
$$+ \frac{1}{2}\sum_{rs}(2\tilde{g}_{pqrs} - \tilde{g}_{psrq})\tilde{D}_{rs}\exp\left(-\frac{\lambda^2}{4\omega}(\eta_p + \eta_r - \eta_q - \eta_s)^2\right)$$
$$- \frac{\lambda^2}{2}\tilde{D}_{qp}((\tilde{\mathbf{d}}\cdot\boldsymbol{\epsilon})_{pp} - \eta_p)((\tilde{\mathbf{d}}\cdot\boldsymbol{\epsilon})_{qq} - \eta_q)$$
$$+ \lambda^2\delta_{pq}((\tilde{\mathbf{d}}\cdot\boldsymbol{\epsilon})_{pp} - \eta_p)\sum_{r}\tilde{D}_{rr}((\tilde{\mathbf{d}}\cdot\boldsymbol{\epsilon})_{rr} - \eta_r). \quad (33)$$

By introducing the Fock matrix elements in G_{pqrs} and G_{pqsr} and substituting the last two in eq. (23), we obtain

$$\left(\frac{\partial^2 E}{\partial\kappa_{pq}\partial\kappa_{rs}}\right)_{\boldsymbol{\kappa}=\mathbf{0}} = (1 + P_{pq,rs})\Big(2(\delta_{qr}F_{ps} - \delta_{ps}F_{qr} + \delta_{qs}F_{pr} - \delta_{pr}F_{qs})(\delta_{s,occ} - \delta_{r,occ})$$
$$+ \Big[4(g^b_{pqrs} + g^b_{pqsr}) - 2(g^b_{psrq} + g^b_{prsq})\Big](\delta_{q,occ} - \delta_{p,occ})(\delta_{s,occ} - \delta_{r,occ})\Big). \quad (34)$$

Finally, by the application of the permutator $P_{pq,rs}$ we obtain

$$\left(\frac{\partial^2 E}{\partial\kappa_{pq}\partial\kappa_{rs}}\right)_{\boldsymbol{\kappa}=\mathbf{0}} = \Big[(\delta_{s,occ} - \delta_{r,occ}) - (\delta_{q,occ} - \delta_{p,occ})\Big]2(\delta_{qr}F_{ps} - \delta_{ps}F_{qr})$$
$$+ \Big[(\delta_{s,occ} - \delta_{r,occ}) + (\delta_{q,occ} - \delta_{p,occ})\Big]2(\delta_{qs}F_{pr} - \delta_{pr}F_{qs})$$
$$+ (\delta_{q,occ} - \delta_{p,occ})(\delta_{s,occ} - \delta_{r,occ})\Big[8(\mathring{g}_{pqrs} + \mathring{g}_{pqsr}) - 4(\mathring{g}_{psrq} + \mathring{g}_{prsq})\Big]. \quad (35)$$

From this last equation we can recognise that the only non-redundant derivatives are those arising from two κs both respectively constituted by one occupied and one virtual indices. Considering that in eq. (12) we have only κ_{pq} parameters where $p > q$, the non-vanishing elements of the κ-κ block of the Hessian matrix are

$$\left(\frac{\partial^2 E}{\partial\kappa_{ai}\partial\kappa_{bj}}\right) = 4\Big[F_{ab}\delta_{ij} - F_{ij}\delta_{ab} + 2(g^b_{aibj} + g^b_{aijb}) - g^b_{abji} - g^b_{ajbi}\Big] \quad (36)$$

which can be further simplified recognising that the Fock matrix is diagonal in the canonical basis:

$$\left(\frac{\partial^2 E}{\partial\kappa_{ai}\partial\kappa_{bj}}\right)_{\boldsymbol{\kappa}=\mathbf{0}} = 4\Big[\delta_{ab}\delta_{ij}(\epsilon_a - \epsilon_i) + 2(g^b_{aibj} + g^b_{aijb}) - g^b_{abji} - \mathring{g}_{ajbi}\Big]. \tag{37}$$

Equation (37) represents the κ-κ block elements of the Hessian and by comparing it with the Hartree Fock Hessian in eq. (22) we can observe that the g^b_{aibj} and g^b_{aijb} terms don't sum up giving a factor of 4 like in the Hartree Fock case. This happens because, according to eq. (20), the redefined two electrons integrals pass from an eigth-fold symmetry to a fourth-fold one: the symmetry of interchanging two real orbitals belonging to the same electron and leaving unchanged the ones for the other electron is lost. Only interchanging both the couples is allowed due to the presence of the cavity Gaussian factors.

Lastly, we can observe how the κ-κ block of the Hessian reduces to the Hartree Fock one at zero coupling restoring also the lost symmetry of the integrals:

$$g^b_{pqrs} \xrightarrow{\lambda=0} g_{pqrs} \ . \tag{38}$$

S2.2 The $\boldsymbol{\eta}$-$\boldsymbol{\eta}$ block

The Hessian elements for the η-η block are

$$\left(\frac{\partial^2 E}{\partial\eta_r\partial\eta_s}\right)_{\boldsymbol{\kappa}=\mathbf{0}} = \frac{\lambda^2}{2\omega}\langle \text{HF}, 0|\left[\tilde{E}_{ss}(b-b^\dagger), \left[\tilde{E}_{rr}(b-b^\dagger), H_{\text{SC}}\right]\right]|\text{HF}, 0\rangle \tag{39}$$

where H_{SC} is defined in eq. (15).

Unlike for the κ-κ block of the Hessian, we cannot average H_{SC} on the photonic vacuum and work with a simpler electronic-like Hamiltonian with redefined integrals. This happens because in the commutators in eq. (39) we have several photonic operators (b and $b^\dagger$) so that the effect of the vacuum average is not trivial to evaluate.

However, we can notice which terms of H_{SC} have a non-zero contribution by expanding the commutators to separate the photonic and electronic operators. Using

$$\left[AB, C\right] = A\left[B, C\right] + \left[A, C\right]B, \tag{40}$$

the first commutator can be written as

$$\left[\tilde{E}_{rr}(b-b^\dagger), \tilde{H}_{SC}\right] = \tilde{E}_{rr}\left[(b-b^\dagger), \tilde{H}_{SC}\right] + \left[\tilde{E}_{rr}, \tilde{H}_{SC}\right](b-b^\dagger) \tag{41}$$

and by substituting it into the second commutator we obtain:

$$\left[\tilde{E}_{ss}(b-b^\dagger), \tilde{E}_{rr}\left[(b-b^\dagger), \tilde{H}_{SC}\right] + \left[\tilde{E}_{rr}, \tilde{H}_{SC}\right](b-b^\dagger)\right]. \tag{42}$$

Using again eq. (40) we finally end up with the sum of the following 4 terms:

$$\tilde{E}_{ss}\tilde{E}_{rr}\big[(b-b^\dagger),\big[(b-b^\dagger),\tilde{H}_{SC}\big]\big], \tag{43}$$

$$\tilde{E}_{ss}\big[(b-b^\dagger),\big[\tilde{E}_{rr},\tilde{H}_{SC}\big]\big](b-b^\dagger), \tag{44}$$

$$\tilde{E}_{rr}\big[\tilde{E}_{rr},\big[(b-b^\dagger),\tilde{H}_{SC}\big]\big](b-b^\dagger), \tag{45}$$

$$\big[\tilde{E}_{ss},\big[\tilde{E}_{rr},\tilde{H}_{SC}\big]\big]\left(b-b^\dagger\right)(b-b^\dagger). \tag{46}$$

Now, it's easy to verify that, once the vacuum average is taken, the addends in eqs. (44) and (45) vanish while for those in eqs. (43) and (46) only the purely photonic $\omega b^\dagger b$ and SC-electronic H_e^{SC} in eq. (16) respectively contribute.

Equation 39 for the η-η block elements of the Hessian reduces to

$$\begin{aligned}\left(\frac{\partial^2 E}{\partial\eta_r\partial\eta_s}\right)_{\boldsymbol{\kappa}=\mathbf{0}} = \frac{\lambda^2}{2\omega}\Big(&\langle\mathrm{HF}|\,\tilde{E}_{ss}\tilde{E}_{rr}\,|\mathrm{HF}\rangle\,\langle 0|\,\big[(b-b^\dagger),\big[(b-b^\dagger),\omega b^\dagger b\big]\big]\,|0\rangle \\ &+\langle\mathrm{HF},0|\,\big[\tilde{E}_{ss},\big[\tilde{E}_{rr},H_e^{\mathrm{SC}}\big]\big](b-b^\dagger)^2\,|\mathrm{HF},0\rangle\Big).\end{aligned} \tag{47}$$

The first term in the parenthesis is simply

$$\langle\mathrm{HF}|\,\tilde{E}_{ss}\tilde{E}_{rr}\,|\mathrm{HF}\rangle\,\langle 0|\,\big[(b-b^\dagger),\big[(b-b^\dagger),\omega b^\dagger b\big]\big]\,|0\rangle = 2\omega(\tilde{D}_{rr}\delta_{rs}+\tilde{d}_{rrss}) \tag{48}$$

where $\tilde{D}_{pq}$ and $\tilde{d}_{pqrs}$ are the one and two body density matrices in the dipole basis.

For the second more complex term we have:

$$
\begin{aligned}
&\langle \mathrm{HF},0|\left[\tilde{E}_{ss},\left[\tilde{E}_{rr},H_e^{\mathrm{SC}}\right]\right](b-b^\dagger)^2\,|\mathrm{HF},0\rangle = \\
&=\sum_{pq}\tilde{h}_{pq}\,\langle \mathrm{HF}|\left[\tilde{E}_{ss},\left[\tilde{E}_{rr},\tilde{E}_{pq}\right]\right]|\mathrm{HF}\rangle\,\langle 0|\exp\!\left(\frac{\lambda}{\sqrt{2\omega}}\,(\eta_p-\eta_q)\,\big(b-b^\dagger\big)\right)\big(b-b^\dagger\big)^2\,|0\rangle \\
&+\frac{1}{2}\sum_{pqtu}\tilde{g}_{pqtu}\,\langle \mathrm{HF}|\left[\tilde{E}_{ss},\left[\tilde{E}_{rr},\tilde{e}_{pqtu}\right]\right]|\mathrm{HF}\rangle\,\langle 0|\exp\!\left(\frac{\lambda}{\sqrt{2\omega}}\,(\eta_p+\eta_r-\eta_q-\eta_s)\,\big(b-b^\dagger\big)\right)\big(b-b^\dagger\big)^2\,|0\rangle\,.
\end{aligned}
\tag{49}
$$

Using the following relation

$$
\langle 0|\exp\!\big(\alpha(b-b^\dagger)\big)(b-b^\dagger)^2\,|0\rangle=(\alpha^2-1)\exp\!\left(-\frac{\alpha^2}{2}\right)
\tag{50}
$$

and expliciting all the commutators in eq. (49), we end up with

$$
\begin{aligned}
&\langle \mathrm{HF},0|\left[\tilde{E}_{ss},\left[\tilde{E}_{rr},H_{el}^{\mathrm{SC}}\right]\right](b-b^\dagger)^2\,|\mathrm{HF},0\rangle = \\
&=2\Bigg(\delta_{rs}\sum_{q}\tilde{h}^a_{rq}\tilde{D}_{rq}\Big(\frac{\lambda^2}{2\omega}(\eta_r-\eta_q)^2-1\Big)-\tilde{h}^a_{rs}\tilde{D}_{rs}\Big(\frac{\lambda^2}{2\omega}(\eta_r-\eta_s)^2-1\Big) \\
&\quad+\delta_{rs}\sum_{pqt}\tilde{g}^a_{rpqt}\tilde{d}_{rpqt}\Big(\frac{\lambda^2}{2\omega}(\eta_r+\eta_q-\eta_p-\eta_t)^2-1\Big) \\
&\quad-\sum_{qt}\tilde{g}^a_{rsqt}\tilde{d}_{rsqt}\Big(\frac{\lambda^2}{2\omega}(\eta_r+\eta_q-\eta_s-\eta_t)^2-1\Big) \\
&\quad+\sum_{qt}\tilde{g}^a_{rqst}\tilde{d}_{rqst}\Big(\frac{\lambda^2}{2\omega}(\eta_r+\eta_s-\eta_q-\eta_t)^2-1\Big) \\
&\quad-\sum_{qt}\tilde{g}^a_{rqts}\tilde{d}_{rqts}\Big(\frac{\lambda^2}{2\omega}(\eta_r+\eta_t-\eta_q-\eta_s^2-1)\Big)\Bigg)
\end{aligned}
\tag{51}
$$

where we introduced the Gaussian scaled integrals $\bar{\tilde{h}}_{pq}$ and $\bar{\tilde{g}}_{pqrs}$:

$$
\tilde{h}^a_{pq}=\tilde{h}_{pq}\exp\!\left(-\frac{\lambda^2}{4\omega}(\eta_p-\eta_q)^2\right),
\tag{52}
$$

$$
\tilde{g}^a_{pqrs}=\tilde{g}_{pqrs}\exp\!\left(-\frac{\lambda^2}{4\omega}(\eta_p+\eta_r-\eta_q-\eta_s)^2\right).
\tag{53}
$$

Finally, by substituting the terms in eqs. (48) and (51) into eq. (47) we end up with

$$\begin{aligned}\left(\frac{\partial^2 E}{\partial\eta_r\partial\eta_s}\right)_{\boldsymbol{\kappa}=\mathbf{0}} &= \delta_{rs}\Bigg(\frac{\lambda^2}{\omega}\sum_q \tilde{h}^a_{rq}\tilde{D}_{rq}\Big(\frac{\lambda^2}{2\omega}(\eta_r-\eta_q)^2-1\Big)+\lambda^2\tilde{D}_{rr} \\ &\qquad + \frac{\lambda^2}{\omega}\sum_{pqt}\tilde{g}^a_{rpqt}\tilde{d}_{rpqt}\Big(\frac{\lambda^2}{2\omega}(\eta_r+\eta_q-\eta_p-\eta_t)^2-1\Big)\Bigg) \\ &\quad - \frac{\lambda^2}{\omega}\sum_{qt}\tilde{g}^a_{rsqt}\tilde{d}_{rsqt}\Big(\frac{\lambda^2}{2\omega}(\eta_r+\eta_q-\eta_s-\eta_t)^2-1\Big) \\ &\quad + \frac{\lambda^2}{\omega}\sum_{qt}\tilde{g}^a_{rqst}\tilde{d}_{rqst}\Big(\frac{\lambda^2}{2\omega}(\eta_r+\eta_s-\eta_q-\eta_t)^2-1\Big) \\ &\quad - \frac{\lambda^2}{\omega}\sum_{qt}\tilde{g}^a_{rqts}\tilde{d}_{rqts}\Big(\frac{\lambda^2}{2\omega}(\eta_r+\eta_t-\eta_q-\eta_s)^2-1\Big) \\ &\quad + \lambda^2\tilde{d}_{rrss} - \frac{\lambda^2}{\omega}\tilde{h}^a_{rs}\tilde{D}_{rs}\Big(\frac{\lambda^2}{2\omega}(\eta_r-\eta_s)^2-1\Big).\end{aligned} \tag{54}$$

for the η-η block elements of the SC-Hessian matrix.

Note that this block was derived in the dipole basis contrary to the κ-κ block which was derived in the canonical one. Note also that this result is in totally agreement with the result we would have more tediously obtained by directly deriving from the η gradient equation written in the dipole basis

$$\begin{aligned}\tilde{f}^\eta_r = \frac{\partial E}{\partial\eta_r} &= \frac{\lambda^2}{\omega}\sum_q \tilde{h}^a_{rq}\tilde{D}_{rq}(\eta_q-\eta_r) - \lambda^2\tilde{D}_{rr}((\tilde{\mathbf{d}}\cdot\boldsymbol{\epsilon})_{rr}-\eta_r) \\ &\quad + \frac{\lambda^2}{\omega}\sum_{pqt}\tilde{g}^a_{rpqt}\tilde{d}_{rpqt}(\eta_p+\eta_t-\eta_r-\eta_q) \\ &\quad - \lambda^2\sum_q \tilde{d}_{qqrr}((\tilde{\mathbf{d}}\cdot\boldsymbol{\epsilon})_{qq}-\eta_q).\end{aligned} \tag{55}$$

To conclude, we point out that no redundancies are obtained for this block of the Hessian contrary to the κ-κ block derivide in the canonical basis.

S2.3 The $\boldsymbol{\kappa}$-$\boldsymbol{\eta}$ and $\boldsymbol{\eta}$-$\boldsymbol{\kappa}$ blocks

The Hessian elements for the κ-η and η-κ blocks are

$$\left(\frac{\partial^2 E}{\partial \kappa_{pq} \partial \eta_r}\right)_{\boldsymbol{\kappa}=\mathbf{0}} = \left(\frac{\partial^2 E}{\partial \eta_r \partial \kappa_{pq}}\right)_{\boldsymbol{\kappa}=\mathbf{0}} = \lambda \sqrt{\frac{2}{\omega}} \langle \mathrm{HF}, 0| \left[\left[\tilde{E}_{rr}(b - b^\dagger), H_{\mathrm{SC}}\right], E_{pq}\right] |\mathrm{HF}, 0\rangle \tag{56}$$

where H_{SC} is defined in eq. (15).

We point out that these blocks turn to be naturally identical and in the Hessian matrix they would simply be one the transpose of the other and vice versa.

Like for the η-η block case, we cannot directly take the vacuum average on H_{SC} and we should split the first commutator in order to separate the photonic and the electronic operators. Then, using eq. (41) and making observations on the resulting commutators looking forward on what would be the effect of taking the vacuum average, we obtain

$$\left(\frac{\partial^2 E}{\partial \kappa_{pq} \partial \eta_r}\right)_{\boldsymbol{\kappa}=\mathbf{0}} = \left(\frac{\partial^2 E}{\partial \eta_r \partial \kappa_{pq}}\right)_{\boldsymbol{\kappa}=\mathbf{0}} = \sum_{tu} U^*_{tp} \tilde{H}_{r,tu} U_{uq} \tag{57}$$

where $\tilde{H}_{r,tu}$ has the following definition

$$\begin{aligned} \tilde{H}_{r,tu} = {} & 2\lambda^2 \sum_p ((\tilde{\mathbf{d}} \cdot \boldsymbol{\epsilon})_{pp} - \eta_p) \langle \mathrm{HF}| \left[\tilde{E}_{tu}, \tilde{e}_{rrpp} + \tilde{E}_{pr}\delta_{rp}\right] |\mathrm{HF}\rangle \\ & + \lambda \sqrt{\frac{2}{\omega}} \langle \mathrm{HF}, 0| \left[\left[\tilde{E}_{rr}, H_e^{SC}\right](b - b^\dagger), \tilde{E}_{tu}\right] |\mathrm{HF}, 0\rangle \,, \end{aligned} \tag{58}$$

$\mathbf{U}$ is the canonical to dipole basis transformation and H_e^{SC} is defined in eq. (16).

By expliciting the commutator in the first term we obtain

$$
\begin{aligned}
&2\lambda^2 \sum_p ((\mathbf{d}\cdot\boldsymbol{\epsilon})_{pp} - \eta_p) \langle \mathrm{HF}| \left[\tilde{E}_{tu}, \tilde{e}_{rrpp} + \tilde{E}_{pr}\delta_{rp}\right] |\mathrm{HF}\rangle = \\
&= (1-P_{t,u}) 2\lambda^2 \Big(\delta_{ru}\big(\tilde{D}_{tr}((\tilde{\mathbf{d}}\cdot\boldsymbol{\epsilon})_{rr} - \eta_r) + \sum_p ((\tilde{\mathbf{d}}\cdot\boldsymbol{\epsilon})_{pp} - \eta_p)\tilde{d}_{trpp}\big) + \tilde{d}_{rrtu}((\tilde{\mathbf{d}}\cdot\boldsymbol{\epsilon})_{uu} - \eta_u)\Big)
\end{aligned}
\tag{59}
$$

where $P_{t,u}$ swaps the t and u indices.

For the second more complex term, by expliciting H_e^{SC} given eq. (16), we have

$$
\begin{aligned}
&\lambda\sqrt{\frac{2}{\omega}} \langle \mathrm{HF}, 0| \left[\left[\tilde{E}_{rr}, H_e^{\mathrm{SC}}\right](b-b^\dagger), \tilde{E}_{tu}\right] |\mathrm{HF}, 0\rangle = \\
&= \lambda\sqrt{\frac{2}{\omega}} \sum_{pq} \tilde{h}_{pq} \langle \mathrm{HF}| \left[\left[\tilde{E}_{rr}, \tilde{E}_{pq}\right], \tilde{E}_{tu}\right] |\mathrm{HF}\rangle \langle 0| \exp\left(\frac{\lambda}{\sqrt{2\omega}}(\eta_p - \eta_q)(b-b^\dagger)\right) b^\dagger |0\rangle \\
&+ \frac{\lambda}{\sqrt{2\omega}} \sum_{pqvz} \tilde{g}_{pqvz} \langle \mathrm{HF}| \left[\left[\tilde{E}_{rr}, \tilde{e}_{pqvz}\right], \tilde{E}_{tu}\right] |\mathrm{HF}\rangle \langle 0| \exp\left(\frac{\lambda}{\sqrt{2\omega}}(\eta_p + \eta_v - \eta_q - \eta_z)(b-b^\dagger)\right) b^\dagger |0\rangle \,.
\end{aligned}
\tag{60}
$$

Using the following relation

$$
\langle 0| \exp\big(\alpha(b-b^\dagger)\big) b^\dagger |0\rangle = \alpha \exp\left(-\frac{\alpha^2}{2}\right) \tag{61}
$$

and expanding all the commutators, we obtain

$$
\begin{aligned}
&\lambda\sqrt{\frac{2}{\omega}} \langle \mathrm{HF}, 0| \left[\left[\tilde{E}_{rr}, H_e^{\mathrm{SC}}\right](b-b^\dagger), \tilde{E}_{tu}\right] |\mathrm{HF}, 0\rangle = \\
&= (1-P_{t,u}) \frac{\lambda^2}{\omega} \Bigg(\delta_{ru}\Big(\sum_q \tilde{h}^c_{qr}\tilde{D}_{qt} + \sum_{qvz} \tilde{g}^c_{qrvz}\tilde{d}_{qtvz}\Big) + \tilde{h}^c_{rt}\tilde{D}_{ru} \\
&\qquad + \sum_{vz} \tilde{g}^c_{rtvz}\tilde{d}_{ruvz} - \sum_{qz} \tilde{g}^c_{rquz}\tilde{d}_{rqtz} + \sum_{qv} \tilde{g}^c_{rqvt}\tilde{d}_{rqvu}\Bigg)
\end{aligned}
\tag{62}
$$

where the $\tilde{h}^c_{pq}$ and $\tilde{g}^c_{pqrs}$ integrals are defined as follows:

$$\tilde{h}^c_{pq} = \tilde{h}_{pq} \exp\left(-\frac{\lambda^2}{4\omega}(\eta_p - \eta_q)^2\right)(\eta_q - \eta_p) \ , \tag{63}$$

$$\tilde{g}^c_{pqrs} = \tilde{g}_{pqrs} \exp\left(-\frac{\lambda^2}{4\omega}(\eta_p + \eta_r - \eta_q - \eta_s)^2\right)(\eta_q + \eta_s - \eta_p - \eta_r) \ . \tag{64}$$

Finally, by adding together the terms in eqs. (59) and (62) we end up with the following formula for the κ-η and η-κ blocks elements of the SC-Hessian:

$$\left(\frac{\partial^2 E}{\partial \kappa_{pq} \partial \eta_r}\right)_{\boldsymbol{\kappa}=\mathbf{0}} = \left(\frac{\partial^2 E}{\partial \eta_r \partial \kappa_{pq}}\right)_{\boldsymbol{\kappa}=\mathbf{0}} = \sum_{tu} U^*_{tp} \tilde{H}_{r,tu} U_{uq} \tag{65}$$

where $\tilde{H}_{r,tu}$ is defined as:

$$\begin{aligned}\tilde{H}_{r,tu} = &(1 - P_{t,u}) \Bigg(\frac{\lambda^2}{\omega} \Big(\delta_{ru} \big(\sum_q \tilde{h}^c_{qr} \tilde{D}_{qt} + \sum_{qvz} \tilde{g}^c_{qrvz} \tilde{d}_{qtvz} \big) + \tilde{h}^c_{rt} \tilde{D}_{ru} \\ &+ \sum_{vz} \tilde{g}^c_{rtvz} \tilde{d}_{ruvz} - \sum_{qz} \tilde{g}^c_{rquz} \tilde{d}_{rqtz} + \sum_{qv} \tilde{g}^c_{rqvt} \tilde{d}_{rqvu} \Big) \\ &+ 2\lambda^2 \Big(\delta_{ru} \big(\tilde{D}_{tr} ((\tilde{\mathbf{d}} \cdot \boldsymbol{\epsilon})_{rr} - \eta_r) + \sum_p ((\tilde{\mathbf{d}} \cdot \boldsymbol{\epsilon})_{pp} - \eta_p) \tilde{d}_{trpp} \big) + \tilde{d}_{rrtu} ((\tilde{\mathbf{d}} \cdot \boldsymbol{\epsilon})_{uu} - \eta_u) \Big) \Bigg) .\end{aligned} \tag{66}$$

We point out that no apparent redundancies emerged from the derivation of the mixed parameter blocks because we derived $\tilde{H}_{r,tu}$ in the dipole basis without explicitly transforming back the t and u indices to the canonical one. However, by doing so we would obtain the usual occupied-occupied and virtual-virtual redundancies brought by the κ-parameters:

$$\left(\frac{\partial^2 E}{\partial \kappa_{ai} \partial \eta_r}\right)_{\boldsymbol{\kappa}=\mathbf{0}} = \left(\frac{\partial^2 E}{\partial \eta_r \partial \kappa_{ai}}\right)_{\boldsymbol{\kappa}=\mathbf{0}} = \sum_{tu} U^*_{ta} \tilde{H}_{r,tu} U_{ui} \ . \tag{67}$$

S3 Trust region Newton-Raphson algorithm

If we consider an Hamiltonian H and a parametrized wave function $|\psi(\mathbf{z})\rangle$ where $\mathbf{z}$ is the parameters vector, the energy hypersurface reads

$$E(\mathbf{z}) = \langle\psi(\mathbf{z})| H |\psi(\mathbf{z})\rangle . \tag{68}$$

By approximating the above equation to a quadratic model we obtain

$$E(\mathbf{z}+\boldsymbol{\delta}) \approx Q(\boldsymbol{\delta}) = E(\mathbf{z}) + \mathbf{g}^T(\mathbf{z})\boldsymbol{\delta} + \frac{1}{2}\boldsymbol{\delta}^T\mathbf{G}(\mathbf{z})\boldsymbol{\delta} \tag{69}$$

where $\mathbf{g}$ is the gradient vector, $\mathbf{G}$ is the Hessian matrix and $\boldsymbol{\delta}$ is the step vector. If we impose the stationary conditions for the optimization

$$\frac{\partial Q(\boldsymbol{\delta})}{\partial\boldsymbol{\delta}} = 0, \tag{70}$$

we obtain the following set of linear equations:

$$\mathbf{G}(\mathbf{z})\boldsymbol{\delta} = -\mathbf{g}(\mathbf{z}) . \tag{71}$$

We can show that this model is quadratically convergent by expanding the gradient vector

$$\mathbf{g}(\mathbf{z}+\boldsymbol{\delta}) = \cancel{\mathbf{g}(\mathbf{z})} + \cancel{\mathbf{G}(\mathbf{z})\boldsymbol{\delta}} + \mathcal{O}\left(\boldsymbol{\delta}^2\right) \tag{72}$$

which turn to scale quadratically with the error.

Convergence is guaranteed if the Hessian is positive definite, but in the initial steps this is not always the case as the quadratic approximation holds only locally close to a minimum. To strengthen the robustness of the algorithm we can introduce a Lagrange constrain to the

quadratic model such that the step is accepted only if it lies within a trusted hypervolume around the current iterate. Then, we can define a Lagrangian

$$\mathcal{L}\left(\boldsymbol{\delta},\mu\right)=Q\left(\boldsymbol{\delta}\right)-\frac{1}{2}\mu\left(\left\|\boldsymbol{\delta}\right\|^{2}-R_{t}^{2}\right) \tag{73}$$

where μ is the Lagrange multiplier and R_t the trust radius. From this Lagrangian we can obtain the active constrain condition as well as the new set of linear equations for determine the step, the so called Karush-Kuhn-Tucker (KKT) conditions:

$$\frac{\partial\mathcal{L}}{\partial\mu}=\left\|\boldsymbol{\delta}\right\|^{2}-R_{t}^{2}=0, \tag{74}$$

$$\frac{\partial\mathcal{L}}{\partial\boldsymbol{\delta}}=\mathbf{G}\left(\mathbf{z}\right)\boldsymbol{\delta}+\mathbf{g}\left(\mathbf{z}\right)-\mu\boldsymbol{\delta}=0\rightarrow\Big(\mathbf{G}\left(\mathbf{z}\right)-\mu\mathbf{I}\Big)\boldsymbol{\delta}=-\mathbf{g}\left(\mathbf{z}\right). \tag{75}$$

The role of the Lagrange multiplier μ is to shift the eigenvalues of the Hessian matrix $\mathbf{G}$ such that it becomes positive definite and convergence to a stationary minima is ensured. To this purpose, a multiplier smaller than the smallest eigenvalue of $\mathbf{G}$ is the most suitable choice. The common way of achieving this is to define an augmented Hessian

$$\tilde{\mathbf{G}}=\left(\begin{array}{ccc:c} & & & 1\\ & \mathbf{G} & & \vdots\\ & & & 1\\ \hdashline 1 & \ldots & 1 & 0\end{array}\right) \tag{76}$$

whose first eigenvalue is smaller then all the eigenvalues of $\mathbf{G}$ thanks to the Hylleraas-Undheim-MacDonald theorem. A successful variant of this approach is the NEO algorithm.

Lastly, we mention that the trust radius is updated after each iteration step by using the Fletcher method based on the ratio between the actual energy change and the one predicted by the approximate quadratic model.

S4 Hessian linear transformations

In the trust region Newton-Raphson algorithm the optimization is carried out by solving iteratively the following set of linear equations

$$\left(\mathbf{E}_n^{(2)} - \mu\mathbf{I}\right)\Delta\mathbf{z}_n = -\mathbf{E}_n^{(1)} \tag{77}$$

featuring the level-shifted Hessian at the n-th iteration ($\mathbf{E}_n^{(2)} - \mu\mathbf{I}$), the new step in the variables ($\boldsymbol{\Delta}\mathbf{z}_n$) and the local gradient ($\mathbf{E}_n^{(1)}$) defined as

$$\mathbf{E}^{(1)} = \begin{pmatrix} \partial E/\partial\boldsymbol{\kappa} \\ \partial E/\partial\boldsymbol{\eta} \end{pmatrix} \equiv \begin{pmatrix} \mathbf{f}^{\kappa} \\ \mathbf{f}^{\eta} \end{pmatrix}. \tag{78}$$

In principle, to resolve this set of equations, the Hessian matrix should be calculated at each n-th iteration step and then be inverted, but this is very computationally demanding due to the large number of the non-redudant κ-parameters. For this reason the common way for defining Newton steps is to calculate directly the Hessian linear transformations on a trial step vector

$$\boldsymbol{\sigma} = \mathbf{E}^{(2)}\Delta\mathbf{z} \tag{79}$$

and solve iteratively with a Davidson algorithm.

Considering our SC-parametrization in eq. (11) composed by two different classes of parameters, the linear transformations equations read as

$$\boldsymbol{\sigma} = \begin{pmatrix} \mathbf{E}^{\kappa\kappa} & \mathbf{E}^{\kappa\eta} \\ \mathbf{E}^{\eta\kappa} & \mathbf{E}^{\eta\eta} \end{pmatrix} \begin{pmatrix} \Delta\boldsymbol{\kappa} \\ \Delta\boldsymbol{\eta} \end{pmatrix} = \begin{pmatrix} \mathbf{E}^{\kappa\kappa}\Delta\boldsymbol{\kappa} + \mathbf{E}^{\kappa\eta}\Delta\boldsymbol{\eta} \\ \mathbf{E}^{\eta\kappa}\Delta\boldsymbol{\kappa} + \mathbf{E}^{\eta\eta}\Delta\boldsymbol{\eta} \end{pmatrix}. \tag{80}$$

In the following sections each term in eq. (80) is derived in terms of gradient elements.

S4.1 Pure $\boldsymbol{\kappa}$ linear transformations

Starting with the purely κ-transformations, we have

$$\left(\mathbf{E}^{\kappa\kappa}\Delta\boldsymbol{\kappa}\right)_{pq} = \frac{1+P_{pq,rs}}{2}\sum_{r>s}\langle\mathrm{HF},0|\left[\left[H_{\mathrm{SC}},E_{pq}^{-}\right],E_{rs}^{-}\right]|\mathrm{HF},0\rangle\,\Delta\kappa_{rs} \tag{81}$$

which can be easily rearranged as

$$\left(\mathbf{E}^{\kappa\kappa}\Delta\boldsymbol{\kappa}\right)_{pq} = 2\,\langle\mathrm{HF}|\left[\left[\langle H_{\mathrm{SC}}\rangle_0,\Delta\hat{\kappa}\right],E_{pq}\right]|HF\rangle + \langle HF|\left[\left[\Delta\hat{\kappa},E_{pq}\right],\langle H_{\mathrm{SC}}\rangle_0\right]|\mathrm{HF}\rangle \tag{82}$$

where $\langle H_{SC}\rangle_0$ is an electronic-like Hamiltonian defined in eq. (18), while $\Delta\hat{\kappa}$ is defined as

$$\Delta\hat{\kappa} = \sum_{pq}\Delta\kappa_{pq}E_{pq}. \tag{83}$$

For the first term in eq. (82) we can observe that the commutator between $\langle H_{\mathrm{SC}}\rangle_0$ and $\Delta\hat{\kappa}$ remains an electronic-like Hamiltonian

$$\langle H_{\mathrm{SC}}\rangle_0^{\Delta\kappa} = \left[\langle H_{\mathrm{SC}}\rangle_0,\Delta\hat{\kappa}\right] = \sum_{pq}h_{pq}^{b,\Delta\kappa}E_{pq} + \frac{1}{2}\sum_{pqrs}g_{pqrs}^{b,\Delta\kappa}e_{pqrs} \tag{84}$$

where the integrals in eqs. (19) and (20) (transformed back to the canonical basis) are $\Delta\kappa$-one-index transformed:

$$h_{pq}^{b,\Delta\kappa} = \sum_t(\Delta\kappa_{tp}h_{tq}^b + \Delta\kappa_{tq}h_{pt}^b), \tag{85}$$

$$g_{pqrs}^{b,\Delta\kappa} = \sum_t(\Delta\kappa_{tp}g_{tqrs}^b + \Delta\kappa_{tq}g_{ptrs}^b + \Delta\kappa_{tr}g_{pqts}^b + \Delta\kappa_{ts}g_{pqrt}^b). \tag{86}$$

For the second term in eq. (82), instead, we have

$$\begin{aligned} &\langle \mathrm{HF}| \big[\big[\Delta\hat{\kappa}, E_{pq} \big], \langle H_{\mathrm{SC}} \rangle_0 \big] \, |\mathrm{HF}\rangle = \\ &\quad = \sum_r \big(\Delta\kappa_{pr} \, \langle \mathrm{HF}| \, \big[E_{rq}, \langle H_{\mathrm{SC}} \rangle_0 \big] \, |\mathrm{HF}\rangle - \langle \mathrm{HF}| \, \big[E_{pr}, \langle H_{\mathrm{SC}} \rangle_0 \big] \, |\mathrm{HF}\rangle \, \Delta\kappa_{rq} \big). \end{aligned} \tag{87}$$

By substituting eqs. (84) and (87) in eq. (82) we end up with the following definition for the purely κ-transformations:

$$(\mathbf{E}^{\kappa\kappa} \Delta\boldsymbol{\kappa})_{pq} = f^{\kappa}_{pq}(h^{b,\Delta\kappa}_{pq}, \; g^{b,\Delta\kappa}_{pqrs}) + \frac{1}{2} \Big(\big[\Delta\boldsymbol{\kappa}, \mathbf{f}^{\kappa} \big] \Big)_{pq}. \tag{88}$$

This result is completely analogous with the one found in the Hartree Fock which is recovered by simply setting $\lambda = 0$.

S4.2 Pure $\boldsymbol{\eta}$ linear transformations

Moving to the purely η-transformations, we have

$$\left(\mathbf{E}^{(2)}_{\eta\eta}\Delta\boldsymbol{\eta}\right)_r = \frac{\lambda}{\sqrt{2\omega}} \langle \mathrm{HF}, 0| \left[\tilde{E}_{rr}(b-b^\dagger), \left[\Delta\hat{\eta}, H_{\mathrm{SC}}\right]\right] |\mathrm{HF}, 0\rangle \tag{89}$$

where $\Delta\hat{\eta}$ is defined as:

$$\Delta\hat{\eta} = \frac{\lambda}{\sqrt{2\omega}} \sum_s \Delta\eta_s \tilde{E}_{ss}(b-b^\dagger). \tag{90}$$

Calculating the first commutator we obtain the $\Delta\eta$-transformed SC-Hamiltonian:

$$\begin{aligned}
H^{\Delta\eta}_{\mathrm{SC}} &= \left[\Delta\hat{\eta}, H_{\mathrm{SC}}\right] \\
&= \frac{\lambda}{\sqrt{2\omega}} \sum_{pq} \tilde{h}_{pq}(\Delta\eta_p - \Delta\eta_q)\tilde{E}_{pq} \exp\left(\frac{\lambda}{\sqrt{2\omega}}(\eta_p-\eta_q)(b-b^\dagger)\right)(b-b^\dagger) \\
&+ \frac{\lambda}{\sqrt{2\omega}} \sum_{pqrs} \frac{\tilde{g}_{pqrs}}{2}(\Delta\eta_p - \Delta\eta_q + \Delta\eta_r - \Delta\eta_s)\tilde{e}_{pqrs} \exp\left(\frac{\lambda}{\sqrt{2\omega}}(\eta_p-\eta_q+\eta_r-\eta_s)\left(b-b^\dagger\right)\right)(b-b^\dagger) \\
&+ \lambda\sqrt{\frac{\omega}{2}} \sum_s \Delta\eta_s \tilde{E}_{ss}(b+b^\dagger) - \frac{\lambda^2}{\omega}\sum_{ps}((\tilde{\mathbf{d}}\cdot\boldsymbol{\epsilon})_{pp} - \eta_p)\Delta\eta_s(\tilde{e}_{sspp} + \tilde{E}_{ps}\delta_{ps}).
\end{aligned} \tag{91}$$

Substituting the $\Delta\eta$-transformed Hamiltonian just found inside the second commutator in eq. (54), we obtain:

$$\begin{aligned}
&\left[\tilde{E}_{rr}(b-b^\dagger), H^{\Delta\eta}_{\mathrm{SC}}\right] = \\
&= \frac{\lambda}{\sqrt{2\omega}} \sum_{pq} \tilde{h}_{pq}(\Delta\eta_p - \Delta\eta_q)\left[\tilde{E}_{rr}, \tilde{E}_{pq}\right] \exp\left(\frac{\lambda}{\sqrt{2\omega}}(\eta_p-\eta_q)(b-b^\dagger)\right)(b-b^\dagger)^2 \\
&+ \frac{\lambda}{\sqrt{2\omega}} \sum_{pqrs} \frac{\tilde{g}_{pqrs}}{2}(\Delta\eta_p - \Delta\eta_q + \Delta\eta_r - \Delta\eta_s)\left[\tilde{E}_{rr}, \tilde{e}_{pqrs}\right] \exp\left(\frac{\lambda}{\sqrt{2\omega}}(\eta_p-\eta_q+\eta_r-\eta_s)(b-b^\dagger)\right)(b-b^\dagger)^2 \\
&+ \lambda\sqrt{2\omega}\sum_s \Delta\eta_s(\tilde{e}_{rrss} + \tilde{E}_{sr}\delta_{rs}).
\end{aligned} \tag{92}$$

Expliciting the last commutators in the electronic part and averaging over the reference wave function we end up with

$$
\begin{aligned}
(\mathbf{E}^{\eta\eta}\Delta\boldsymbol{\eta})_r &= \frac{\lambda^2}{\omega}\sum_q \tilde{h}^{a,\eta}_{rq}(\Delta\eta_q - \Delta\eta_r)\tilde{D}_{rq} \\
&+ \frac{\lambda^2}{\omega}\sum_{qvz} \tilde{g}^{a,\eta}_{rqvz}(\Delta\eta_q - \Delta\eta_r + \Delta\eta_z - \Delta\eta_v)\tilde{d}_{rqvz} \\
&+ \lambda^2\sum_s \Delta\eta_s \tilde{d}_{rrss} + \lambda^2\Delta\eta_r\tilde{D}_{rr}
\end{aligned}
\tag{93}
$$

where the integrals $\tilde{h}^{a,\eta}_{pq}$ and $\tilde{g}^{a,\eta}_{pqrs}$ are

$$
\tilde{h}^{a,\eta}_{pq} = \tilde{h}^{a}_{pq}\Big(1 - \frac{\lambda^2}{2\omega}\left(\eta_p - \eta_q\right)^2\Big),
\tag{94}
$$

$$
\tilde{g}^{a,\eta}_{pqrs} = \tilde{g}^{a}_{pqrs}\Big(1 - \frac{\lambda^2}{2\omega}\left(\eta_p - \eta_q + \eta_r - \eta_s\right)^2\Big).
\tag{95}
$$

Comparing eq. (93) with the one for the η-gradient elements in eq. (55) we are suggested to rewrite the purely η-transformations as

$$
(\mathbf{E}^{\eta\eta}\Delta\boldsymbol{\eta})_r = \tilde{f}^{\Delta\eta}_r(\tilde{h}^{a,\eta}_{pq},\ \tilde{g}^{a,\eta}_{pqrs}) + \lambda^2\sum_q(\tilde{\mathbf{d}}\cdot\boldsymbol{\epsilon})_{qq}\tilde{d}_{qqrr} + \lambda^2(\tilde{\mathbf{d}}\cdot\boldsymbol{\epsilon})_{rr}\tilde{D}_{rr}
\tag{96}
$$

where we see the presence of the $\Delta\eta$-gradient calculated with η-transformed integrals. The same result can of course be obtained by directly contracting one index of the η-η Hessian in eq. (54) with the η-parameters.

S4.3 Mixed $\boldsymbol{\kappa}$ linear transformations

Jumping to the mixed parameter block contribute to the linear transformations in eq. (80), we notice that this block is either contracted with the κ-parameters or the η-parameters.

Starting with the transformations of the κ-parameters, we have

$$(\mathbf{E}^{\eta\kappa}\Delta\boldsymbol{\kappa})_r = \sum_{pq}\Delta\kappa_{pq}\sum_{tu}U^*_{tp}\tilde{H}_{r,tu}U_{uq} = \sum_{tu}\Delta\tilde{\kappa}_{tu}\tilde{H}_{r,tu} \tag{97}$$

where $\Delta\tilde{\kappa}_{pq}$ are the κ-steps transformed to the dipole basis. Proceeding with the calculation of the last equation directly through the definition of $\tilde{H}_{r,tu}$ in eq. (66), we obtain

$$\begin{aligned}
(\mathbf{E}^{\eta\kappa}\Delta\boldsymbol{\kappa})_r &= 2\frac{\lambda^2}{\omega}\sum_{q}\tilde{h}^c_{rq}\sum_t(\Delta\tilde{\kappa}_{rt}\tilde{D}_{tq} + \Delta\tilde{\kappa}_{qt}\tilde{D}_{rt}) \\
&+ 2\frac{\lambda^2}{\omega}\sum_{qvz}\tilde{g}^c_{rqvz}\sum_t(\Delta\tilde{k}_{rt}\tilde{d}_{tqvz} + \Delta\tilde{k}_{qt}\tilde{d}_{rtvz} + \Delta\tilde{k}_{vt}\tilde{d}_{rqtz} + \Delta\tilde{k}_{st}\tilde{d}_{rqvt}) \\
&- 2\lambda^2\sum_p((\tilde{\mathbf{d}}\cdot\boldsymbol{\epsilon})_{pp} - \eta_p)\sum_t(\Delta\tilde{k}_{rt}\tilde{d}_{trpp} + \Delta\tilde{k}_{rt}\tilde{d}_{rtpp} + \Delta\tilde{k}_{pt}\tilde{d}_{rrtp} + \Delta\tilde{k}_{pt}\tilde{d}_{rrpt}) \\
&- 2\lambda^2((\tilde{\mathbf{d}}\cdot\boldsymbol{\epsilon})_{rr} - \eta_r)\sum_t(\Delta\tilde{\kappa}_{tr}\tilde{D}_{rt} + \Delta\tilde{\kappa}_{tr}\tilde{D}_{tr})
\end{aligned} \tag{98}$$

which, comparing it with the η-gradient equation in eq. (55), can be rewritten as two times the η-gradient calculated using $\Delta\kappa$-one-index transformed density matrices:

$$(\mathbf{E}^{\eta\kappa}\Delta\boldsymbol{\kappa})_r = 2\tilde{f}^{\Delta\eta}_r(\mathbf{D}^{\Delta\kappa},\ \mathbf{d}^{\Delta\kappa}) \tag{99}$$

with

$$\tilde{D}^{\Delta\kappa}_{pq} = \sum_t(\Delta\tilde{\kappa}_{pt}\tilde{D}_{tq} + \Delta\tilde{\kappa}_{qt}\tilde{D}_{pt}), \tag{100}$$

$$\tilde{d}^{\Delta\kappa}_{pqrs} = \sum_t(\Delta\tilde{\kappa}_{pt}\tilde{d}_{tqrs} + \Delta\tilde{\kappa}_{qt}\tilde{d}_{ptrs}\Delta\tilde{\kappa}_{rt}\tilde{d}_{pqts} + \Delta\tilde{\kappa}_{st}\tilde{d}_{pqrt}). \tag{101}$$

S4.4 Mixed $\boldsymbol{\eta}$ linear transformations

Finally, moving to the transformation of the η-parameters through the mixed block, we have

$$(\mathbf{E}^{\kappa\eta}\Delta\boldsymbol{\eta})_{pq} = \lambda\sqrt{\frac{2}{\omega}}\sum_r \langle \mathrm{HF},0|\left[\left[\tilde{E}_{rr}(b-b^\dagger), H_{\mathrm{SC}}\right], E_{pq}\right]|\mathrm{HF},0\rangle\,\Delta\eta_r. \tag{102}$$

Using eq. (41) for the inner commutator we obtain

$$\begin{aligned}(\mathbf{E}^{\kappa\eta}\Delta\boldsymbol{\eta})_{pq} = \lambda\sqrt{\frac{2}{\omega}}\sum_r \Delta\eta_r\Big(&\langle \mathrm{HF},0|\left[\tilde{E}_{rr}\left[(b-b^\dagger), H_{\mathrm{SC}}\right], E_{pq}\right]|\mathrm{HF},0\rangle \\ &+\langle \mathrm{HF},0|\left[\left[\tilde{E}_{rr}, H_{\mathrm{SC}}\right](b-b^\dagger), E_{pq}\right]|\mathrm{HF},0\rangle\Big)\end{aligned} \tag{103}$$

where for the first term only the bilinear term of the SC-transformed Hamiltonian contributes, while for the second only the electronic one in eq. (16) does.

For the second addend in eq. (103): expliciting the inner commutator, taking the vacuum average and exploiting the particle antisymmetry of eqs. (63) and (64), we obtain

$$\sum_r \Delta\eta_r\,\langle \mathrm{HF},0|\left[\left[\tilde{E}_{rr}, H_{\mathrm{SC}}\right](b-b^\dagger), E_{pq}\right]|\mathrm{HF},0\rangle = \frac{\lambda}{\sqrt{2\omega}}\langle \mathrm{HF}|\left[H^{\#}_{tmp}, E_{pq}\right]|\mathrm{HF}\rangle \tag{104}$$

where

$$H^{\#}_{tmp} = \sum_{rq}\tilde{h}^c_{rq}(\Delta\eta_r - \Delta\eta_q)\tilde{E}_{rq} + \frac{1}{2}\sum_{rqvz}\tilde{g}^c_{rqvz}(\Delta\eta_r - \Delta\eta_q + \Delta\eta_v - \Delta\eta_z)\tilde{e}_{rqvz} \tag{105}$$

is an electronic-like Hamiltonian inside the definition of an Hartree Fock κ-gradient.

For the first addend in eq. (103): proceeding as we did before we obtain

$$\sum_r \Delta\eta_r \langle \mathrm{HF}, 0| \big[\tilde{E}_{rr}\big[(b-b^\dagger), \tilde{H}_{SC}\big], E_{pq}\big] |\mathrm{HF}, 0\rangle = \\ = -\lambda\sqrt{2\omega}\,\langle \mathrm{HF}| \,\big[\sum_{rs}((\tilde{\mathbf{d}}\cdot\boldsymbol{\epsilon})_{rr} - \eta_r)\Delta\eta_s(\tilde{e}_{rrss} + \tilde{E}_{rs}\delta_{rs}), E_{pq}\big] |\mathrm{HF}\rangle\,. \tag{106}$$

To further continue with the derivation is useful to give the particle symmetry carried by the electronic operators on left hand side of the commutator to the product $((\tilde{\mathbf{d}}\cdot\boldsymbol{\epsilon})_{rr} - \eta_r)\Delta\eta_s$:

$$\sum_{rs}((\tilde{\mathbf{d}}\cdot\boldsymbol{\epsilon})_{rr} - \eta_r)\Delta\eta_s\ \tilde{e}_{rrss} = \frac{1}{2}\sum_{rs}\big(((\tilde{\mathbf{d}}\cdot\boldsymbol{\epsilon})_{rr} - \eta_r)\Delta\eta_s + ((\tilde{\mathbf{d}}\cdot\boldsymbol{\epsilon})_{ss} - \eta_s)\Delta\eta_r\big)\tilde{e}_{rrss}. \tag{107}$$

Using this symmetrization is possible to reabsorb all the first addend inside the second in eq. (104) redefining the one and two electronic integrals as follows:

$$\sum_r \langle \mathrm{HF}, 0| \big[\big[\tilde{E}_{rr}(b-b^\dagger), H_{\mathrm{SC}}\big], E_{pq}\big] |\mathrm{HF}, 0\rangle\,\Delta\eta_r = \frac{\lambda}{\sqrt{2\omega}}\langle \mathrm{HF}| \big[H_e^{\#}, E_{pq}\big] |\mathrm{HF}\rangle \tag{108}$$

where the new electronic-like Hamiltonian $H_{el}^{\#}$ is

$$H_e^{\#} = \sum_{pq}\tilde{h}_{pq}^{\#}\tilde{E}_{pq} + \frac{1}{2}\sum_{pqrs}\tilde{g}_{pqrs}^{\#}\tilde{e}_{pqrs} \tag{109}$$

with

$$\tilde{h}_{pq}^{\#} = \tilde{h}_{pq}^{c}(\Delta\eta_p - \Delta\eta_q) - 2\omega((\tilde{\mathbf{d}}\cdot\boldsymbol{\epsilon})_{pp} - \eta_p)\Delta\eta_p\delta_{pq}, \tag{110}$$

$$\tilde{g}_{pqrs}^{\#} = \tilde{g}_{pqrs}^{c}(\Delta\eta_p - \Delta\eta_q + \Delta\eta_r - \Delta\eta_s) - 2\omega\big(((\tilde{\mathbf{d}}\cdot\boldsymbol{\epsilon})_{pp} - \eta_p)\Delta\eta_r + ((\tilde{\mathbf{d}}\cdot\boldsymbol{\epsilon})_{rr} - \eta_r)\Delta\eta_p\big)\delta_{pq}\delta_{rz}. \tag{111}$$

Finally, we can substitute eq. (108) in eq. (102) and obtain

$$\left(\mathbf{E}^{\kappa\eta}\Delta\boldsymbol{\eta}\right)_{pq} = \frac{\lambda^2}{\omega}\left\langle \mathrm{HF}\right| \left[H_e^{\#}, E_{pq}\right] \left|\mathrm{HF}\right\rangle \tag{112}$$

in which we recognise the κ-gradient calculated with the transformed integrals $\tilde{h}_{pq}^{\#}$ and $\tilde{g}_{pqrs}^{\#}$ transformed back to the canonical basis:

$$\left(\mathbf{E}^{\kappa\eta}\Delta\boldsymbol{\eta}\right)_{pq} = \frac{\lambda^2}{2\omega} f_{pq}^{\kappa}(h_{pq}^{\#},\ g_{pqrs}^{\#}) \tag{113}$$

where

$$h_{pq}^{\#} = \sum_{tu} U_{tp}\tilde{h}_{tu}^{\#}U_{uq}^{*}, \tag{114}$$

$$g_{pqrs}^{\#} = \sum_{tuvz} U_{tp}U_{vr}\tilde{g}_{tuvz}^{\#}U_{uq}^{*}U_{zs}^{*}. \tag{115}$$

S5 Algorithms convergence comparison

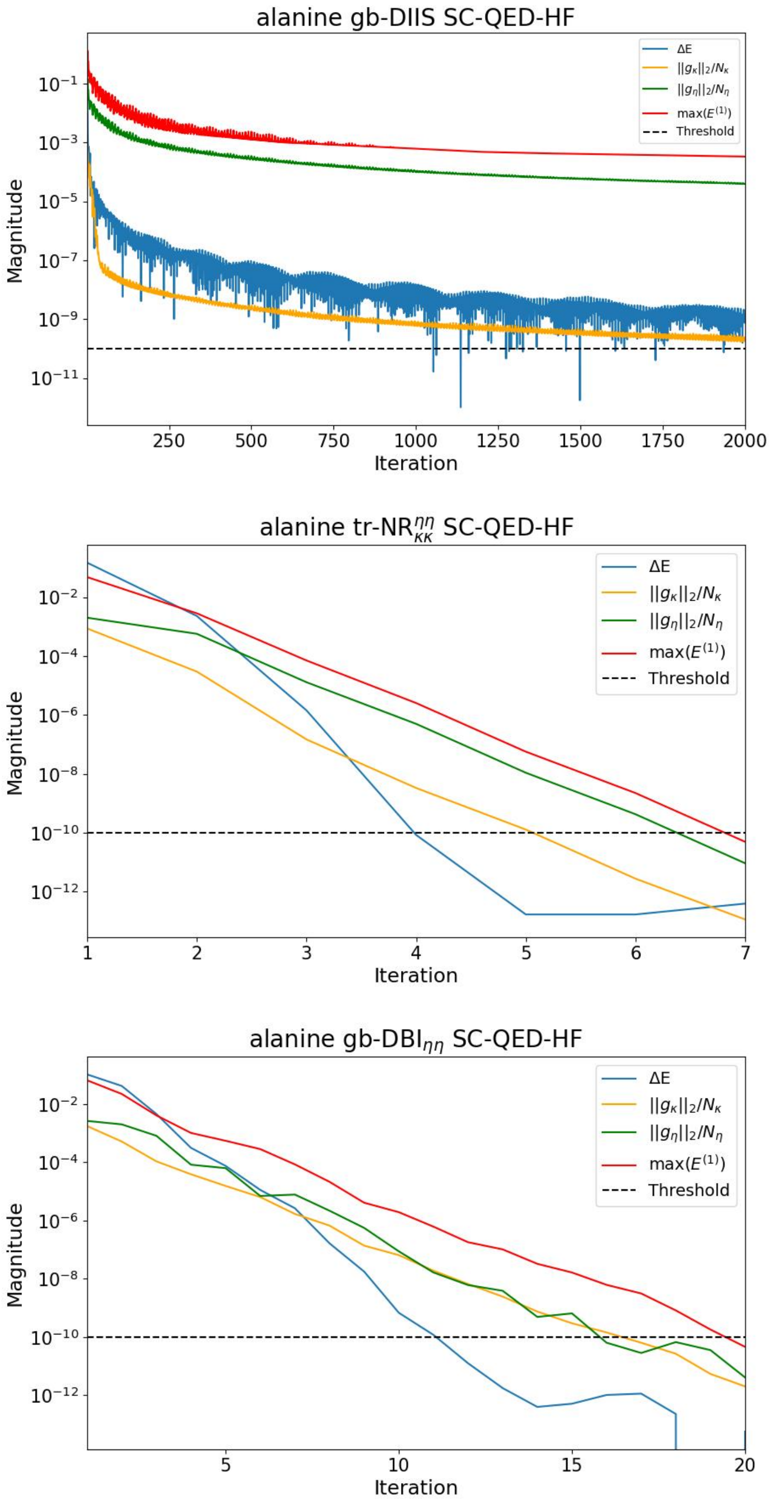

Figure S1: alanine convergence comparison

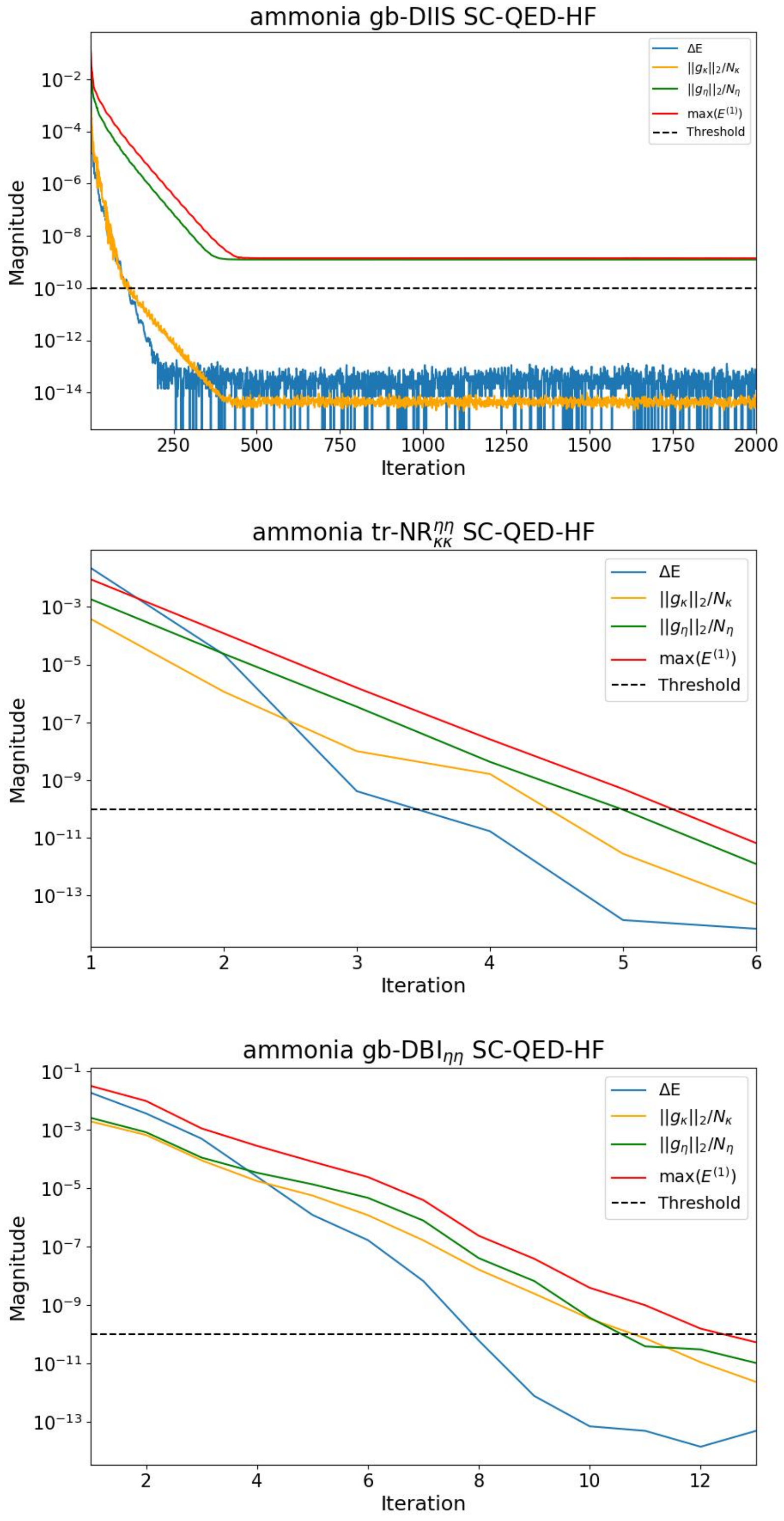

Figure S2: ammonia convergence comparison

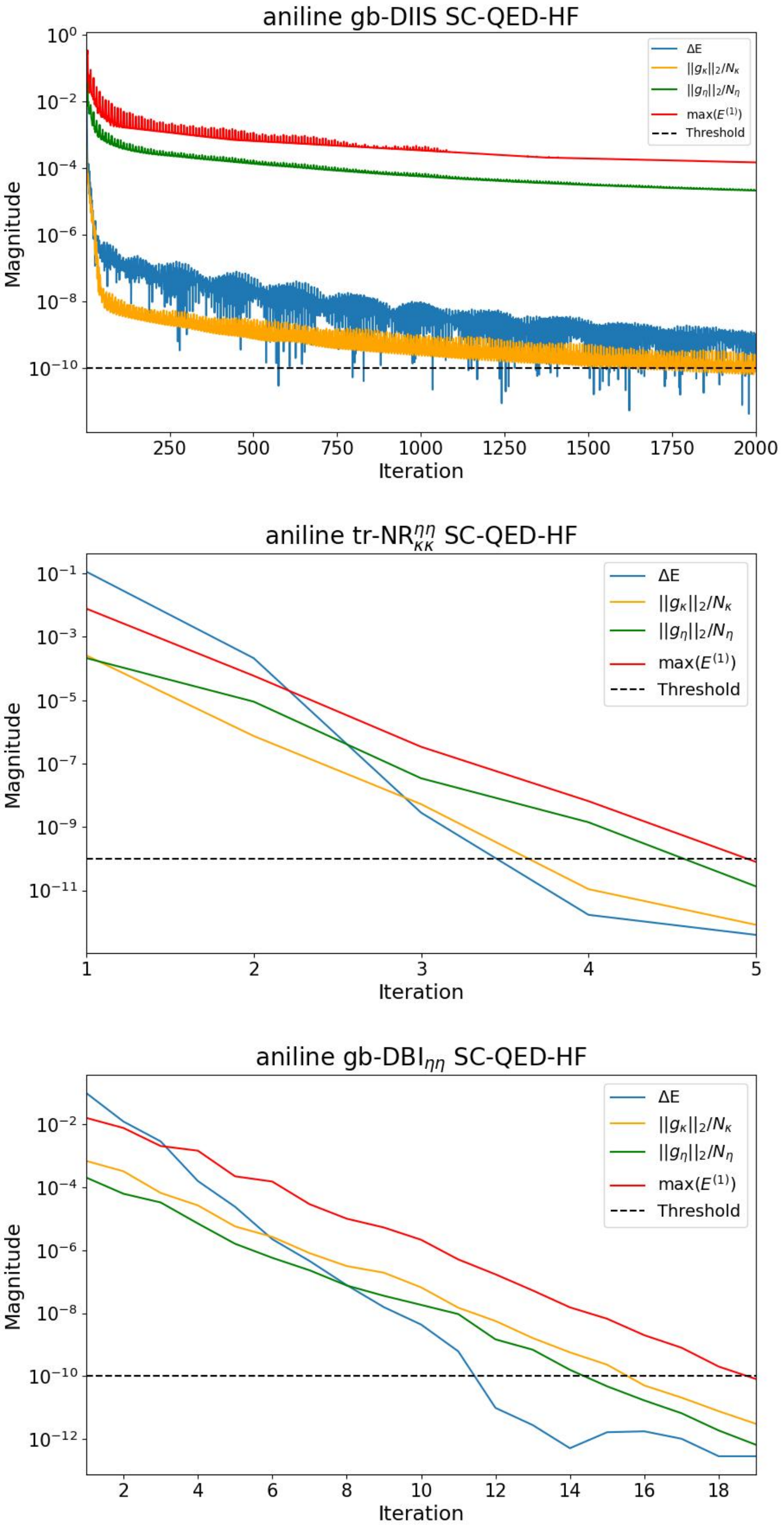

Figure S3: aniline convergence comparison

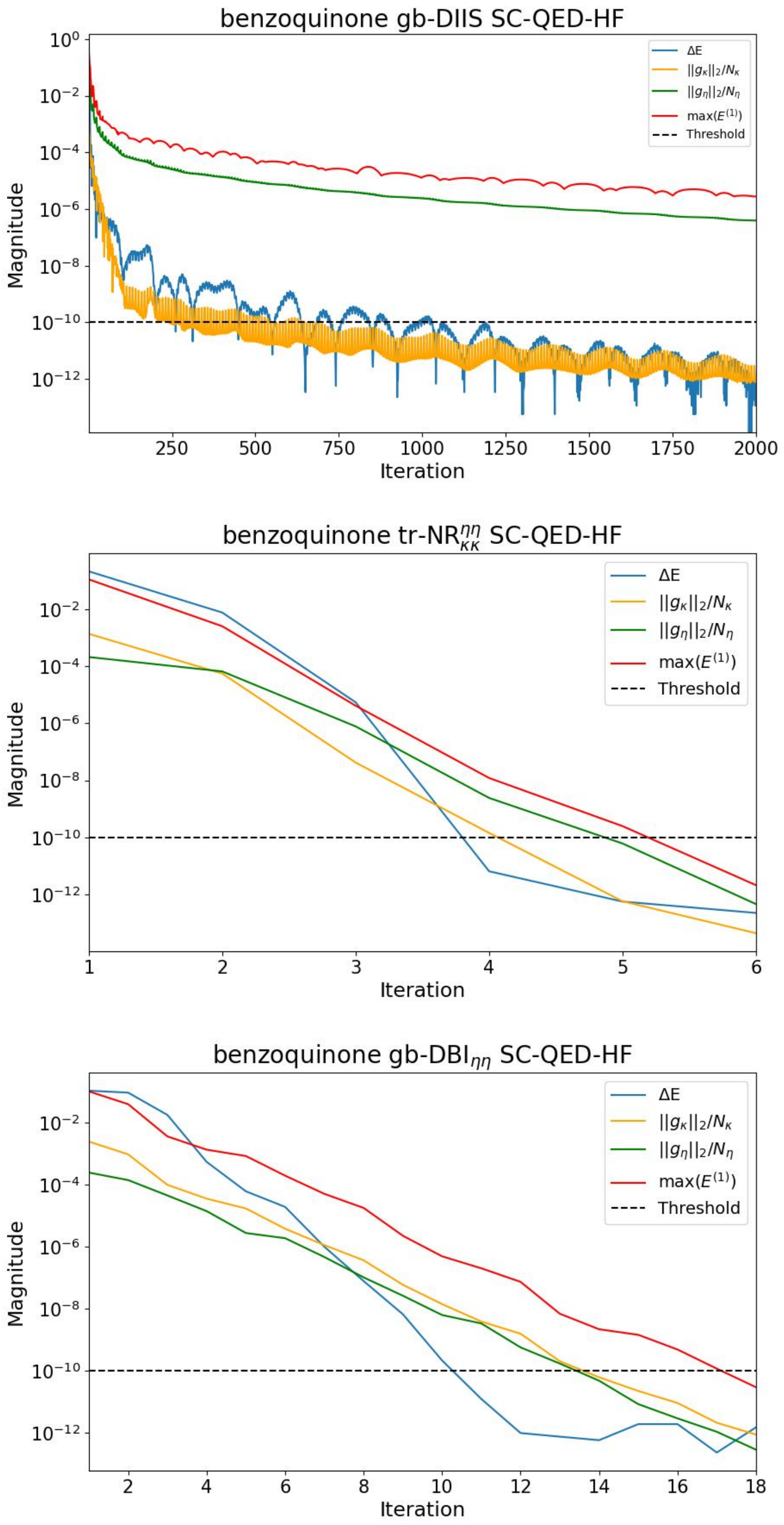

Figure S4: benzoquinone convergence comparison

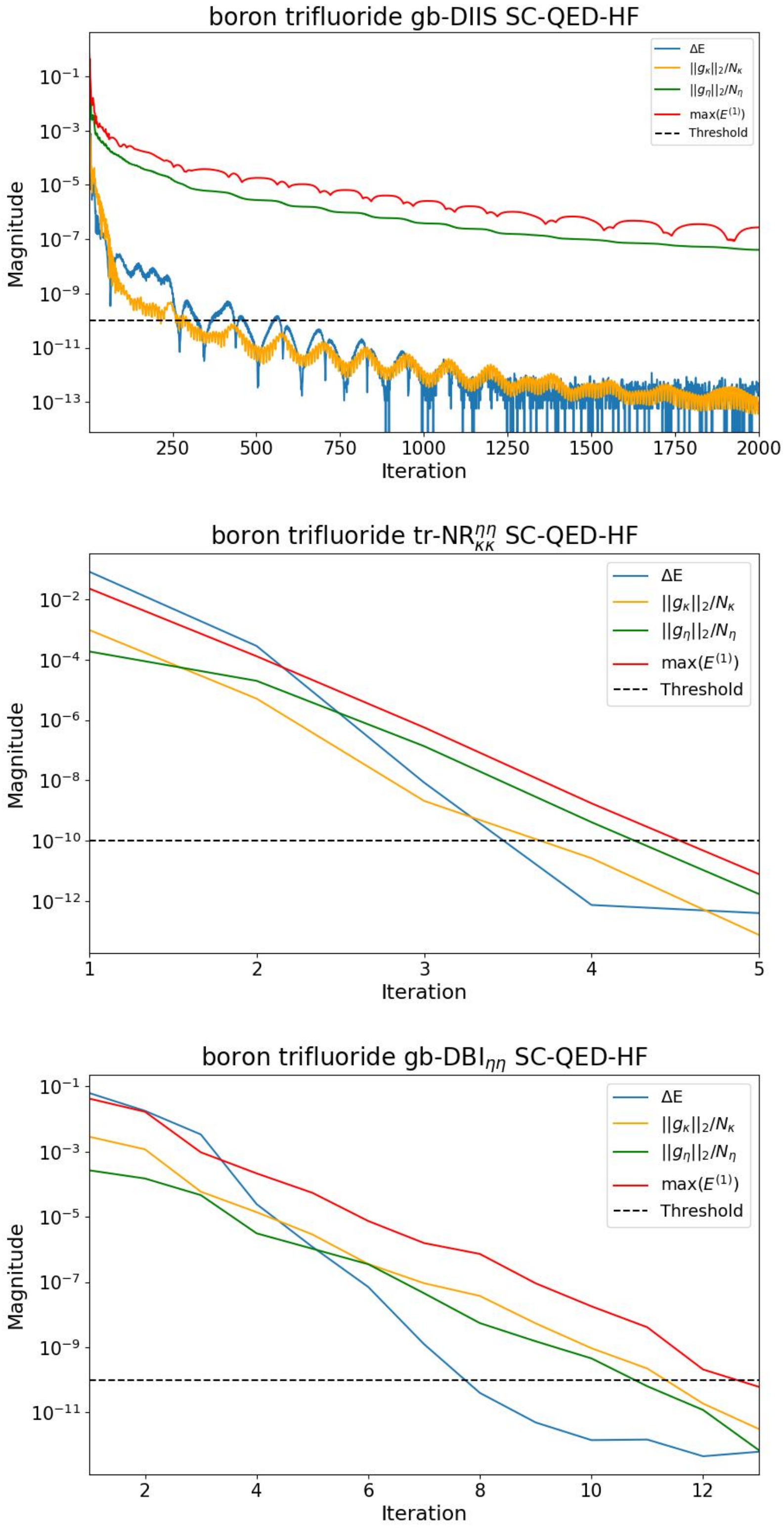

Figure S5: boron trifluoride convergence comparison

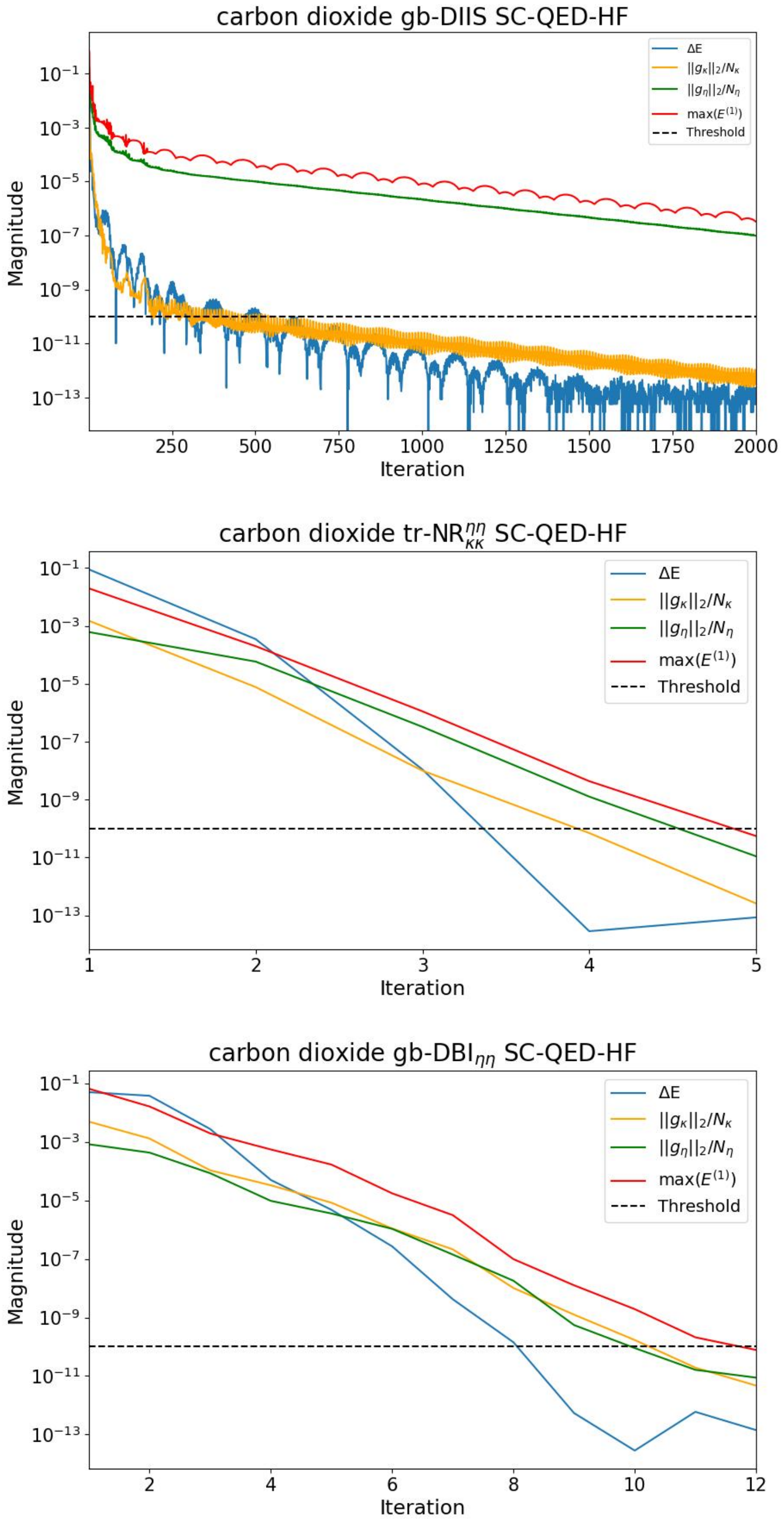

Figure S6: carbon dioxide convergence comparison

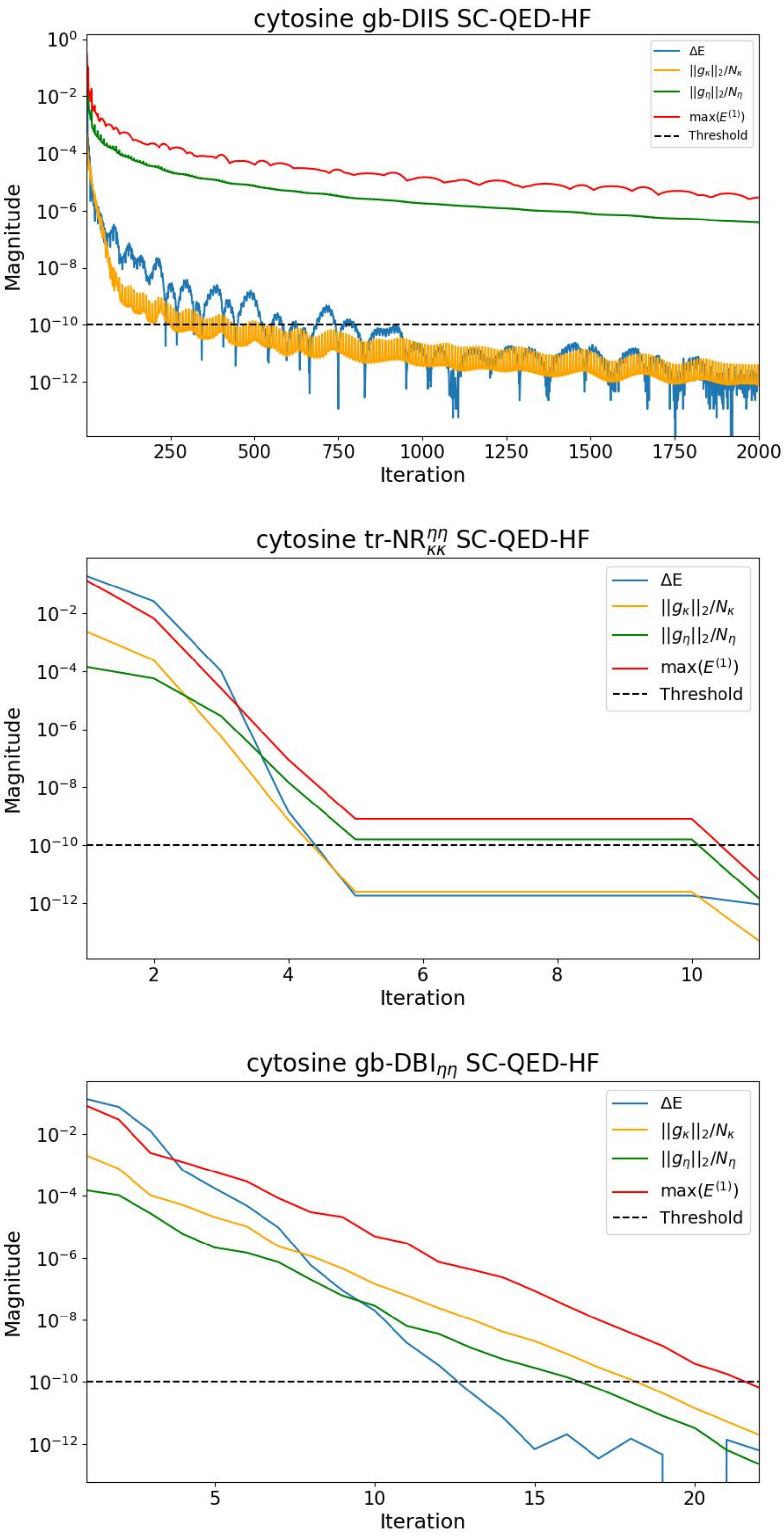

Figure S7: cytosine convergence comparison

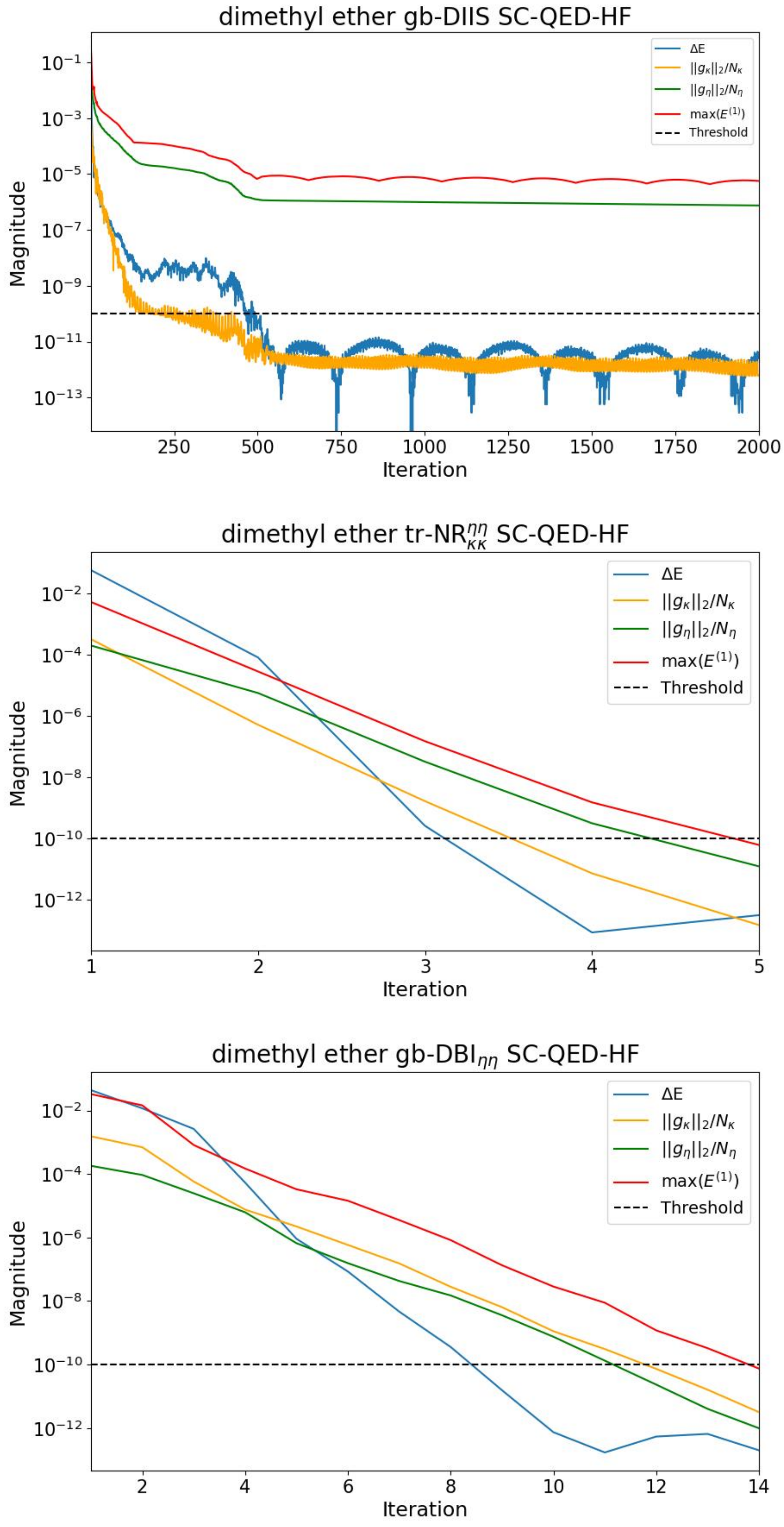

Figure S8: dimethyl ether convergence comparison

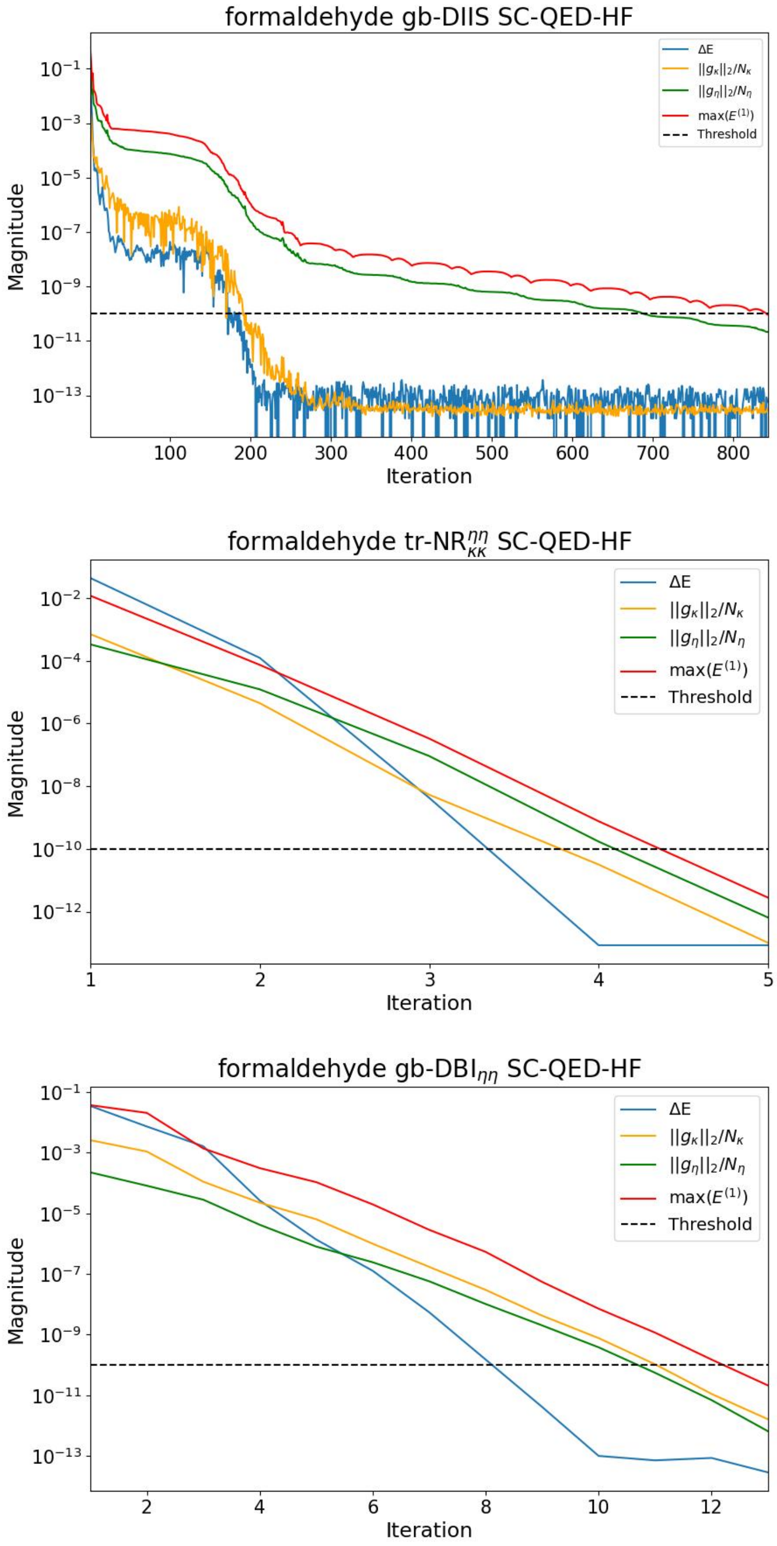

Figure S9: formaldehyde convergence comparison

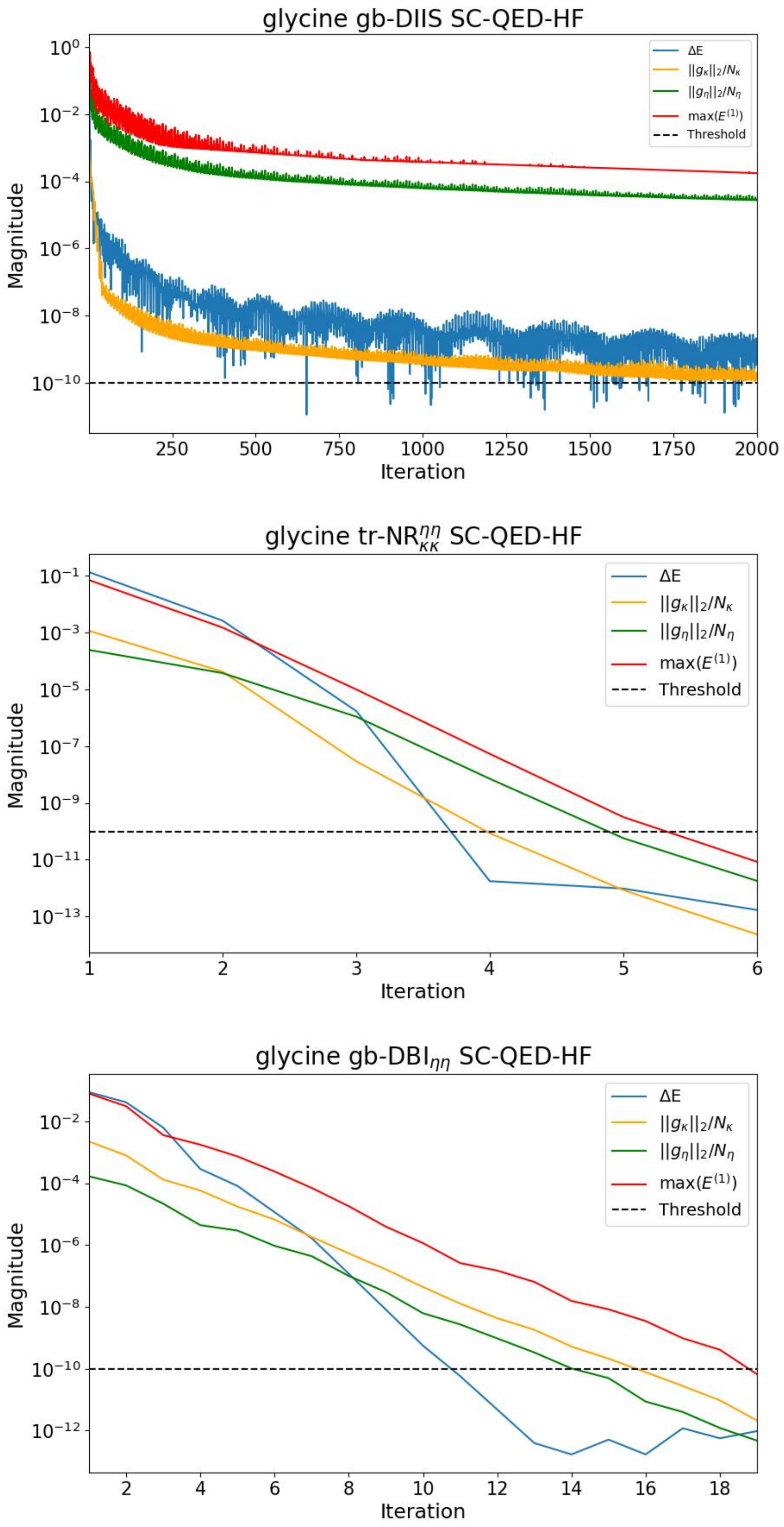

Figure S10: glycine convergence comparison

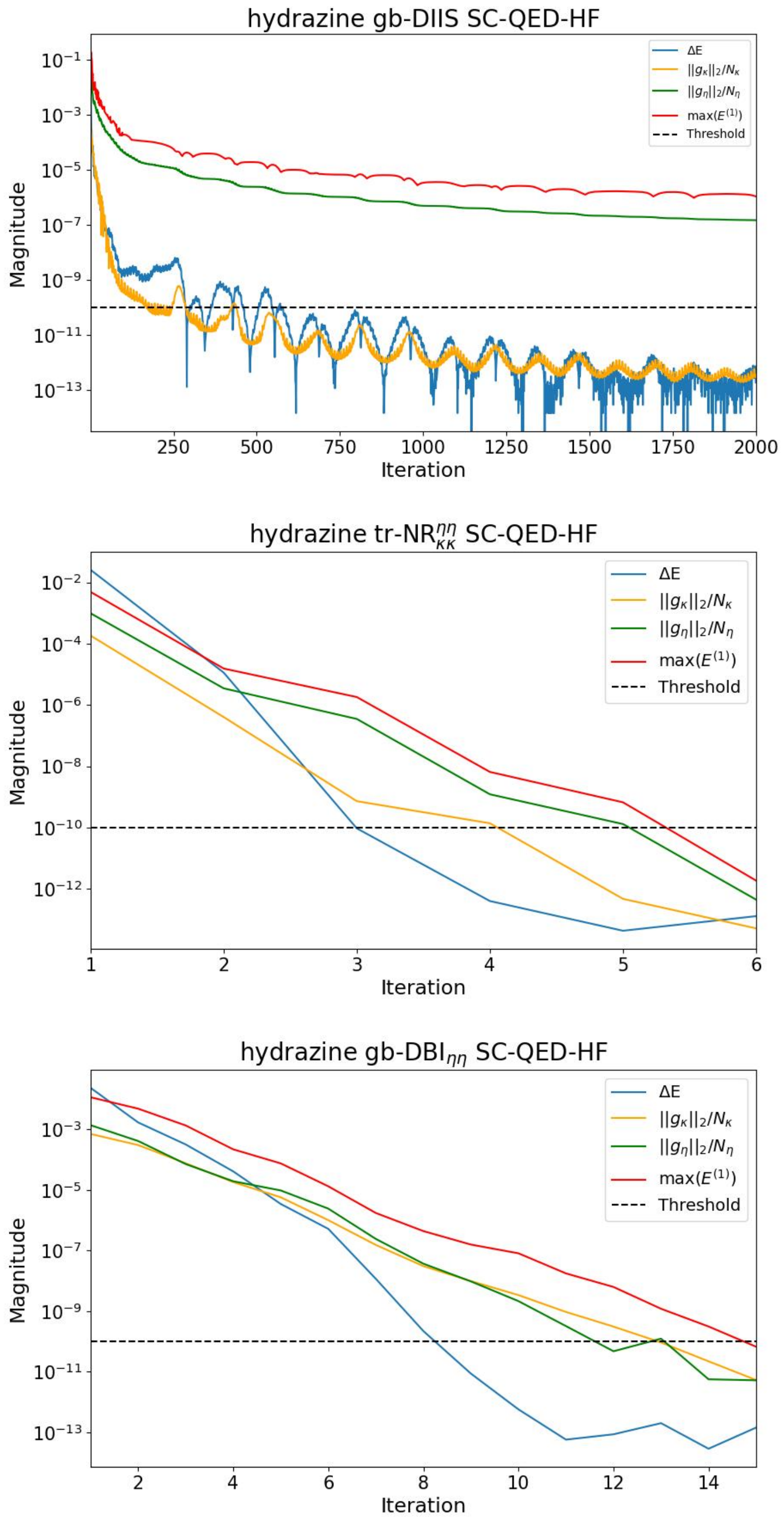

Figure S11: hydrazine convergence comparison

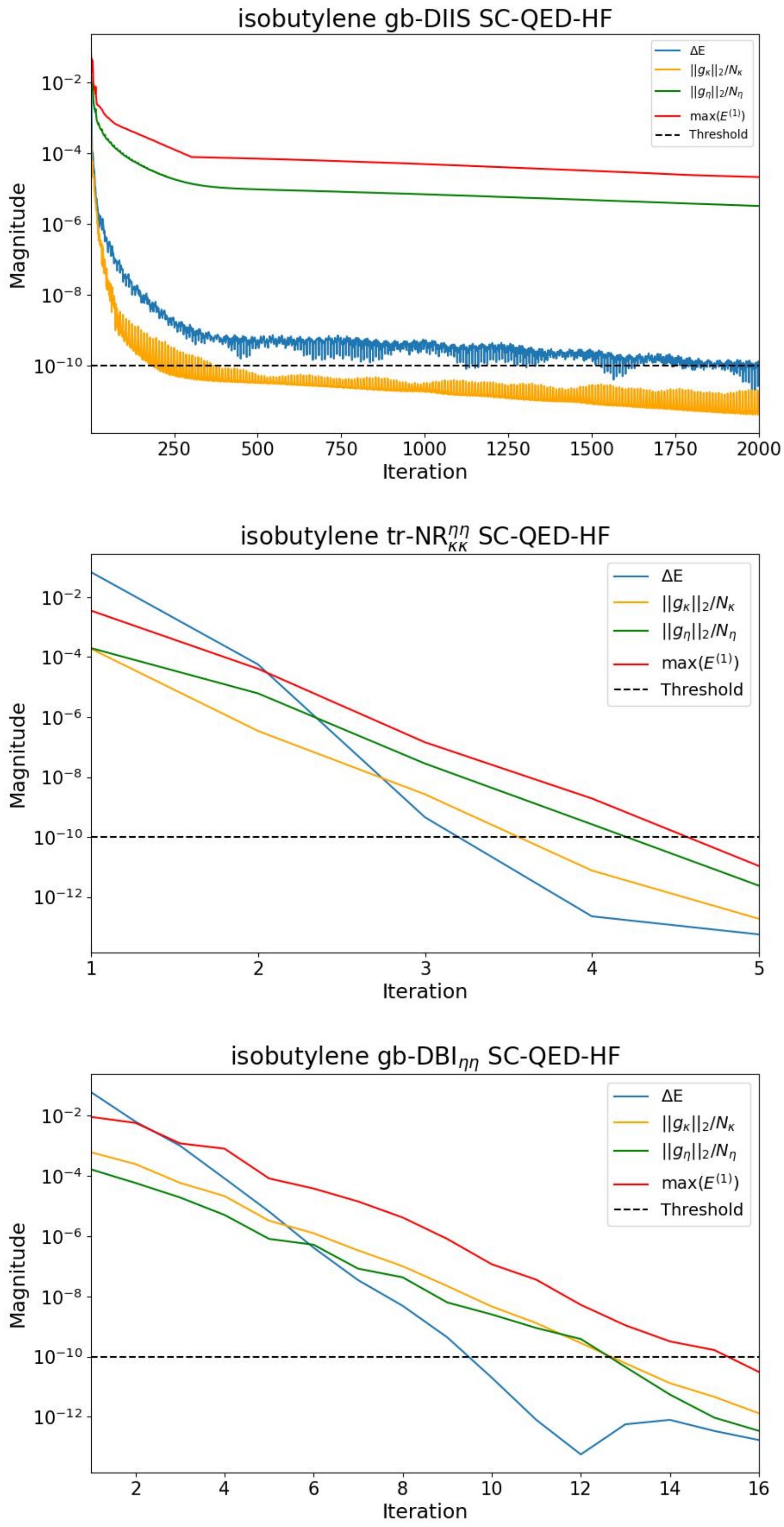

Figure S12: isobutylene convergence comparison

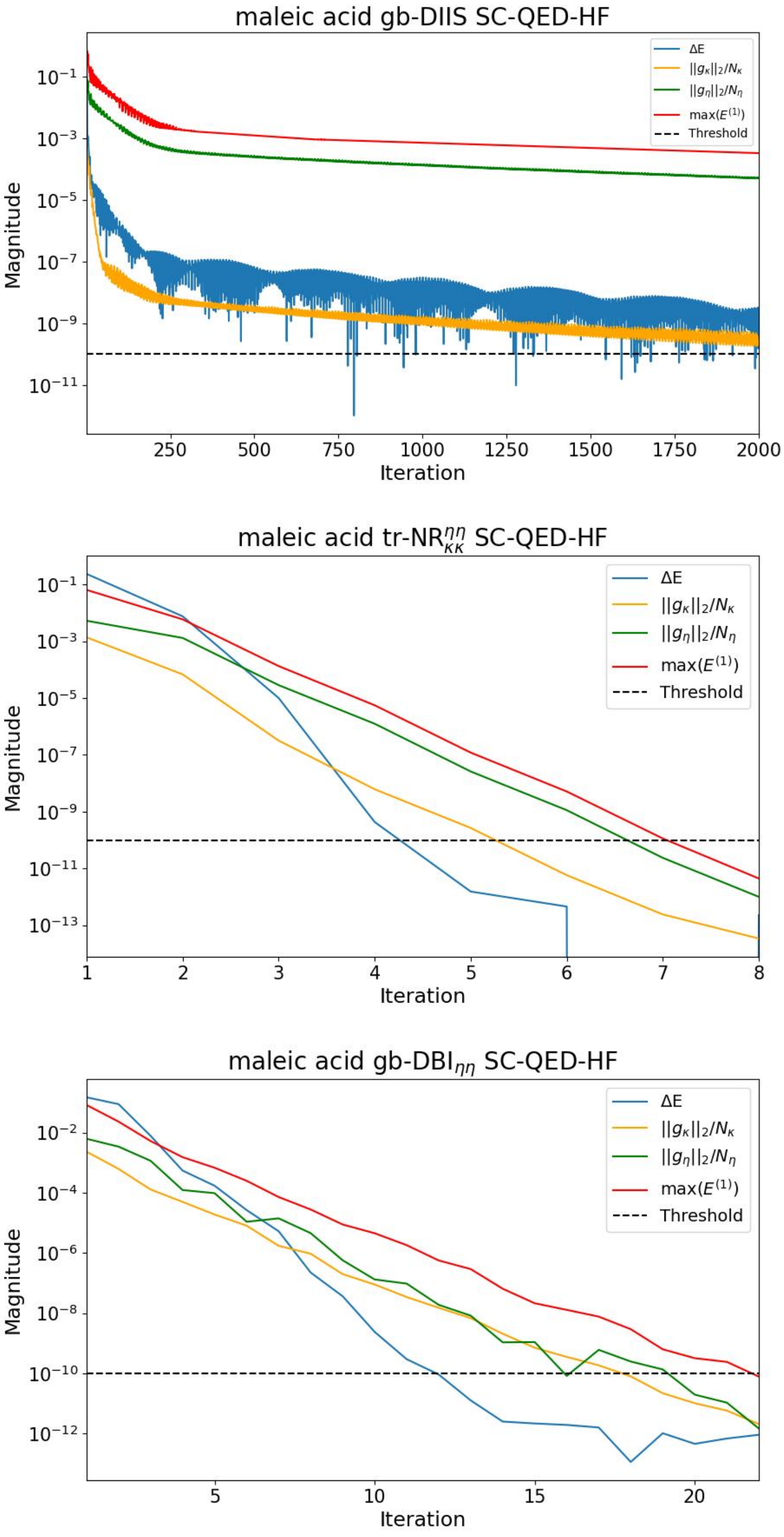

Figure S13: maleic acid convergence comparison

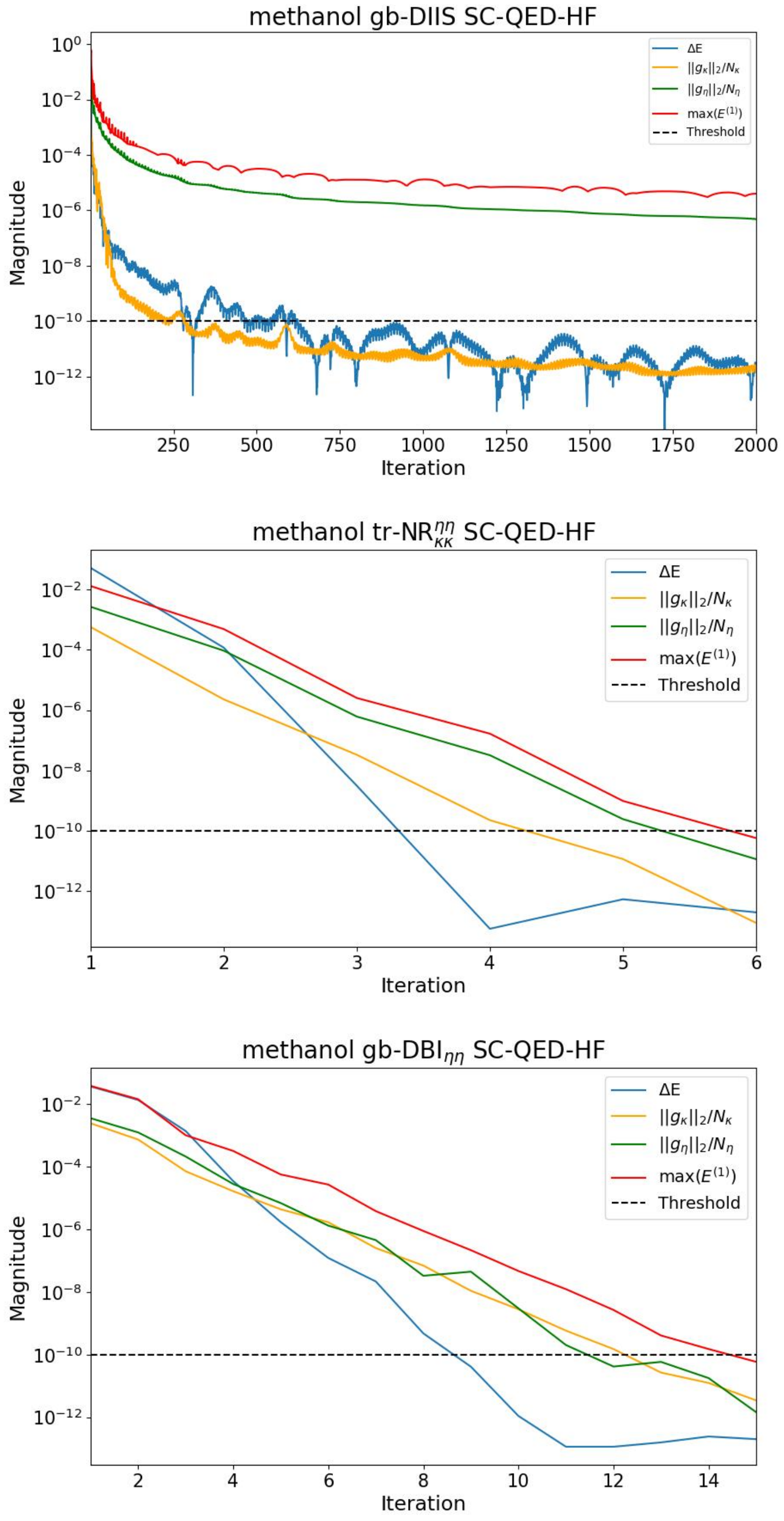

Figure S14: methanol convergence comparison

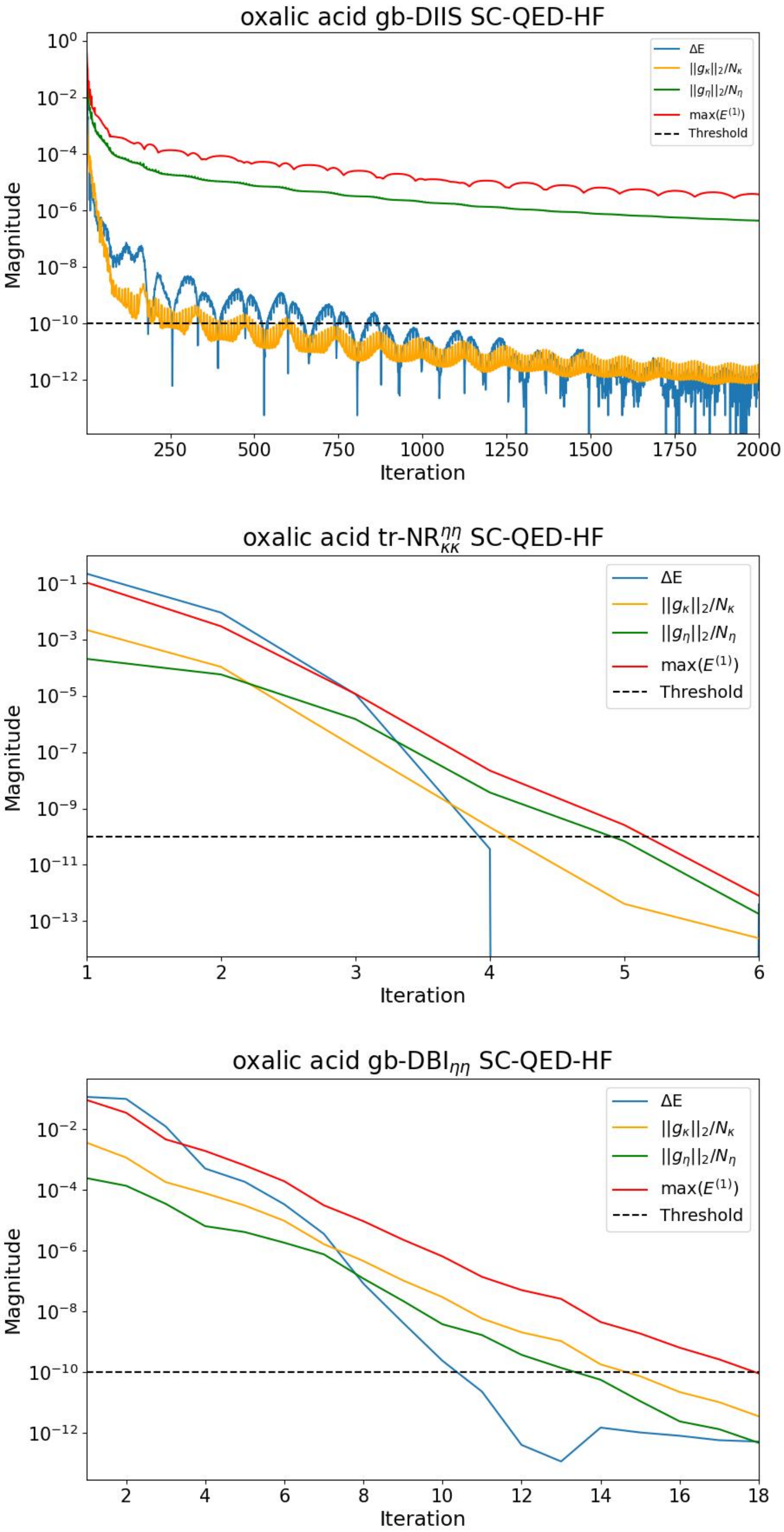

Figure S15: oxalic acid convergence comparison

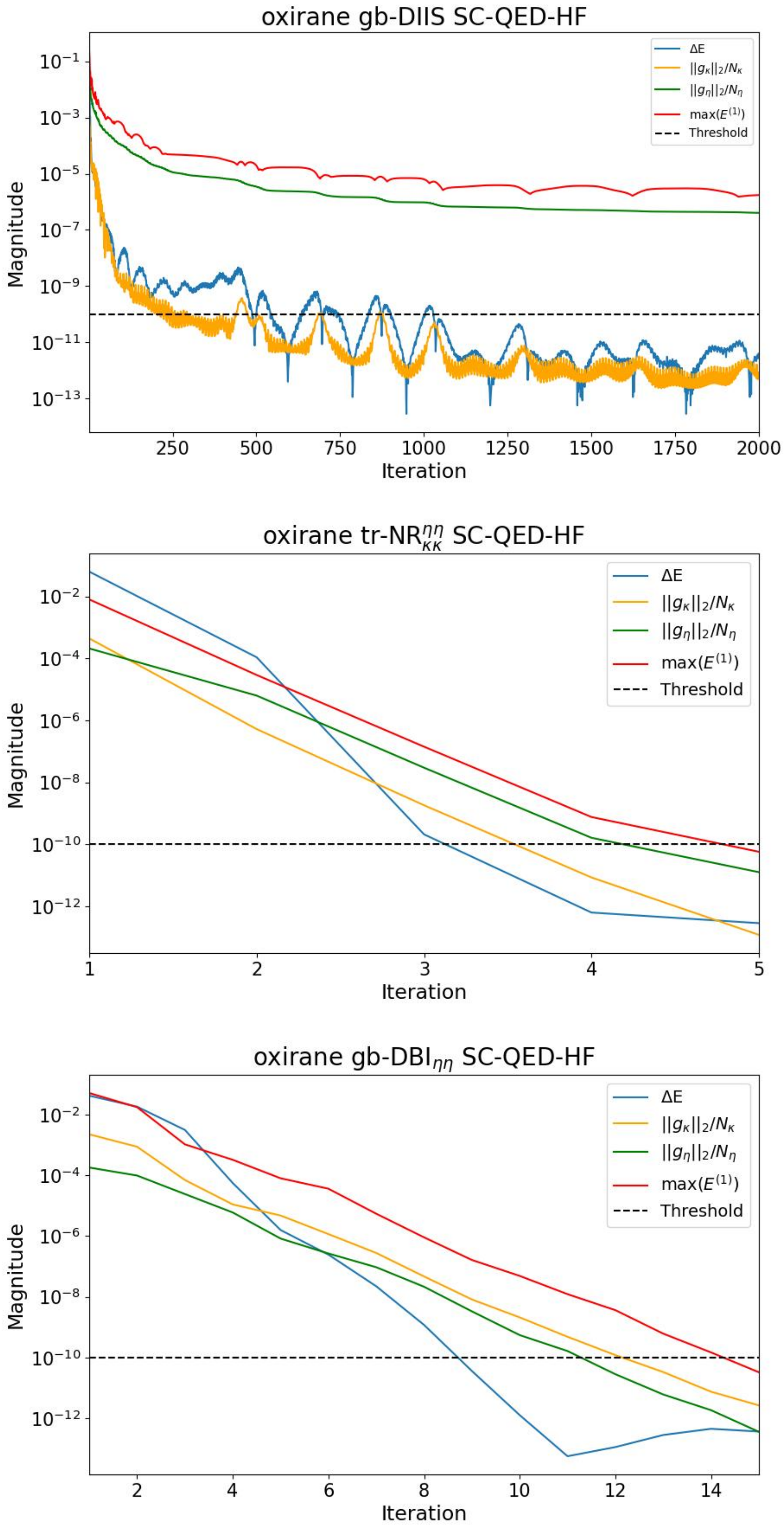

Figure S16: oxirane convergence comparison

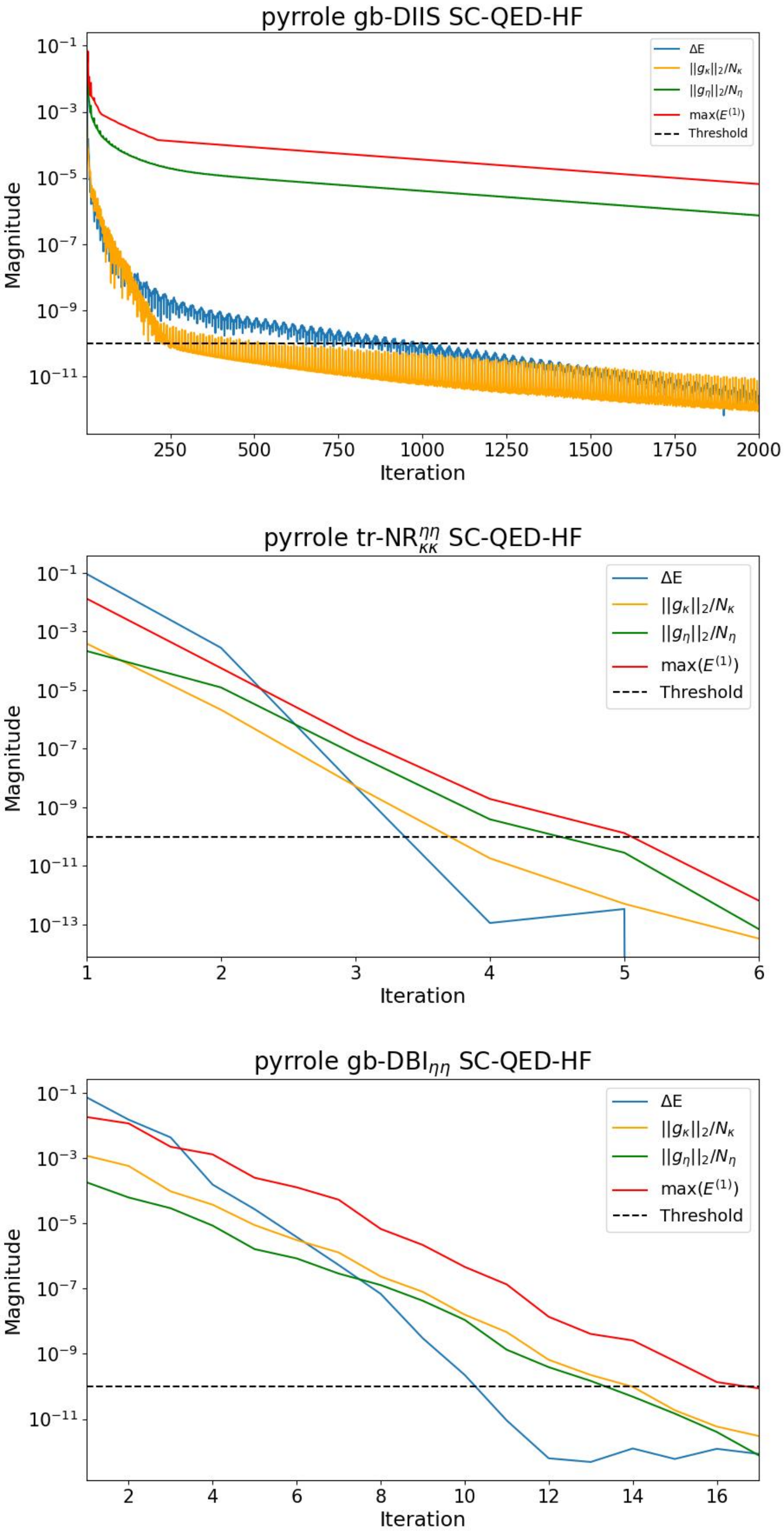

Figure S17: pyrrole convergence comparison

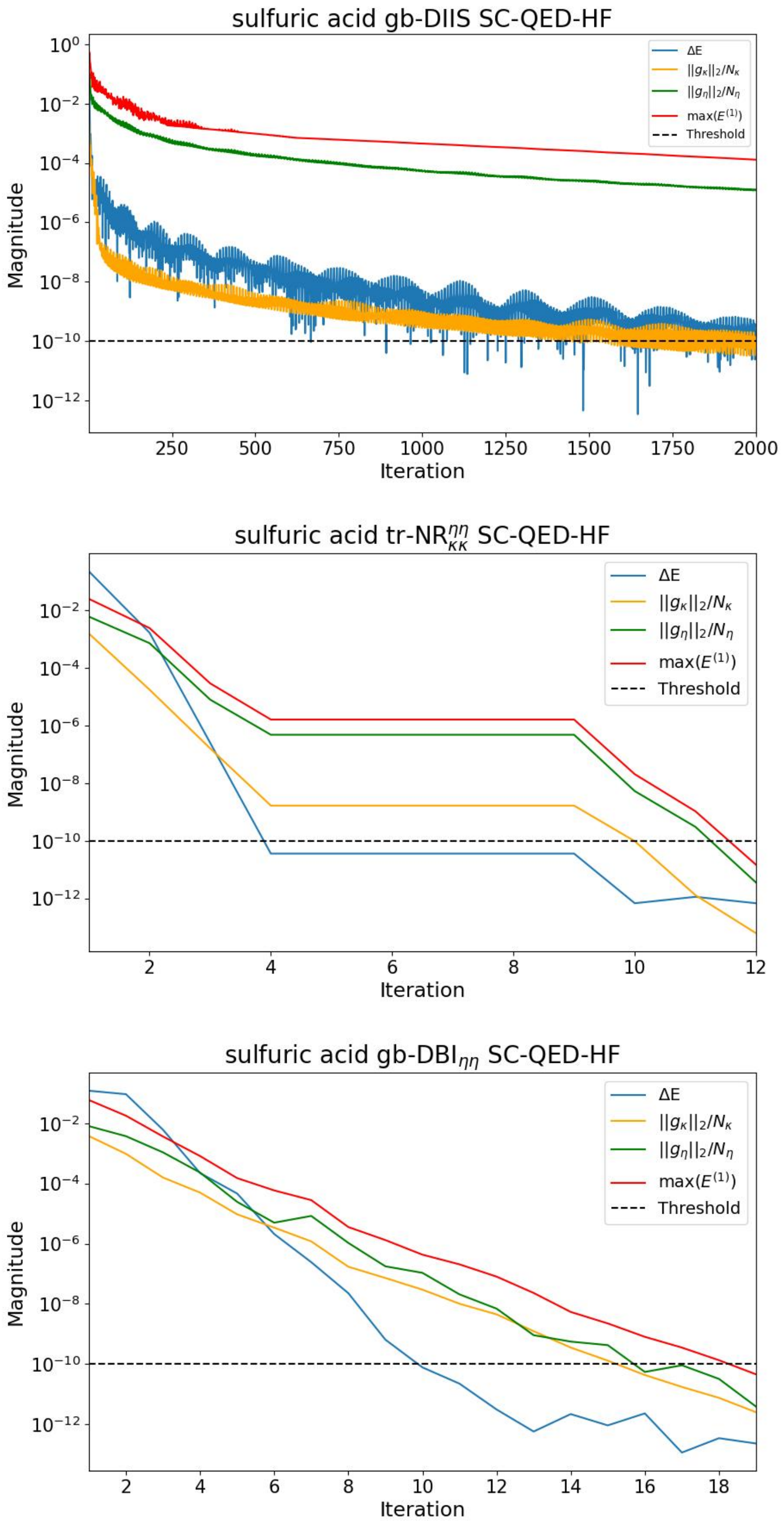

Figure S18: sulfuric acid convergence comparison

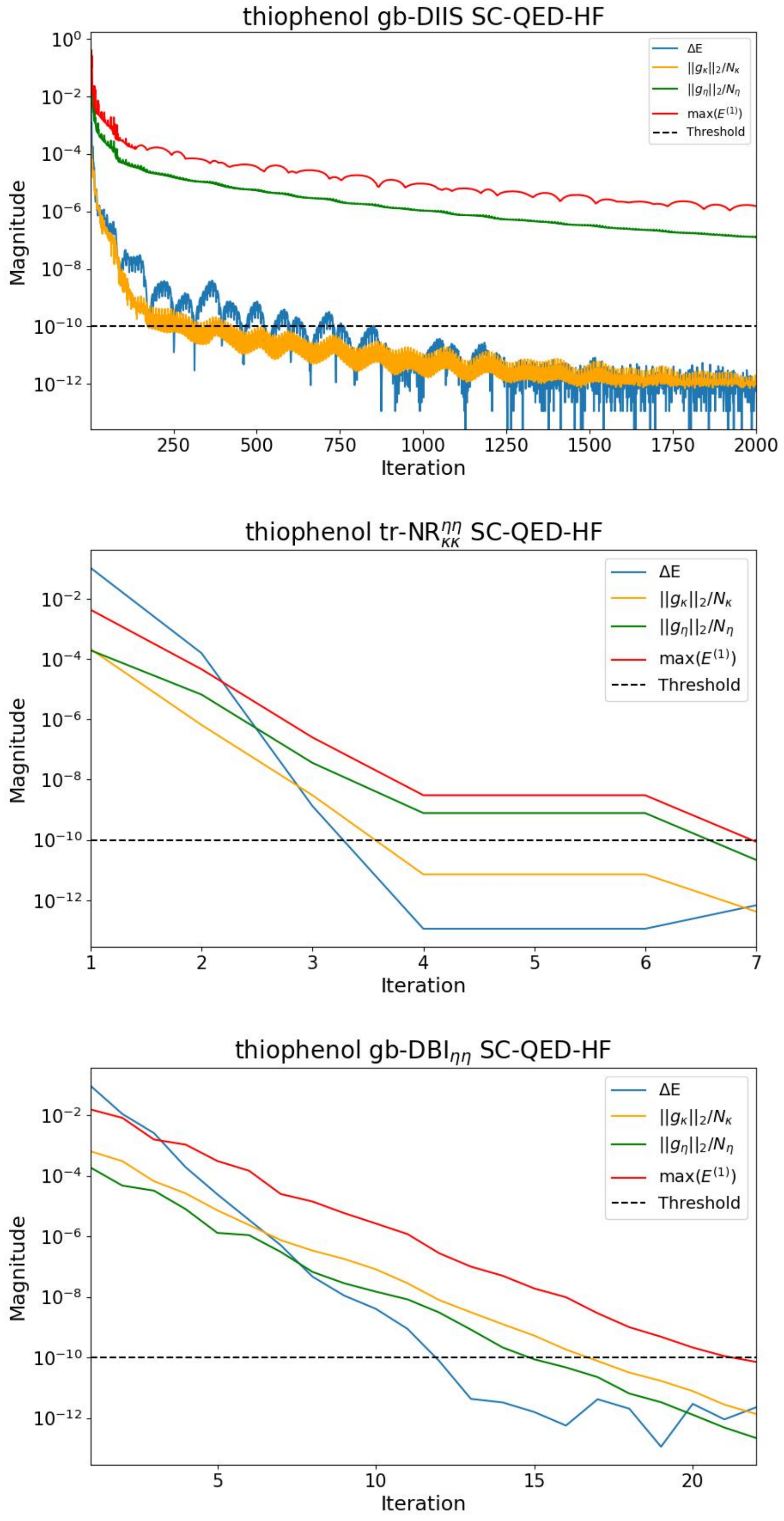

Figure S19: thiophenol convergence comparison

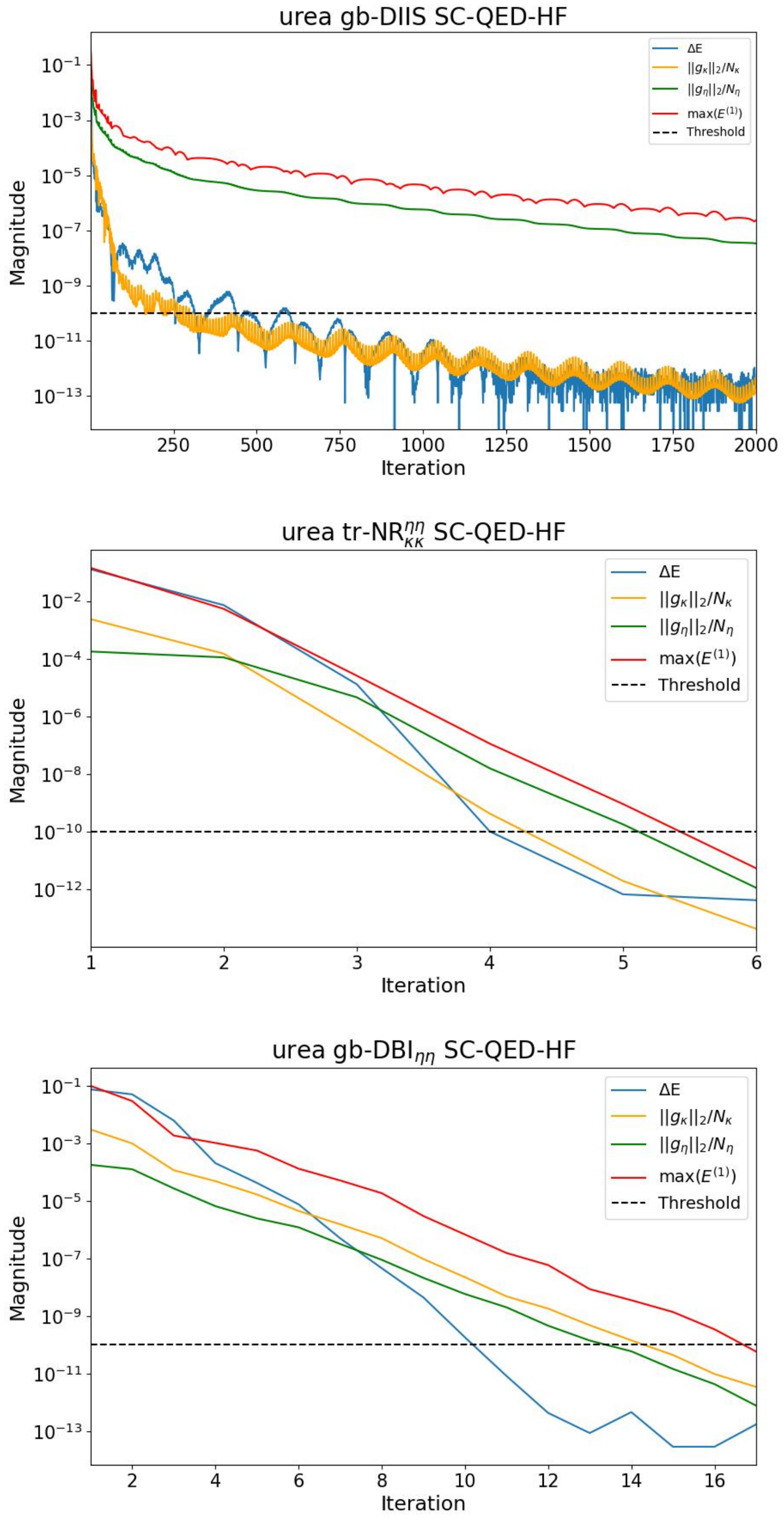

Figure S20: urea convergence comparison

S6 Hessian blocks analysis

The heat map representations of the κ-κ and η-η Hessian blocks are reported. The η-η Hessian blocks turn to be highly non-diagonal.

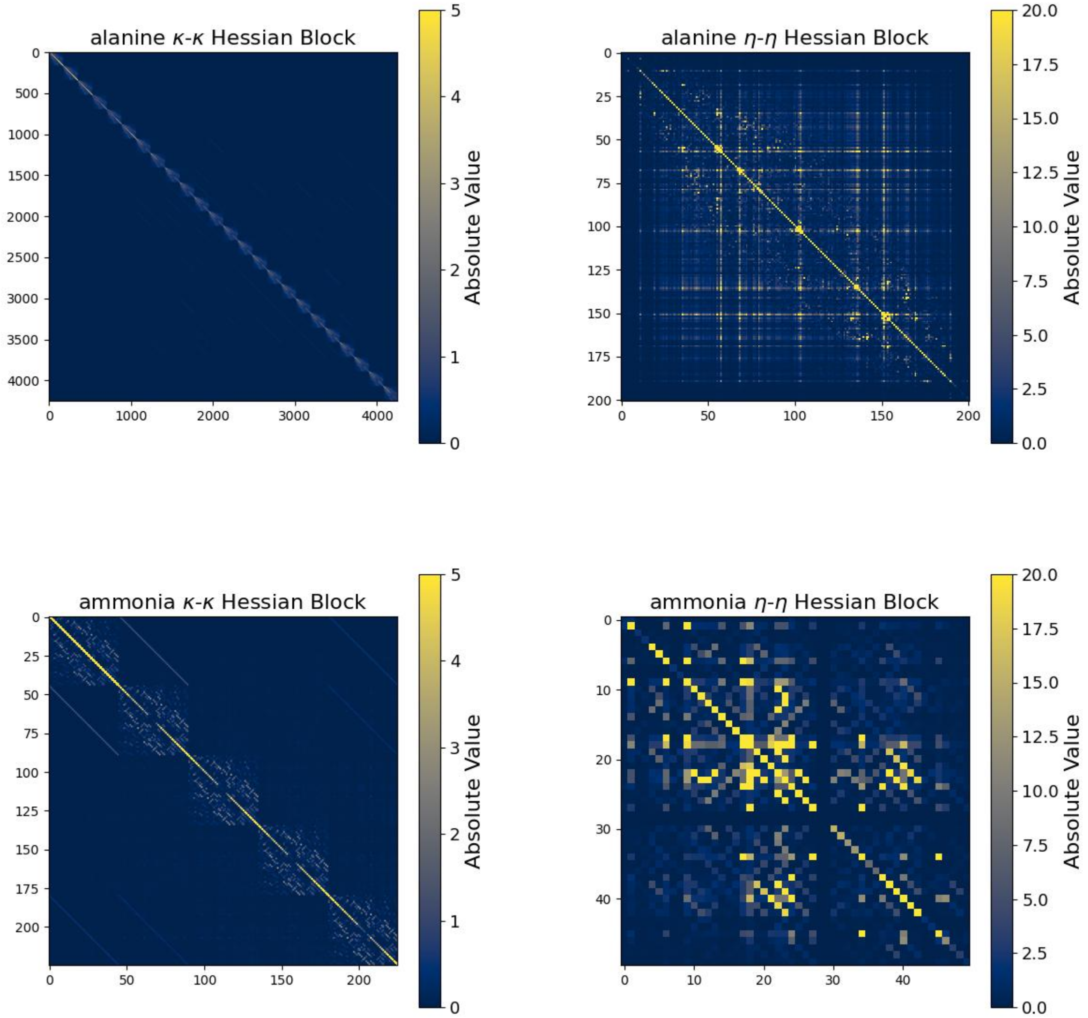

Figure S21: κ-κ and η-η Hessian heat map representations (part 1)

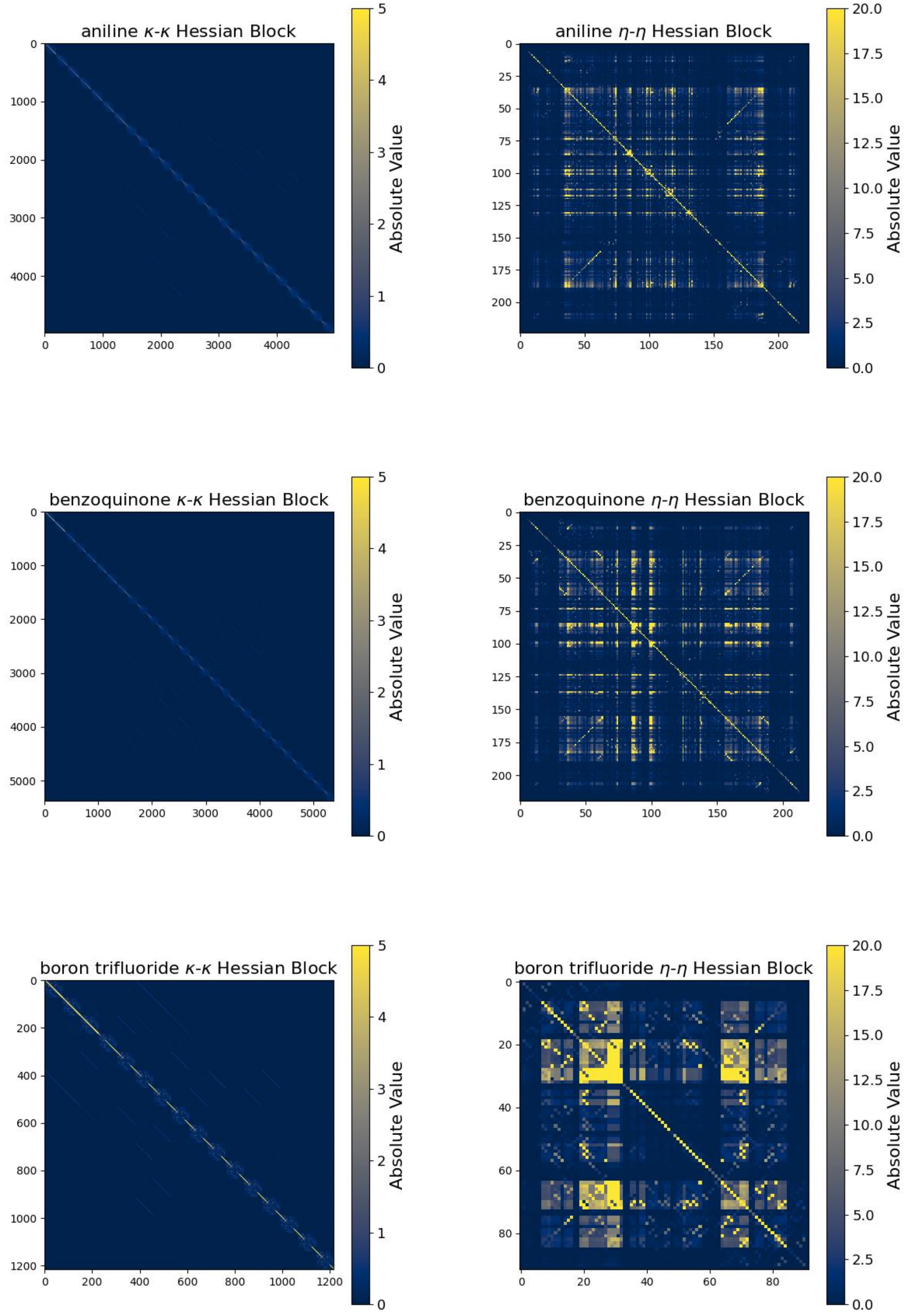

Figure S22: κ-κ and η-η Hessian heat map representations (part 2)

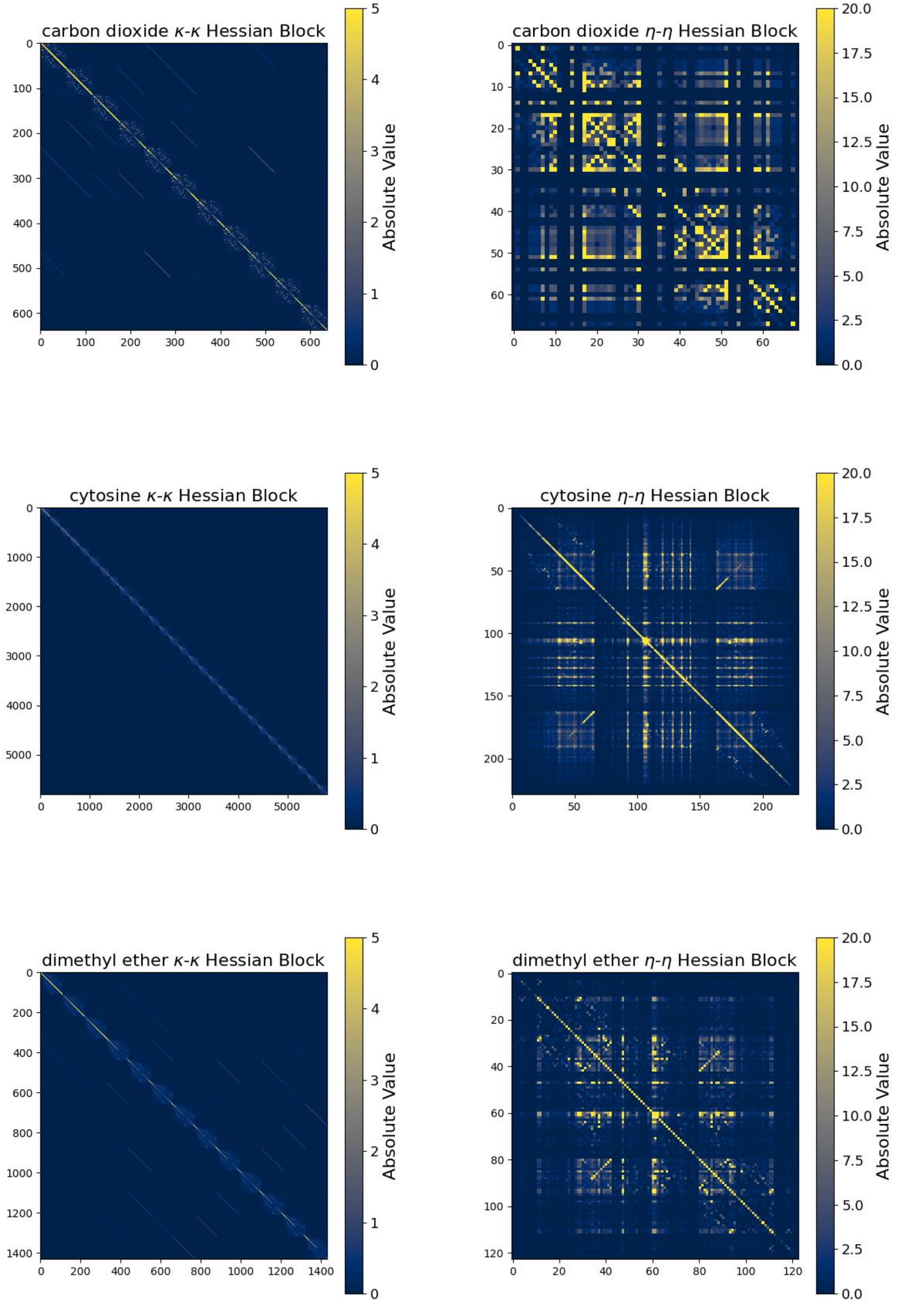

Figure S23: κ-κ and η-η Hessian heat map representations (part 3)

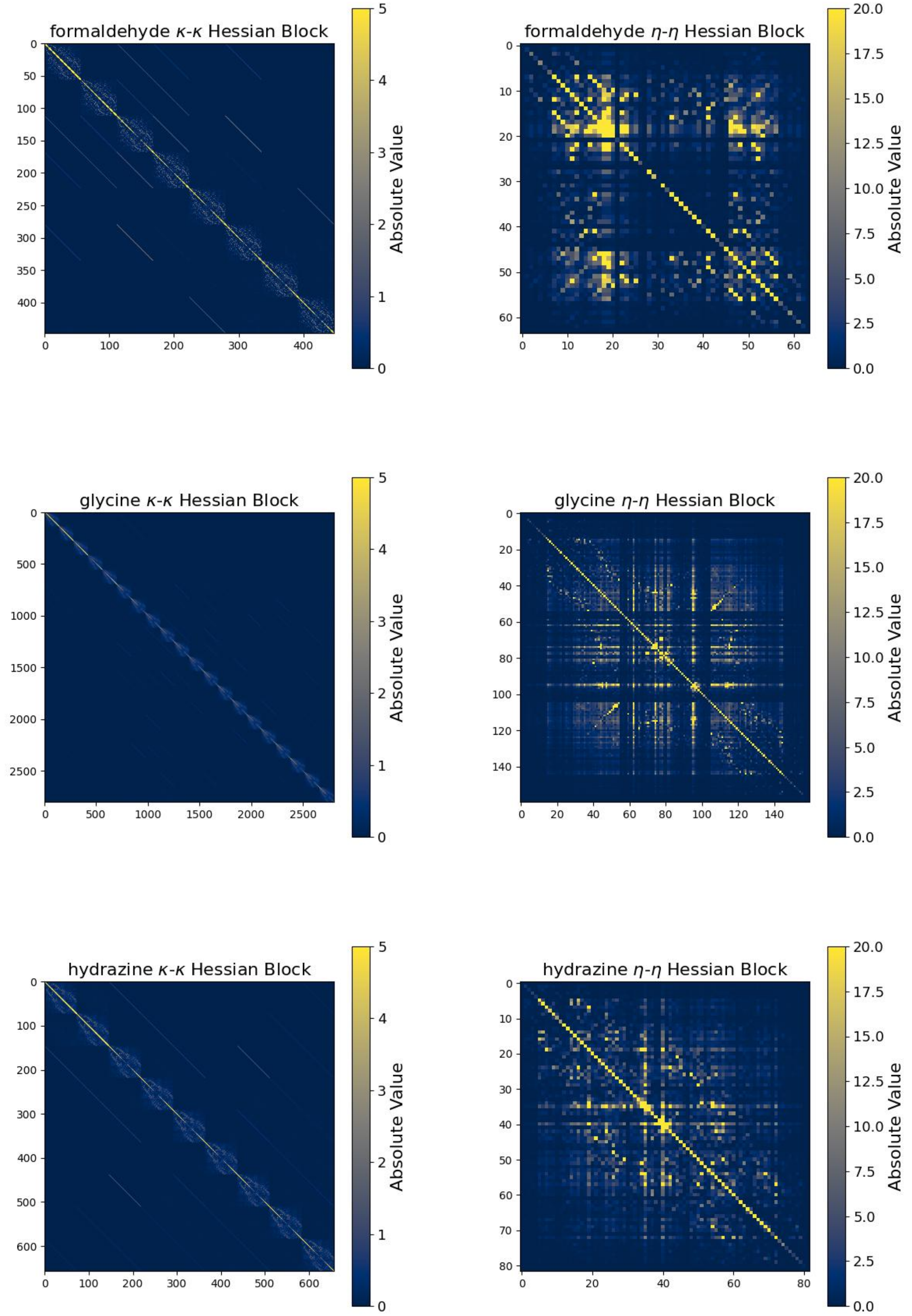

Figure S24: κ-κ and η-η Hessian heat map representations (part 4)

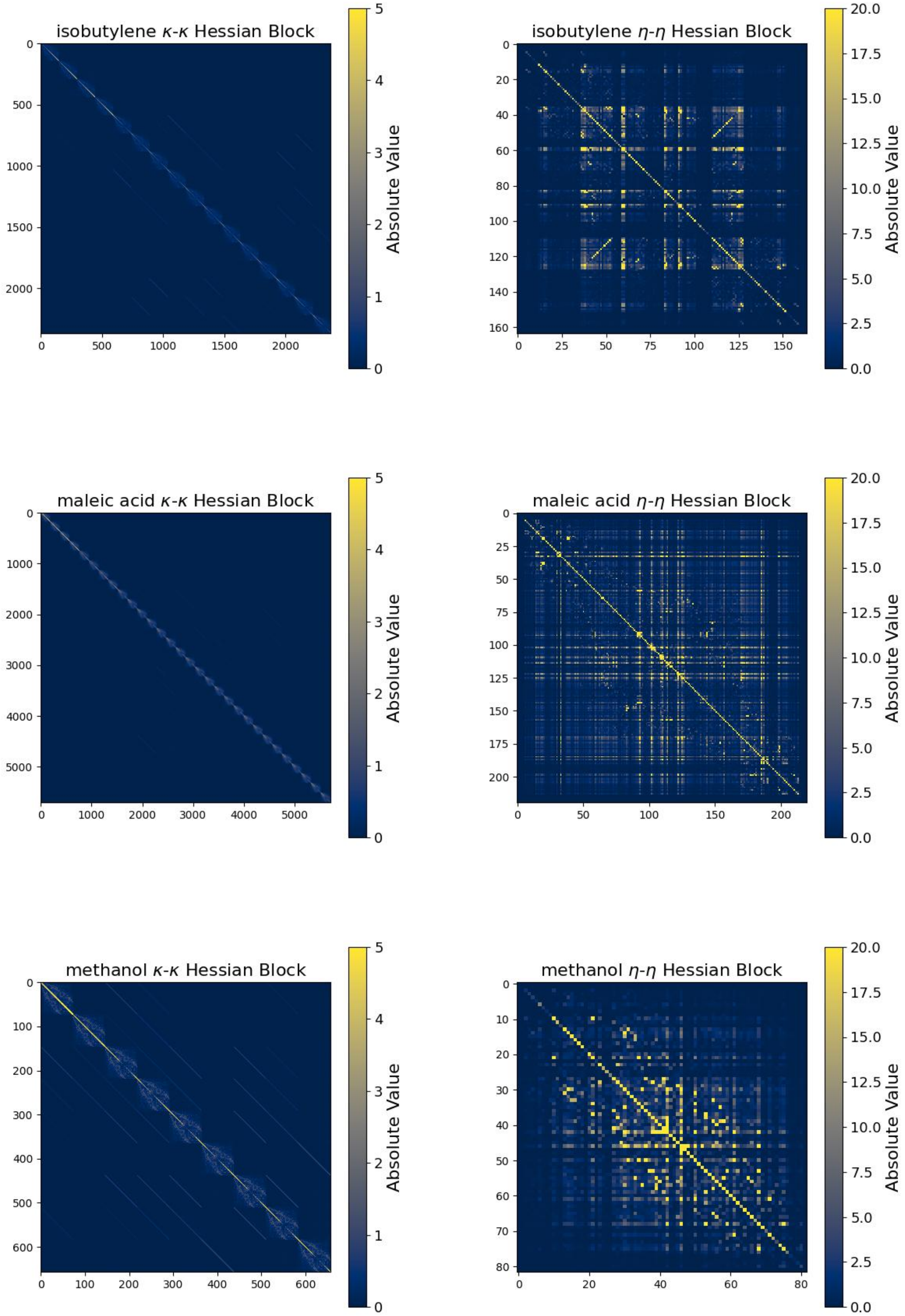

Figure S25: κ-κ and η-η Hessian heat map representations (part 5)

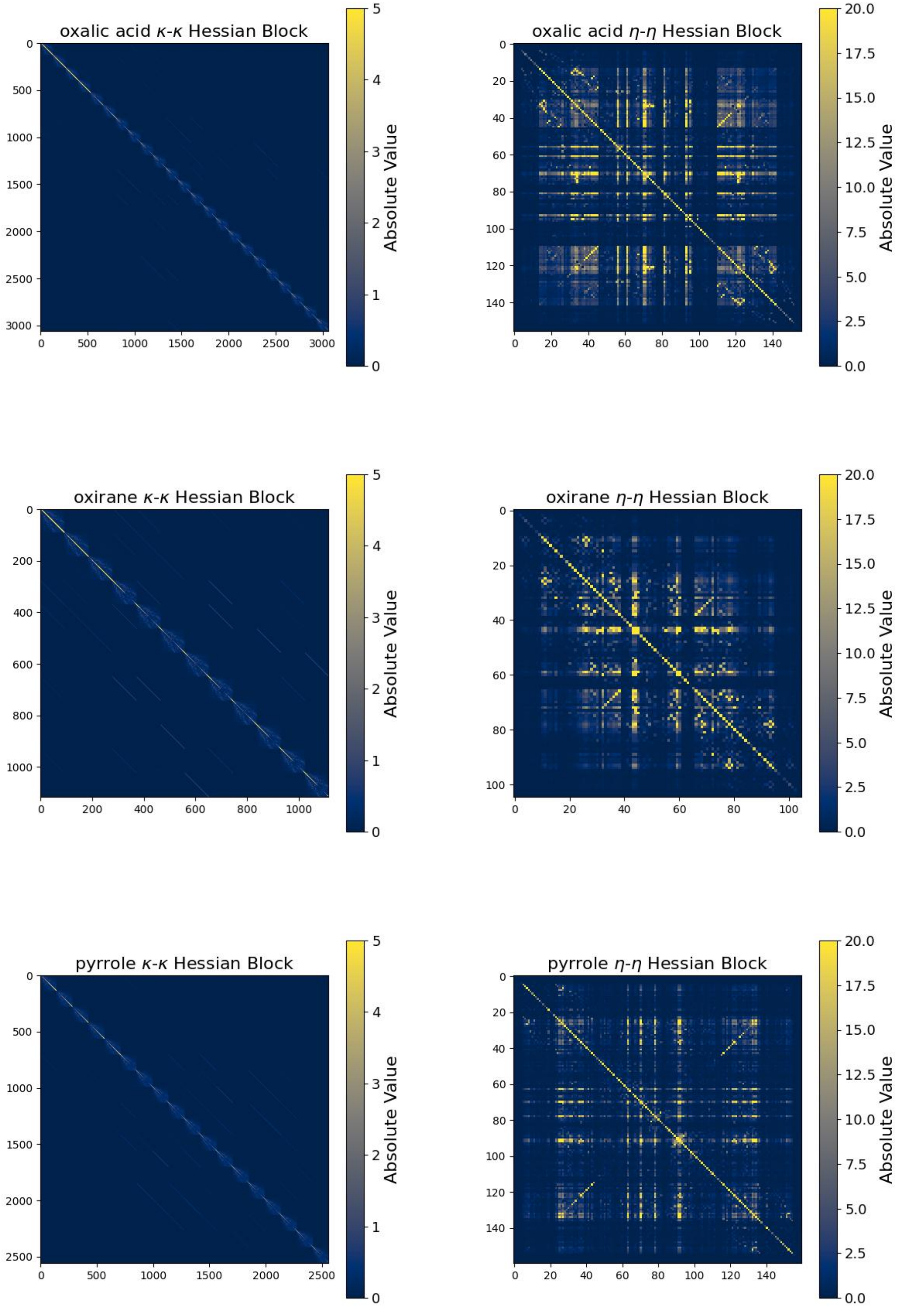

Figure S26: κ-κ and η-η Hessian heat map representations (part 6)

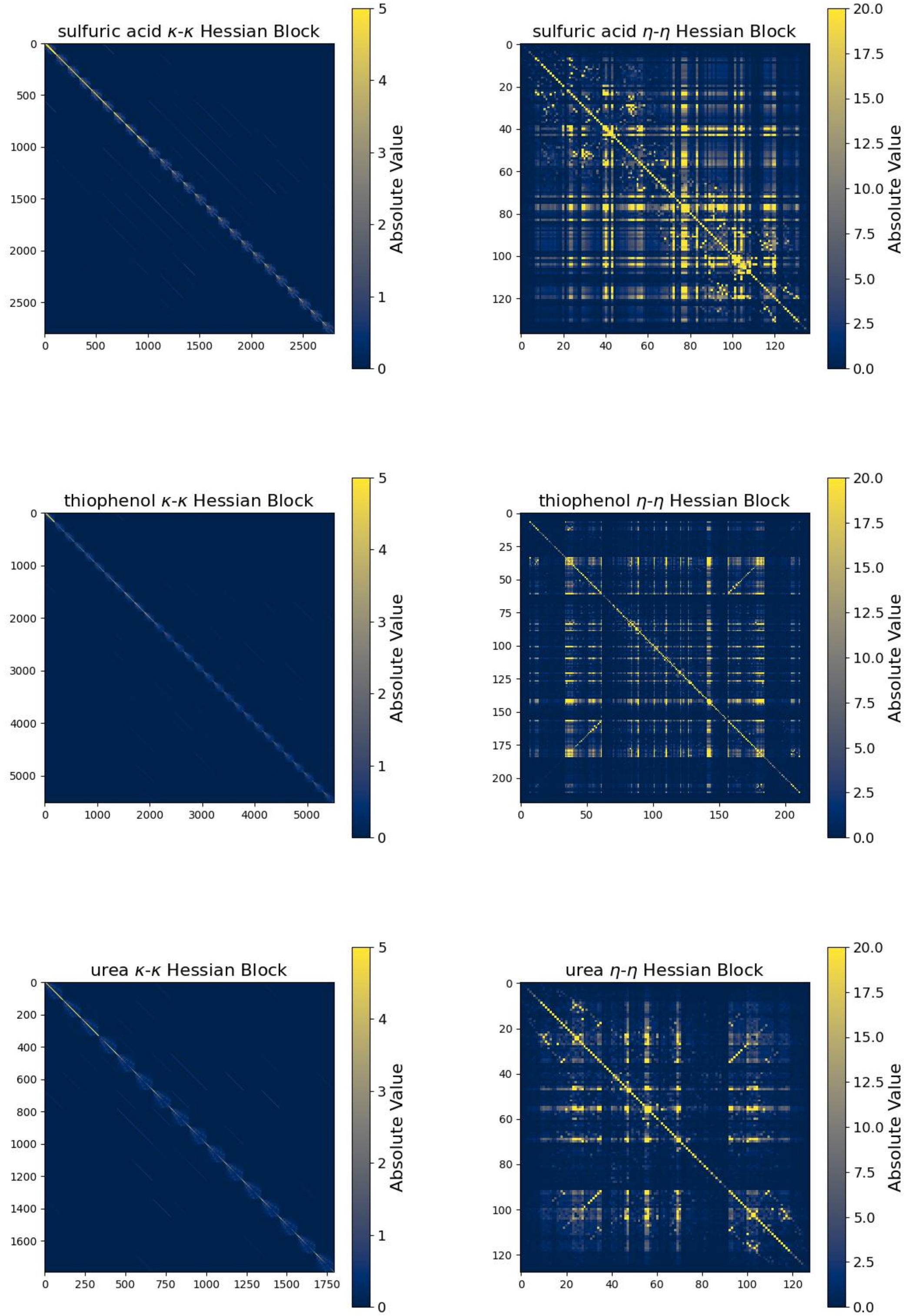

Figure S27: κ-κ and η-η Hessian heat map representations (part 7)

S7 Non-necessety of the mixed bloks

Table S1: Wall time comparison between the tr-NR with and without considering the mixed blocks.

Molecule	tr-NR$^{\mathfrak{m}}_{\kappa\kappa}$	full tr-NR	Molecule	tr-NR$^{\mathfrak{m}}_{\kappa\kappa}$	full tr-NR
alanine	22.0 m	178 m	hydrazine	26.8 s	2.07 m
ammonia	5.91 s	40.1 s	isobutylene	6.37 s	54.1 m
aniline	23.7 m	238 m	maleic acid	36.2 m	300 m
benzoquinone	25.1 m	203 m	methanol	31.2 s	2.53 m
boron trifluoride	69.1 s	6.38 m	oxalic acid	5.37 m	37.8 m
carbon dioxide	20.2 s	1.95 m	oxirane	83.3 s	7.65 m
cytosine	47.8 m	268 m	pyrrole	5.23 m	35.5 m
dimethyl ether	2.09 m	12.4 m	sulfuric acid	8.76 m	70.6 m
formaldehyde	10.2 s	51.6 s	thiophenol	26.0 m	193 m
glycine	6.94 m	46.5 m	urea	2.68 m	17.3 m

Considering the mixed parameters blocks significantly increase the computational time.

S8 Fullerene HOMO-1 and LUMO+1 reshapings

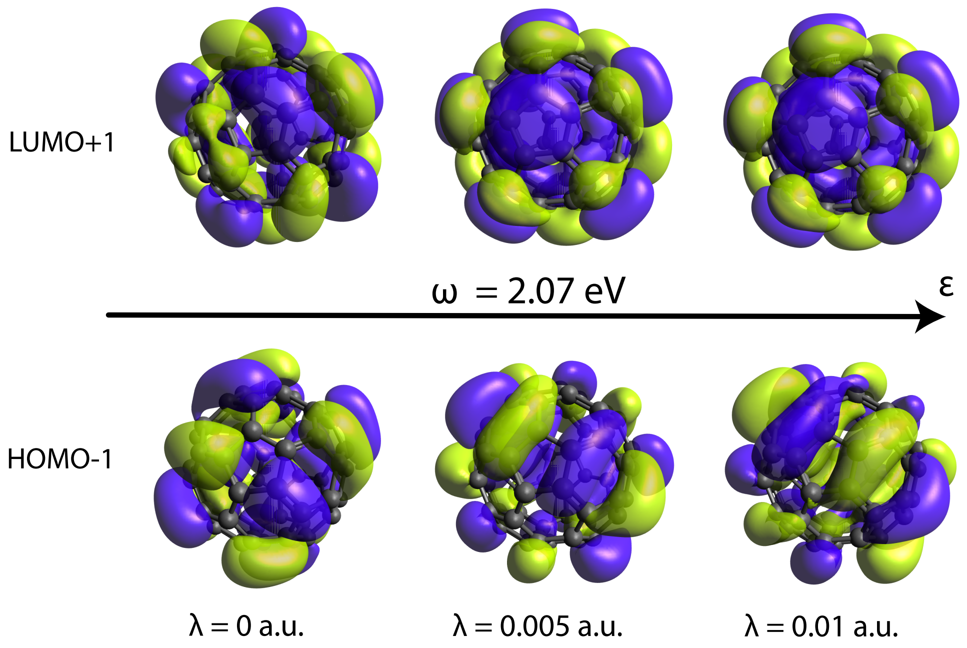

Figure S28: C_{60} HOMO-1 and LUMO+1 at various couplings and frequency $\omega = 2.07$ eV.

S9 Molecular geometries

S9.1 Benchmark molecules

Table S2: alanine

Atom	x / Å	y / Å	z / Å
O	1.50753958114233	-0.9029374701238	0.52054030256044
O	1.12221682236161	0.88332274641426	-0.78112521053633
N	-1.39540561214278	1.15983356228106	0.30075703084875
C	-0.74512877528783	-0.13676621947484	0.37697192544942
C	-1.36340268958411	-1.24559794663811	-0.50418028806638
C	0.71361012877607	0.03946686293961	-0.02223640526031
H	-0.75284178901618	-0.49045770582094	1.42313486090934
H	-0.83177433524497	-2.20336160316772	-0.38594943757755
H	-2.41677553294187	-1.40412648176633	-0.22104454535856
H	-1.33106130560111	-0.95414217030916	-1.56732063426856
H	-2.40884688413076	1.05498299638853	0.32109140324016
H	-1.14505244036518	1.6143346655389	-0.57924783924883
H	2.40272283203477	-0.75465123626146	0.16840883730841

Table S3: ammonia

Atom	x / Å	y / Å	z / Å
N	3.786477015e-05	1.529907129e-05	0.31539639529544
H	-0.46739972094214	-0.80950919569646	-0.10517015927472
H	-0.46742951902069	0.80951619955967	-0.10517027313719
H	0.93479137519269	-2.23029345e-05	-0.10515596288353

Table S4: aniline

Atom	x / Å	y / Å	z / Å
N	-2.39697479260141	-0.00014792296814	0.00275592032993
C	-1.0214390544481	-2.940659656e-05	0.00120960182057
C	-0.29450905196247	1.21072502088442	0.00079053568694
C	-0.29430055594105	-1.21066037017928	0.00081803715932
C	1.10009392661827	1.20342497869635	7.445541682e-05
C	1.10030178218867	-1.20312019059304	0.00010197259921
C	1.81432545418967	0.00021338511241	-0.00028619441974
H	-0.83629886730104	2.16180798159883	0.00110915183523
H	-0.83592412918912	-2.16183813269173	0.0011582159523
H	1.63659926728504	2.15687311088885	-0.00025432723956
H	1.63696612932574	-2.15647825037689	-0.00020462567067
H	2.90697576154526	0.00031134050761	-0.00090342650369
H	-2.91493638919997	-0.86575876011242	-0.00342178356454
H	-2.91507948050948	0.86537721582959	-0.00344753340211

Table S5: benzoquinone

Atom	x / Å	y / Å	z / Å
O	2.67084480116988	-5.221129452e-05	-0.0001191050417
O	-2.67089532135356	5.476825017e-05	-0.00028364430711
C	1.45178071465234	-2.999141965e-05	-0.00023061377989
C	-1.45183088256141	2.983846744e-05	0.00010175340409
C	-0.67278346054122	-1.26952063023965	-0.00013010974846
C	0.67268214997036	-1.26954701176556	-0.00019078381802
C	-0.67273172117467	1.26954679561817	-0.00012174526235
C	0.67273269375586	1.26951918333657	-0.00018139446467
H	-1.26393891328834	-2.18958008040806	-0.00017620771278
H	-1.2638486088068	2.18963104105478	-0.00016070144168
H	1.263800367527	-2.18963024259184	-0.00026205174719
H	1.26388818065058	2.18957854099213	-0.00024539608023

Table S6: boron trifluoride

Atom	x / Å	y / Å	z / Å
F	-1.14641330751968	-0.66138524600286	-2.500794e-08
F	1.14641332413075	-0.66138527062785	-2.500794e-08
F	-1.307361e-08	1.32290139350453	-2.498917e-08
B	-3.53746e-09	-0.00013087687382	7.500505e-08

Table S7: carbon dioxide

Atom	x / Å	y / Å	z / Å
C	5.81258e-09	-6.54397e-09	-1.039e-11
O	-2.90629e-09	3.27198e-09	1.16764877610542
O	-2.90629e-09	3.27198e-09	-1.16764877609503

Table S8: cytosine

Atom	x / Å	y / Å	z / Å
O	2.2134	1.1188	0.0001
N	-0.0595	0.9816	0.0001
N	-2.4422	0.9214	0.0001
N	1.2935	-0.9744	0.0000
C	-1.2406	0.2676	-0.0001
C	-1.1643	-1.0726	-0.0002
C	1.2088	0.4056	-0.0003
C	0.1908	-1.6478	0.0003
H	-0.1331	1.9946	0.0003
H	-2.0223	-1.7317	-0.0003
H	0.2351	-2.7487	0.0005
H	-3.3089	0.3984	0.0003
H	-2.4533	1.9332	0.0006

Table S9: dimethyl ether

Atom	x / Å	y / Å	z / Å
O	3.66128307e-06	0.51112290643144	-2.999968447e-05
C	1.17081361331246	-0.25673130886892	4.14728915e-06
C	-1.17079227694214	-0.25675265797246	-2.880408448e-05
H	1.24481186848303	-0.90869784880785	0.89651386167065
H	2.02852882602686	0.43241084109005	-1.03905564e-06
H	1.24483537822226	-0.90874145526379	-0.89647192938793
H	-1.24480567073375	-0.90871761620846	0.89648095516092
H	-1.24477566004411	-0.90876709802071	-0.89650515291614
H	-2.02851973960768	0.43237423762069	-6.203899206e-05

Table S10: formaldehyde

Atom	x / Å	y / Å	z / Å
O	2.0	-0.56	0.0
C	2.866	-0.06	0.0
H	3.403	-0.37	0.0
H	2.866	0.56	0.0

Table S11: glycine

Atom	x / Å	y / Å	z / Å
O	-1.64635674767162	0.69666778713316	-0.00334182128516
O	-0.61190647304033	-1.29751144451648	-0.00897361260389
N	1.95564054620037	-0.04998330502742	-0.00367325674711
C	0.73525913452538	0.72177975311312	0.00189850557701
C	-0.55335072638898	-0.09291280869991	-0.00419744441106
H	0.70062978735425	1.40127983531095	-0.86870516782063
H	0.70016139540602	1.38698180960039	0.88351855270123
H	1.97062545610749	-0.66416637873456	-0.81911636975786
H	1.96877634739106	-0.67848116135667	0.80085392619531
H	-2.42217871988366	0.10934591317741	-0.00676331184784

Table S12: hydrazine

Atom	x / Å	y / Å	z / Å
N	0.70860837953212	-0.03218959337804	-0.07356202980306
N	-0.7084990570299	-0.08023395228274	0.00283009152726
H	1.08800520911457	-0.32912410869742	0.82528846910161
H	1.09050966707597	-0.66641587032257	-0.78161491031548
H	-1.09054315549919	-0.99313749504662	-0.26207577244996
H	-1.08808104319356	0.6014010197274	-0.65396584806037

Table S13: isobutylene

Atom	x / Å	y / Å	z / Å
C	4.980472053e-05	-0.07528002952911	-7.654786715e-05
C	-1.27649632620912	0.72800835536229	-6.10075971e-05
C	1.27595031191592	0.72902672039391	-6.100321455e-05
C	0.00057799931762	-1.41577573781627	-0.00010916141183
H	-1.32792121764212	1.38901358275792	0.88429815741164
H	-1.32793614774829	1.38901964206031	-0.88441514151027
H	-2.16939711761342	0.08521717189449	-5.6654597e-05
H	1.32686005517176	1.39008316379982	-0.88441271879614
H	1.32684804165912	1.39007187625966	0.88429949699484
H	2.16936630029022	0.08695536543351	-5.972399418e-05
H	0.93396788960593	-1.9870598645323	-0.00012320228475
H	-0.93236959346814	-1.98778024608422	-0.00012249313352

Table S14: maleic acid

Atom	x / Å	y / Å	z / Å
O	1.66960729814715	-0.47043385601196	1.17854048038806
O	-2.79148310658494	-0.07256962639802	0.14873966666856
O	2.24098824521042	-0.20776764721045	-0.98150571517622
O	-0.9206553384183	-1.30340927363636	-0.04484066412311
C	0.56470300783914	1.21380268483585	-0.07098532165556
C	-0.76973998734556	1.09199943133127	-0.06507145354673
C	1.56078543804338	0.08980239955934	-0.03325034521337
C	-1.45876225140215	-0.22258974015487	0.0138443063211
H	1.01195465085034	2.20777110624459	-0.18202154486465
H	-1.40673456465497	1.97647897218841	-0.13906296708194
H	2.31646347928954	-1.1943174239794	1.09822378593623
H	-3.17212687097405	-0.96716702676839	0.18558977234764

Table S15: methanol

Atom	x / Å	y / Å	z / Å
H	0.7399715323734	-1.38235366551991	-0.33023236437175
O	0.62611968268962	-1.02887478951203	-1.22132136661531
C	0.036585032519	0.24528547286065	-1.11848590460926
H	0.67769368224915	0.9831542858221	-0.59457107143463
H	-0.9488144908505	0.23292209565651	-0.60894466780252
H	-0.12645543898066	0.61996660069268	-2.14134462516651

Table S16: oxalic acid

Atom	x / Å	y / Å	z / Å
O	1.27882218548722	-1.20774224846163	1.482722825e-05
O	-1.27882232783038	1.20776691042176	-2.13683262e-06
O	1.41293623399688	1.04139328928934	-7.9240558e-06
O	-1.41293612949503	-1.04136743034375	-0.00010073019257
C	0.77071305382101	0.02685660029597	8.002118598e-05
C	-0.77071311085077	-0.026831202192	0.00011364806898
H	2.24781230406452	-1.11679771160483	-6.04933413e-05
H	-2.24781220919346	1.11682179259513	-0.00013721206092

Table S17: oxirane

Atom	x / Å	y / Å	z / Å
O	-0.00058471720591	0.78395285441668	-4.322031081e-05
C	0.73516659848268	-0.42950894787735	1.21501162e-06
C	-0.73446927475546	-0.43063271496722	1.23329293e-06
H	1.27950649278931	-0.65953884020187	0.92676766588479
H	1.27950876432482	-0.65960557437501	-0.92674731378622
H	-1.27846504260857	-0.66156675999871	-0.92674308556764
H	-1.27846282102687	-0.66150001699653	0.92676350547533

Table S18: pyrrole

Atom	x / Å	y / Å	z / Å
N	0.0032	-1.158	-0.0002
C	-1.1187	-0.3708	0.0002
C	1.1207	-0.3648	0.0001
C	-0.711	0.9448	-0.0002
C	0.7058	0.9487	0.0
H	0.006	-2.1687	-0.0003
H	-2.1038	-0.8157	0.0003
H	2.1083	-0.8041	0.0002
H	-1.3638	1.8064	-0.0003
H	1.3538	1.8139	0.0

Table S19: sulfuric acid

Atom	x / Å	y / Å	z / Å
S	-5.625152911e-05	3.206172935e-05	0.00233411221782
O	1.26952591394871	-0.01615530145921	1.04809977400958
O	-1.27215920339628	0.01948359622497	1.0449629206123
O	-0.02221283777656	1.29786308832917	-0.67132745181754
O	0.02372057611632	-1.29991717076664	-0.66716375198493
H	1.46506005138462	0.91826666614824	1.25259148394876
H	-1.4676782487477	-0.91427294020588	1.25250291301401

Table S20: thiophenol

Atom	x / Å	y / Å	z / Å
S	-2.72866564544314	-0.04663050980193	-0.0013273473747
C	-0.94869923052328	0.02285092621988	-0.00079969870173
C	-0.22744247421651	1.22799267170112	-0.00065877989291
C	-0.24761404088989	-1.19558962937262	-0.00034597420843
C	1.16982352700033	1.20991880782021	-0.00010353014917
C	1.14858243982194	-1.20271352793089	0.00027091857853
C	1.86626857728308	-0.00217394824555	0.00038737331671
H	-0.75635975989472	2.18487618929624	-0.00096157150485
H	-0.79731648561778	-2.14092480415403	-0.00051166162546
H	1.71665630536956	2.15695347197674	-2.666117455e-05
H	1.67911777011471	-2.15894015335134	0.00063478709336
H	2.95923625025082	-0.01148087864139	0.0008602545019
H	-2.94218723325511	1.28806138448354	-0.0028181088587

Table S21: urea

Atom	x / Å	y / Å	z / Å
O	-1.25559968098163	-0.00081369063439	1.583804951e-05
N	0.70496806919806	-1.16029577309685	0.0004114573546
N	0.70344025576134	1.16124801359111	0.00016757054398
C	-0.03727089884835	-1.051746228e-05	0.00015841205984
H	1.71480091460371	-1.18903947966413	-0.00043723162763
H	0.19204822255911	-2.03078766413551	-0.00019274388568
H	0.18937808019146	2.03106585740785	-0.00016546686953
H	1.7132350375163	1.1913332539942	0.00014216437491

S9.2 Large molecular systems

Table S22: fullerene (C_{60})

Atom	x / Å	y / Å	z / Å	Atom	x / Å	y / Å	z / Å
C	3.2742	-1.4297	-0.062	C	1.104	0.9078	-3.275
C	3.5714	-0.0192	-0.085	C	0.6762	2.9475	-1.9021
C	3.3119	0.7909	1.0789	C	0.266	3.5283	0.493
C	2.7182	-2.0292	1.1233	C	-0.6344	2.5573	2.4125
C	2.7702	-1.799	-1.3612	C	-0.6207	1.3752	3.2375
C	3.2507	0.4831	-1.3992	C	-1.1802	-3.3171	0.6015
C	2.7332	2.1042	0.93	C	-1.5988	-2.0068	2.487
C	2.7559	0.1913	2.2642	C	-1.104	-0.9078	3.275
C	1.6574	-2.9989	1.0111	C	-1.819	-2.3154	-2.0245
C	2.4586	-1.2192	2.2872	C	-0.612	-0.6637	-3.4559
C	1.7093	-2.7687	-1.4734	C	-0.3141	0.748	-3.4792
C	2.7559	-0.6159	-2.1872	C	-0.742	2.7877	-2.1063
C	2.6725	1.7952	-1.5485	C	-1.1533	3.3682	0.2881
C	1.819	2.3154	2.0245	C	-1.7093	2.7687	1.4734
C	2.4129	2.6056	-0.3832	C	-1.6822	0.4043	3.1256
C	1.8327	1.1333	2.8495	C	-2.4129	-2.6056	0.3832
C	1.1533	-3.3682	-0.2881	C	-2.6725	-1.7952	1.5485
C	0.742	-2.7877	2.1063	C	-2.7332	-2.1042	-0.93
C	1.2367	-1.6887	2.8943	C	-1.8327	-1.1333	-2.8495
C	0.6344	-2.5573	-2.4125	C	-1.2367	1.6887	-2.8943
C	1.6822	-0.4043	-3.1256	C	-1.6574	2.9989	-1.0111
C	1.5988	2.0068	-2.487	C	-2.7702	1.799	1.3612
C	0.5863	3.027	1.8062	C	-2.7559	0.6159	2.1872
C	1.1802	3.3171	-0.6015	C	-3.2507	-0.4831	1.3992
C	0.612	0.6637	3.4559	C	-3.3119	-0.7909	-1.0789
C	-0.266	-3.5283	-0.493	C	-2.7559	-0.1913	-2.2642
C	-0.6762	-2.9475	1.9021	C	-2.4586	1.2192	-2.2872
C	0.3141	-0.748	3.4792	C	-2.7182	2.0292	-1.1233
C	-0.5863	-3.027	-1.8062	C	-3.2742	1.4297	0.06200
C	0.6207	-1.3752	-3.2375	C	-3.5714	0.0192	0.08500

Table S23: heme group + proximal histidine + oxygen (part 1)

Atom	x / Å	y / Å	z / Å
C	0.949	-0.672	2.746
C	3.001	-0.026	-0.864
C	-0.728	1.063	-2.683
C	-2.887	0.191	0.819
C	2.112	-0.964	3.564
C	3.854	0.323	-1.985
C	-1.861	1.466	-3.502
C	-3.701	-0.049	1.964
C	3.175	-0.898	2.724
C	3.043	0.761	-3.001
C	-2.984	1.359	-2.742
C	-2.897	-0.431	2.951
C	2.670	-0.576	1.449
C	1.702	0.668	-2.443
C	-2.539	0.890	-1.465
C	-1.553	-0.434	2.442
C	2.046	-1.695	4.893
C	3.279	0.897	-4.500
C	-4.398	1.734	-3.083
C	-3.357	-0.622	4.368
C	2.937	-2.893	5.152
C	4.540	0.931	-5.105
C	-4.759	3.038	-3.338
C	-3.371	0.660	5.191
C	2.763	-3.227	6.631
C	-3.900	0.294	6.560
C	-0.396	-0.670	3.217
C	3.470	-0.472	0.347
C	0.587	1.044	-3.154
C	-3.377	0.641	-0.417
C	4.644	-0.985	2.991
C	5.317	-0.011	-1.992
C	-1.804	1.605	-4.990

Atom	x / Å	y / Å	z / Å
C	-5.064	0.527	2.188
Fe	0.000	0.000	0.000
N	1.323	-0.382	1.472
N	1.683	0.205	-1.128
N	-1.139	0.720	-1.413
N	-1.582	-0.078	1.101
O	0.230	1.577	0.449
O	3.715	-3.147	7.419
O	-4.964	-0.317	6.611
O	-0.080	2.755	0.461
O	1.625	-3.510	6.985
O	-3.229	0.602	7.548
C	-3.434	-6.471	-2.220
C	-2.458	-5.739	-1.297
C	-1.476	-4.938	-2.117
C	-1.085	-2.512	-1.284
C	0.646	-2.838	0.073
C	-0.775	-3.834	-1.314
N	-1.678	-6.660	-0.420
N	0.293	-4.064	-0.458
N	-0.206	-1.875	-0.439
O	-4.517	-5.941	-2.435
H	1.037	-2.032	5.009
H	2.409	-0.967	5.588
H	2.768	0.070	-4.948
H	2.956	1.904	-4.660
H	-5.117	0.995	-3.127
H	-2.701	-1.321	4.844
H	-4.367	-0.972	4.322
H	3.958	-2.651	4.942
H	2.675	-3.721	4.527
H	5.001	-0.031	-5.024
H	5.151	1.661	-4.616

Table S24: heme group + proximal histidine + oxygen (part 2)

Atom	x / Å	y / Å	z / Å
H	4.433	1.189	-6.138
H	-4.050	3.787	-3.297
H	-5.738	3.266	-3.570
H	-4.009	1.386	4.731
H	-2.391	1.084	5.259
H	-0.538	-0.859	4.221
H	4.464	-0.738	0.427
H	0.736	1.341	-4.131
H	-4.392	0.787	-0.536
H	5.180	-0.890	2.070
H	4.870	-1.930	3.439
H	4.933	-0.197	3.655
H	5.757	0.332	-2.905
H	5.441	-1.071	-1.912
H	5.796	0.468	-1.164
H	-2.761	1.912	-5.357
H	-1.540	0.664	-5.426
H	-1.070	2.338	-5.252
H	-5.514	0.756	1.245
H	-4.982	1.421	2.770
H	-5.670	-0.184	2.710
H	4.569	-2.927	7.102
H	-2.424	1.069	7.440
H	-3.056	-5.108	-0.673
H	-0.732	-5.604	-2.501
H	-2.030	-4.460	-2.898
H	-1.100	-7.271	-0.997
H	-2.320	-7.225	0.136
H	0.717	-4.932	-0.266
H	1.416	-2.669	0.739
H	-1.852	-2.061	-1.807
O	-3.255	-7.537	-2.712
H	-2.747	-8.355	-2.711
